# Lifestyle Transitions in Fusarioid Fungi are Frequent and Lack Clear Genomic Signatures

**DOI:** 10.1093/molbev/msac085

**Published:** 2022-04-29

**Authors:** Rowena Hill, Richard J.A. Buggs, Dang Toan Vu, Ester Gaya

**Affiliations:** 1 Comparative Fungal Biology, Royal Botanic Gardens Kew, Jodrell Laboratory, Richmond, United Kingdom; 2 School of Biological and Behavioural Sciences, Queen Mary University of London, London, UK; 3 Research Planning and International Cooperation Department, Plant Resources Center, Hanoi, Vietnam

**Keywords:** *Fusarium*, fungal endophytes, plant pathogens, lifestyle evolution, effectors, CAZymes

## Abstract

The fungal genus *Fusarium* (Ascomycota) includes well-known plant pathogens that are implicated in diseases worldwide, and many of which have been genome sequenced. The genus also encompasses other diverse lifestyles, including species found ubiquitously as asymptomatic-plant inhabitants (endophytes). Here, we produced structurally annotated genome assemblies for five endophytic *Fusarium* strains, including the first whole-genome data for *Fusarium chuoi*. Phylogenomic reconstruction of *Fusarium* and closely related genera revealed multiple and frequent lifestyle transitions, the major exception being a monophyletic clade of mutualist insect symbionts. Differential codon usage bias and increased codon optimisation separated *Fusarium sensu stricto* from allied genera. We performed computational prediction of candidate secreted effector proteins (CSEPs) and carbohydrate-active enzymes (CAZymes)—both likely to be involved in the host–fungal interaction—and sought evidence that their frequencies could predict lifestyle. However, phylogenetic distance described gene variance better than lifestyle did. There was no significant difference in CSEP, CAZyme, or gene repertoires between phytopathogenic and endophytic strains, although we did find some evidence that gene copy number variation may be contributing to pathogenicity. Large numbers of accessory CSEPs (i.e., present in more than one taxon but not all) and a comparatively low number of strain-specific CSEPs suggested there is a limited specialisation among plant associated *Fusarium* species. We also found half of the core genes to be under positive selection and identified specific CSEPs and CAZymes predicted to be positively selected on certain lineages. Our results depict fusarioid fungi as prolific generalists and highlight the difficulty in predicting pathogenic potential in the group.

## Introduction

*Fusarium* (Hypocreales, Ascomycota) is a globally distributed genus of approximately 230 species (https://www.fusarium.org/), many of which are implicated in devastating fungal diseases of plants. For instance, throughout the first half of the 20th century, Fusarium wilt of banana single-handedly wiped out the main globally traded banana cultivar—equivalent to losses of at least US$2.3 billion in 2000 (Ploetz 2005). A new *Fusarium* epidemic is now affecting the current dominant banana cultivar (Ordonez et al. 2015). Moreover, on the much-cited list of the top 10 fungal plant pathogens by Dean et al. (2012), two spots belong to *Fusarium* species. Beyond plant pathogenicity, however, many species are also reported to exhibit an array of other fungal lifestyles (see supplementary table S1, Supplementary Material online), and *Fusarium* strains are also frequently isolated from inside healthy plant tissues (e.g., Parsa et al. 2016; Zakaria and Aziz 2018; Rashmi et al. 2019). Fungal inhabitants of plant tissues which cause no symptoms of disease are known as fungal endophytes, hyperdiverse microfungi that are omnipresent in plant microbiomes (Rodriguez et al. 2009; Hardoim et al. 2015).

There is no single role that endophytes play in the plant host, as the endophytic lifestyle represents a functional range between pathogenicity and mutualism, which has been dubbed the “endophytic continuum” (Schulz and Boyle 2005). The outcome of endophyte colonisation can be highly dependent on the context of the plant–fungal interaction, such as the status of the plant immune system and nutrient conditions (Junker et al. 2012; Lahrmann et al. 2015; Hacquard et al. 2016; Hiruma et al. 2016), as well as the presence of other endophytes within the microbiome (Redman et al. 2001; Durán et al. 2018; Mesny et al. 2021; Wolinska et al. 2021) and even light conditions (Álvarez-Loayza et al. 2011). The transient status of endophytism for many taxa is evident from observations of endophytes becoming decayers (saprotrophs) or pathogens following some change in host or abiotic conditions (Slippers and Wingfield 2007; Arnold et al. 2009; Promputtha et al. 2010; Swett and Gordon 2015; Nelson et al. 2020). In some cases, however, an evolutionary transition from pathogenicity to endophytism may represent a permanent switch to obligate commensalism or mutualism (Gazis et al. 2016), and it has also been hypothesised that endophytism may have been an ancestral “waiting room” for the evolution of mycorrhizal symbiosis (Selosse et al. 2018).

The need to categorise pathogenic potential of *Fusarium* taxa is obvious considering the ubiquity of *Fusarium* endophytes in our crops (e.g., Rubini et al., 2005; Leoni et al., 2013; Sandoval-Denis et al., 2018) and the ramifications of pathogenic *Fusarium* strains for food security (e.g., Menzies et al. 1990; Kokkonen et al. 2010; Okello et al. 2020). In >200 years since *Fusarium* was first described, the generic concept has been the source of lively debate (Summerell 2019). In recent years, many *Fusarium* species complexes have been reclassified into distinct “fusarioid” genera based on phenotypic and phylogenetic evidence—such as *Albonectria*, *Bisifusarium*, *Cyanonectria*, *Geejayessia*, *Neocosmospora*, and *Rectifusarium* (Schroers et al. 2011; Lombard et al. 2015; Sandoval-Denis et al. 2019)—resulting in a narrower definition of the genus, *Fusarium sensu stricto*. This has been opposed in some quarters, with the argument that retaining a broader definition of the genus (*Fusarium sensu lato*) is desirable to facilitate communication between scientists and practitioners dealing with agriculturally and clinically important species that have historically been classified under *Fusarium* (Geiser et al. 2021; O’Donnell et al. 2020). Crous, Lombard, et al. (2021) countered that, in light of ever-increasing species discovery and recognised chemical and morphological differences between these clades, reclassification of certain species complexes into different genera is both biologically and practically meaningful. However, both sides of the debate note that ecology is similar among many of these taxa, and so questions regarding lifestyle warrant a perspective that includes allied fusarioid genera.

An evolutionary genomics approach using genomes from diverse lifestyles of fusarioid fungi could address this issue of detecting where strains fall on the pathogenic-mutualistic spectrum. A phylogenomic framework could not only shed light on the timing and frequency of lifestyle transitions in the group, but also inform to what extent genetic content is shared between taxa due to ancestry versus lifestyle. In addition to comparing gene repertoires, detecting signatures of selection may also help to uncover the genetic basis of recently evolved traits. Methods based on the ratio of nonsynonymous to synonymous substitutions (dN/dS) and the phenomenon of codon usage bias—where certain codons appear more frequently than others despite encoding the same amino acid—can be used to investigate the extent of selection acting on gene content.

One genetic feature that can be particularly illuminating to compare between lifestyles is genes that encode effector proteins. Fungal effectors (known as candidate-secreted effector proteins [CSEPs] when computationally predicted) are small secreted proteins produced by fungi which mediate the plant–fungal interaction. While best-studied in the context of pathogenicity (Stergiopoulos and de Wit 2009; De Jonge et al. 2011), we now know that effectors are also essential for mutualistic or commensal fungi to form associations with plant hosts by evading the host immune response (Rafiqi et al. 2012; Plett and Martin 2015; Lo Presti et al. 2015). Effector repertoires have been shown to differentiate host-specific strains (*forma specialis*) in the *Fusarium oxysporum* species complex (FOSC) (van Dam et al. 2016), and could potentially further distinguish pathogenic and endophytic FOSC strains (Czislowski et al. 2021). Another frequently studied group of proteins involved in the plant–fungal interaction are carbohydrate-active enzymes (CAZymes), many of which act as plant cell wall–degrading enzymes (PCWDEs) (Kubicek et al. 2014). CAZymes are often referred to as saprotrophic features (Lebreton et al. 2021), but are also abundant in plant pathogens and endophytes (e.g., Zhao et al. 2013; Knapp et al. 2018; Mesny et al. 2021), and, although present in lower numbers in mycorrhizal fungi (Kohler et al. 2015; Peter et al. 2016; Miyauchi et al. 2020), certain CAZymes play key roles in the establishment and maintenance of the symbiosis (Veneault-Fourrey et al. 2014; Doré et al. 2017; Marqués-Gálvez et al. 2021). Comparing CSEP and CAZyme repertoires is therefore highly relevant to exploring genetic differences in plant associated lifestyles of fusarioid fungi.

Here, we performed whole genome sequencing, assembly, and structural annotation of five novel endophytic *Fusarium* strains (supplementary table S2, Supplementary Material online), including the first whole genome sequencing data and annotated assembly for the recently described species, *Fusarium chuoi* (Crous, Osieck, et al. 2021). Using predicted genes from these and other publicly available fusarioid strains, we produced a genome-scale phylogeny of *Fusarium* and allied genera with time calibration and compared CSEP and CAZyme content to answer the following questions: 1) How are lifestyles distributed across the phylogeny? 2) Can we distinguish plant pathogens and endophytes from genome sequences alone? and 3) How is selection acting on different lifestyles?

## Results

### Both Single- and Multi-Copy Genes Inferred the Same Backbone for *Fusarium s. str*.

**Fig. 1.**
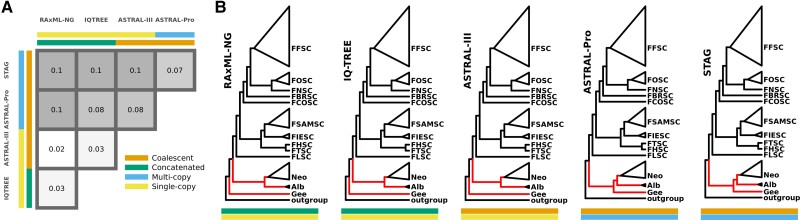
(*A*) Pairwise comparison of normalised Robinson–Foulds distances between topologies from all species tree estimation methods, with grid cells coloured from most similar topology (lighter) to most dissimilar (darker). (*B*) Summary of species trees with red branches indicating topological discordance between methods. Labels indicate species complex (SC) or allied genus (FFSC = *Fusariumfujikuroi* species complex, FOSC = *Fusarium oxysporum* species complex, FNSC = *Fusarium nisikadoi* species complex, FBRSC = *Fusarium burgessii* species complex, FCOSC = *Fusarium concolor* species complex, FSAMSC = *Fusarium sambucinum* species complex, FIESC = *Fusarium incarnatum-equiseti* species complex, FHSC = *Fusarium heterosporum* species complex, FTSC = *Fusarium tricinctum* species complex, FLSC = *Fusarium lateritium* species complex, Neo = *Neocosmospora*, Alb = *Albonectria*, Gee = *Geejayessia*).

To infer the genome-scale phylogeny of *Fusarium*, we used both concatenation and coalescent-based approaches, using single-copy genes with and without multi-copy genes also included (see our bioinformatics pipeline in supplementary fig. S1, Supplementary Material online). Including multi-copy genes had a greater impact on topology than tree building approach (i.e., concatenation versus coalescent) (fig. 1*A*). This was seen chiefly from a change in divergence order of allied genera—*Neocosmospora* (= *Fusarium solani* species concept, “FSSC”), *Geejayessia* (= *Fusarium staphyleae* species concept, “FSTSC”), and *Albonectria* (= *Fusarium decemcellulare* species concept, “FDESC”)—when including multi-copy genes (fig. 1*B*). All methods, however, produced the same divergence order for *Fusarium s. str* species concepts. Disregarding differences in the naming of species, our estimations of *Fusarium s. str* from 1,060 loci were in broad agreement with the most recent phylogenetic analyses by Crous, Lombard, et al. (2021) and Geiser et al. (2021).

We additionally compared the impact of alignment trimming tools—trimAl (Capella-Gutiérrez et al. 2009) versus BMGE (Criscuolo and Gribaldo 2010)—on species tree topology. The RAxML-NG (Kozlov et al. 2019) species tree was identical for both trimming tools, but trimming tool impacted topology for IQ-TREE (Minh et al. 2020) and ASTRAL-III (Zhang, Rabiee, et al. 2018), with discordance in the ambrosia clade of *Neocosmospora* (supplementary fig. S2, Supplementary Material online). The gene trees trimmed with trimAl were selected for downstream analyses based on its reported accuracy relative to BMGE in the literature (Tan et al. 2015; Steenwyk et al. 2020). The RAxML-NG species tree was selected for downstream analyses as its topology was identical for both trimming tools while having branch length units as substitutions per site as opposed to coalescent units.

### Dated Genome-Scale Phylogeny of *Fusarium* and Allied Genera

For divergence time estimation of the RAxML-NG species tree, we used both the independent-rates (IR) and autocorrelated-rates (AR) relaxed clock models, implemented in MCMCTree (Yang and Rannala 2006). Testing best-fit of clock models in MCMCTree (see dos Reis et al., 2018) is not possible using amino acid data, and so our assessment of divergence time estimation from the two clock models was restricted to comparisons against previous studies. The IR model generally shifted nodes towards more recent divergence times in comparison to the AR model (supplementary fig. S3, Supplementary Material online). The crown age of *Fusarium s. lat.* was estimated to fall in the late Cretaceous by both the IR (71 Ma) and AR (84 Ma) models, although the latter was closer to the estimate by O’Donnell et al. (2013) (83 Ma). The crown age of *Fusarium s. str.* estimated in the Eocene (49 Ma) by the same study was much closer to our result from the IR model (51 Ma) compared with the AR model (69 Ma, late Cretaceous). The middle Miocene crown age of the ambrosia clade in *Neocosmospora* from previous estimates by Kasson et al. (2013) (13 Ma) and O’Donnell et al. (2015) (9 Ma) were also in closer agreement with the IR model (7 Ma) compared with the AR model (25 Ma). The crown age of Xyleborini beetle hosts estimated by Jordal and Cognato (2012) (21 Ma) corresponded more closely with the IR estimate of the divergence of the ambrosia clade from non-insect mutualists (15 Ma) compared with the AR estimate (41 Ma). The dating of the diversification of various *formae speciales* in the FOSC by our IR model was also a better fit with their crop hosts having been domesticated within the last ∼10,000 years (Meyer et al. 2012).

### Gene, CSEP, and CAZyme Repertoires were Broadly Shared Across Lifestyles, But Plant Pathogens Included Copy Number Outliers

**Fig. 2.**
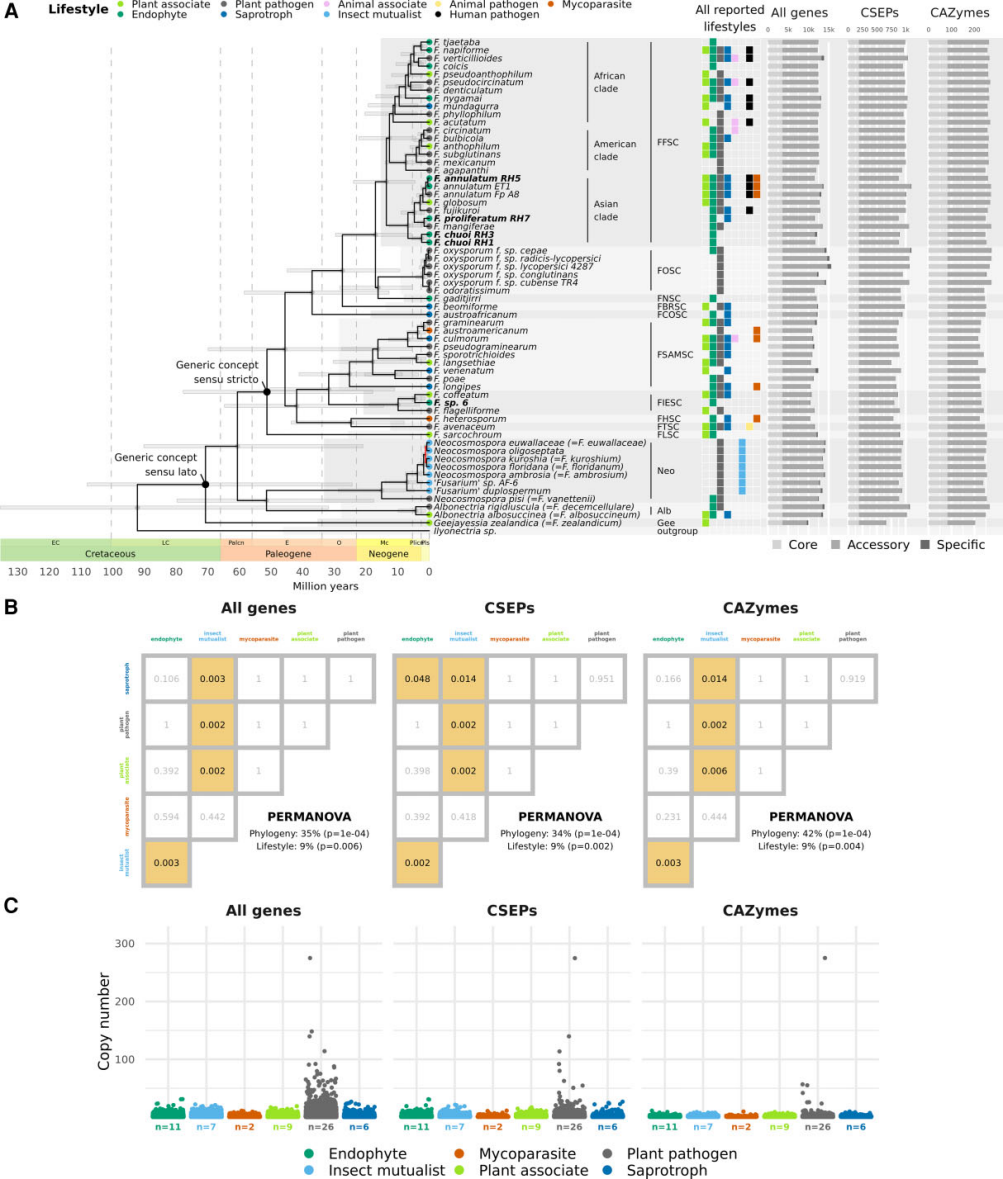
(*A*) Genome-scale phylogeny of fusarioid taxa produced by RAxML-NG from 1,060 core single-copy genes. All branches were significantly supported (≥70 FBP), except those in red. A time scale for node ages estimated by the IR relaxed clock model is shown below the phylogeny, with highest posterior density 95% confidence intervals shown as bars on nodes. For the AR model results and the exact ages and confidence intervals estimated for every node, see supplementary figure S3, Supplementary Material online. Clades corresponding to species complexes (SC) and allied genera are highlighted with alternating boxes and annotated to the right of taxon names (FFSC = *Fusarium fujikuroi* species complex, FOSC = *Fusarium oxysporum* species complex, FNSC = *Fusarium nisikadoi* species complex, FBRSC = *Fusarium burgessii* species complex, FCOSC = *Fusarium concolor* species complex, FSAMSC = *Fusarium sambucinum* species complex, FIESC = *Fusarium incarnatum-equiseti* species complex, FHSC = *Fusarium heterosporum* species complex, FTSC = *Fusarium tricinctum* species complex, FLSC = *Fusarium lateritium* species complex, Neo = *Neocosmospora*, Alb = *Albonectria*, Gee = *Geejayessia*). Lifestyles of the strains used in this study are indicated by coloured circles on tips, with other lifestyles reported from the literature summarised in the central grid (see supplementary table S1, Supplementary Material online for references). Bar graphs on the right indicate the number of genes, CSEPs and CAZymes for each taxon, with lightest to darkest colour indicating whether genes are core, accessory, or strain-specific. (*B*) Matrix of *P*-values showing whether gene, CSEP, and CAZyme content were significantly different between lifestyles according to pairwise PERMANOVA. Coloured boxes indicate significant *P* values (<0.05). Global PERMANOVA results are reported in the bottom right of plots (see also supplementary table S4, Supplementary Material online). (*C*) Scatterplot showing variation in gene copy number across all genes, CSEPs and CAZymes for different lifestyles. Points are jittered to reduce overlap. Sample size (the number of strains) is reported under *x*-axis labels.

There was no significant difference in number of genes, CSEPs or CAZymes across lifestyles (supplementary table S3, Supplementary Material online). Most genes, CSEPs and CAZymes were either core (present in all fusarioid taxa) or accessory (present in more than one taxon but not all), with very few being strain-specific, indeed strain-specific CAZymes being almost non-existent (fig. 2*A*). The number of strain-specific genes or CSEPs was not significantly different across lifestyles (supplementary fig. S4*A*, supplementary table S3, Supplementary Material online). Global pairwise permutational analysis of variance (PERMANOVA) showed that gene, CSEP, and CAZyme content were better described by phylogenetic relatedness (35–42% variance) than lifestyle (9% variance) (fig. 2*B*, supplementary table S4, Supplementary Material online). Nonetheless, pairwise PERMANOVA identified the insect mutualist lifestyle as the most genetically distinct, with insect mutualist taxa having significantly different gene, CSEP, and CAZyme repertoires compared with all other lifestyles other than mycoparasite. While most other lifestyles were genetically similar, endophytes and saprotrophs were also found to be significantly different in terms of CSEPs. In a similar pattern to the number of strain-specific genes, mean gene, CSEP, and CAZyme copy number were not found to be significantly different between lifestyles (supplementary fig. S4*B*, supplementary table S3, Supplementary Material online), but there were extreme outliers in copy number amongst plant pathogens (fig. 2*C*). The greatest copy number outlier by a considerable margin was predicted to be both a CSEP and CAZyme belonging to *F. oxysporum* f. sp. *conglutinans*, annotated as a glycosyltransferase in the GT4 family: α,α-trehalose phosphorylase (configuration-retaining) (EC 2.4.1.231).

### Almost Half of Core Single-Copy Genes were Under Positive Selection

**Fig. 3.**
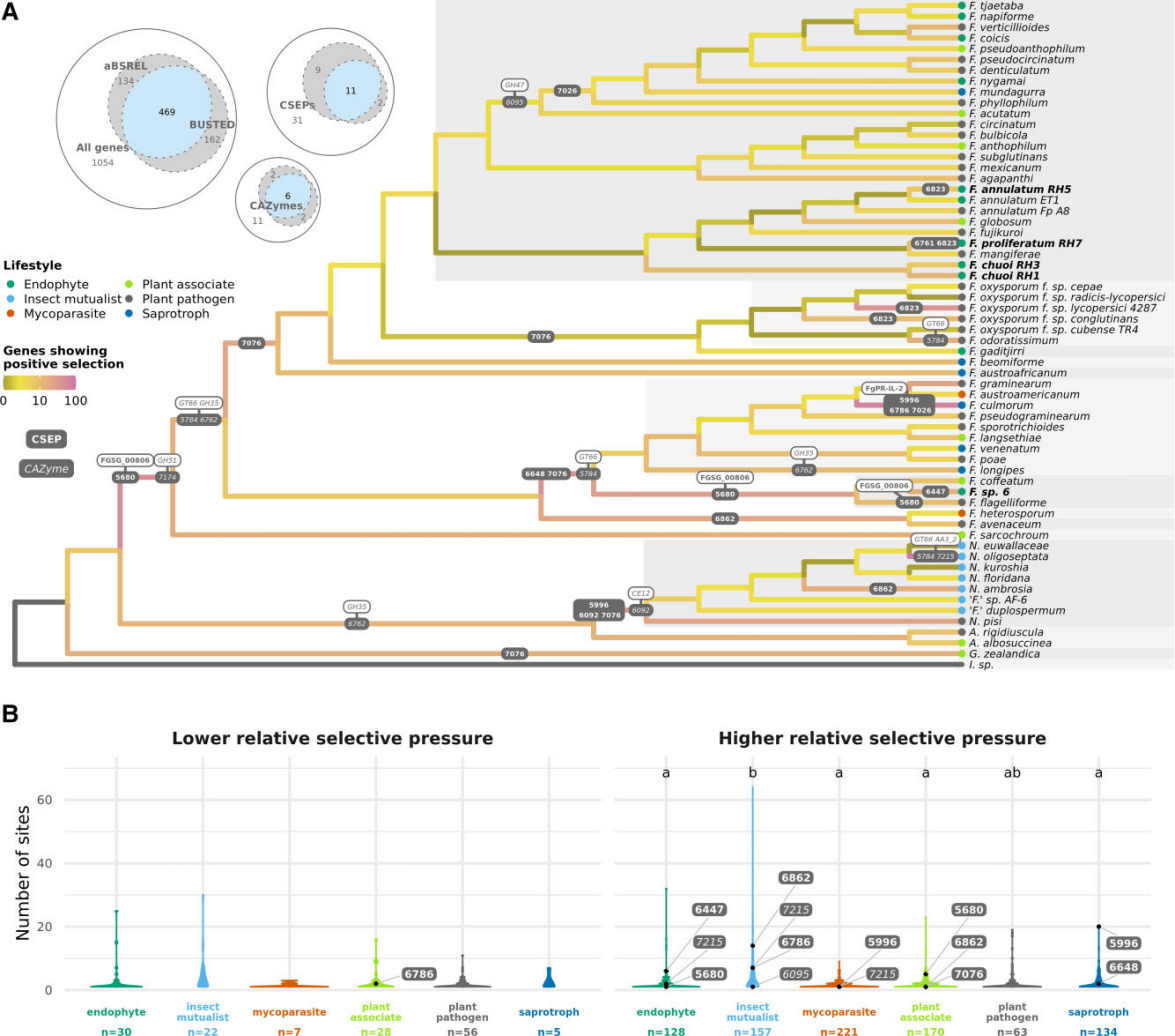
Results of dN/dS analyses on 1,054 core single-copy genes. (*A*) The Euler diagrams show the number of genes, CSEPs and CAZymes found to be under positive selection by both aBSREL and BUSTED. For the 469 cases where there was consensus between the two methods, the number of positively selected genes for each lineage according to aBSREL are shown by coloured branches on the species tree. The colour scale was pseudo log transformed for easier visualisation. For the exact number of positively selected genes on every branch, see supplementary figure S5, Supplementary Material online. Branches on which CSEPs (bold) and CAZymes (italic) were positively selected are labelled with the gene ID(s) and, where possible, more detailed functional annotation is also indicated in white labels. Lifestyles of strains are indicated by coloured circles on tips. (*B*) Violin plot showing, for genes with at least 1 site with different selective pressure, the number of sites per genes for each lifestyle with lower (left) or higher (right) selective pressure relative to all other lifestyles according to Contrast-FEL. CSEPs (bold) and CAZymes (italic) that were also reported to be positively selected by BUSTED and aBSREL are indicated by points and labelled with the gene ID. Lifestyles with significant difference of means as calculated by the Games Howell test are shown by letters to the top of the plots (see supplementary tables S3 and S5, Supplementary Material online for full statistical test results). Sample size (the number of genes) is reported under *x*-axis labels.

While gene, CSEP, and CAZyme repertoires may have been broadly shared, we were interested in whether genes were evolving in a lifestyle-directed manner. Of the 1,054 core single-copy genes used in the selection analyses, 469 (44%) were found to be under episodic positive selection by both BUSTED (Murrell et al. 2015) and aBSREL (Smith et al. 2015) (fig. 3*A*). This included 11 of 31 (35%) core CSEPs and 6 of 11 (55%) core CAZymes. The branch at the root of the more conservative generic concept, *Fusarium s. str.*, was a particular “hotspot” of positive selection, with 52 core single-copy genes positively selected according to BUSTED and aBSREL (supplementary fig. S5, Supplementary Material online). A few external branches also had a notably high number of positively selected core genes: insect mutualist *N. oligoseptata*; saprotrophic *F. culmorum* in the *F. sambucinum* species complex (FSAMSC); and plant pathogenic *F. oxysporum* f. sp. *lycopersici* in the FOSC. There was no significant difference in the number of positively selected genes on external branches between lifestyles according to analysis of variance (ANOVA, *P* = 0.7; supplementary table S3, Supplementary Material online).

Although a minority of all CSEPs (11%) could be assigned known gene names using the PHI-base database (Urban et al. 2020), two core CSEPs with signatures of selection could be classified as known genes: 5680 as FGSG_00806 and 6786 as FgPR-IL-2 (fig. 3*A*). Based on PHI-base records of gene knockouts in *F. graminearum* inoculated on wheat, both FGSG_00806 and FGPR-IL-2 had the mutant phenotype of unaffected pathogenicity (supplementary fig. S6, Supplementary Material online). Of the six core CAZymes which had undergone positive selection, four are known to act on plant cell wall substrates (supplementary fig. S7, Supplementary Material online): glycoside hydrolase GH35 (β-galactosidase) on hemicellulose and pectin and GH51 (non-reducing end α-l-arabinofuranosidase) on cellulose, hemicellulose and pectin; carbohydrate esterase CE12 (rhamnogalacturonan acetylesterase) on pectin; and an enzyme of auxiliary activities AA3_2 (5′-oxoaverantin cyclase) on lignin.

Most CSEPs and CAZymes reported as positively selected by both BUSTED and aBSREL were also found to contain sites with a higher relative selective pressure in certain lifestyles by Contrast-FEL (Kosakovsky Pond et al. 2021) (fig. 3*B*). In most cases only one site per gene was found to have a difference in relative selective pressure. The insect mutualist lifestyle had significantly more sites per gene under higher selective pressure compared with most other lifestyles (fig. 3*B*). We should emphasise that Contrast-FEL does not inform whether positive or negative selection is occurring on a branch set, only that there is a relative increase or decrease in dN/dS, and thus higher or lower selective pressure, compared with other branches. We reasoned that if a CSEP or CAZyme with higher relative selective pressure for a lifestyle was also found to be positively selected on an external lineage of that lifestyle, then it could suggest that the selective pressure is imposed by lifestyle. This was the case for 4 of the 9 core CSEPs and 1 of the 3 core CAZymes identified as positively selected on external lineages: CSEPs 6447 (*F.* sp. 6, endophyte); 5996 (*F. culmorum*, saprotroph); 6862 (*N. ambrosia,* insect mutualist); and 7076 (*Geejayessia zealandica*, plant associate); and CAZyme 7215 of lignin degrading subfamily AA3_2 (*N. oligoseptata*, insect mutualist).

### Codon Optimization was Higher in *Fusarium s. str*.

**Fig. 4.**
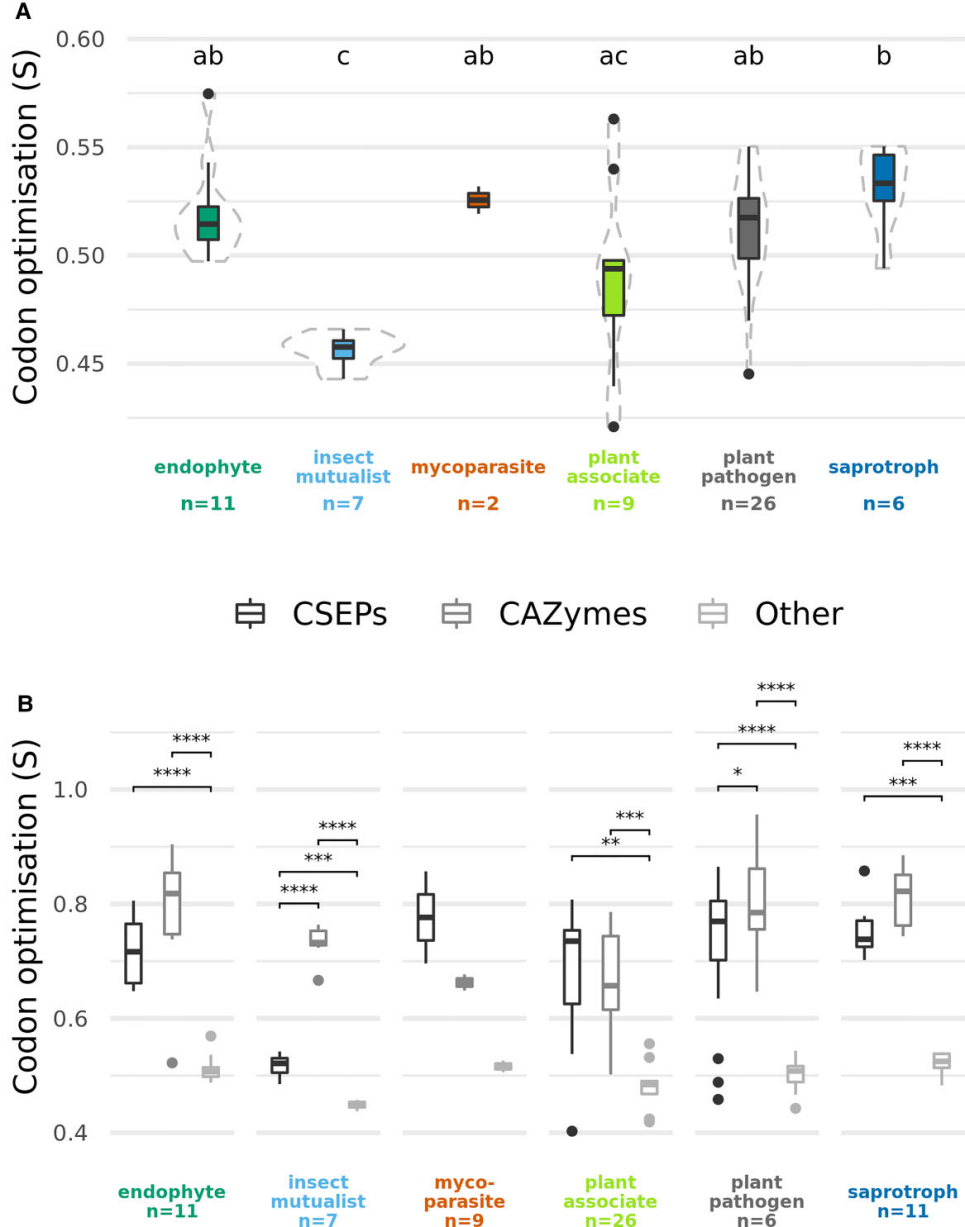
Boxplots showing codon optimisation (S) of core single-copy genes across lifestyles. Sample size (the number of strains) is reported under *x*-axis labels. (*A*) Difference in overall S values between lifestyles, with significant difference of means as calculated by TukeyHSD shown by letters at the top of the plot (see supplementary tables S4 and S6, Supplementary Material online for full statistical test results). (*B*) Difference in S values between CSEPs, CAZymes, and other genes for each lifestyle, with significant difference of means between the gene type as calculated by the Games Howell test shown by bars across significantly different categories (*P < 0.05, **P < 0.01, ***P < 0.001, ****P < 0.0001; see supplementary tables S3 and S5, Supplementary Material online for full statistical test results).

As dN/dS methods are biased by the erroneous assumption that all synonymous substitutions are neutral (Hershberg and Petrov 2008; Rahman et al. 2021), we also explored whether translational selection (i.e., bias towards certain codons in more highly expressed genes) may be acting on synonymous substitutions by assessing the extent of codon optimisation (S) across fusarioid taxa (dos Reis et al. 2004). Codon optimisation of 1,054 core single-copy genes was generally high for all taxa (between 0.4 and 0.6, on a scale from −1 to 1), but it was significantly lower in insect mutualists compared with endophytes, plant pathogens and saprotrophs (fig. 4*A*, supplementary table S5, Supplementary Material online). S values were found to be significantly higher in CSEPs and CAZymes than other core single-copy genes for all lifestyles (excluding mycoparasite, which could not be tested due to small sample size); furthermore, codon optimisation of CAZymes was also significantly higher than CSEPs for insect mutualists and plant pathogens (fig. 4*B*, supplementary table S5, Supplementary Material online). CSEPs and CAZymes also encompassed greater extremes of codon optimisation than other core genes.

As high levels of codon optimisation has been linked to host generalism in fungi (Badet et al. 2017) and codon usage bias to wide habitat range in prokaryotes (Botzman and Margalit 2011), we speculated that higher codon optimiszation may be associated with lifestyle generalism, that is, taxa being capable of exhibiting multiple lifestyles. When no data correction was performed, there was a medium strength positive correlation between the number of reported lifestyles or “lifestyle range” and S values (Pearson’s *r* = 0.3, *P* = 0.01), but the statistical significance of this correlation did not hold when accounting for phylogenetic relationships with phylogenetic generalised least squares (PGLS) analysis (*P* = 0.06) (supplementary fig. S8, Supplementary Material online).

**Fig. 5.**
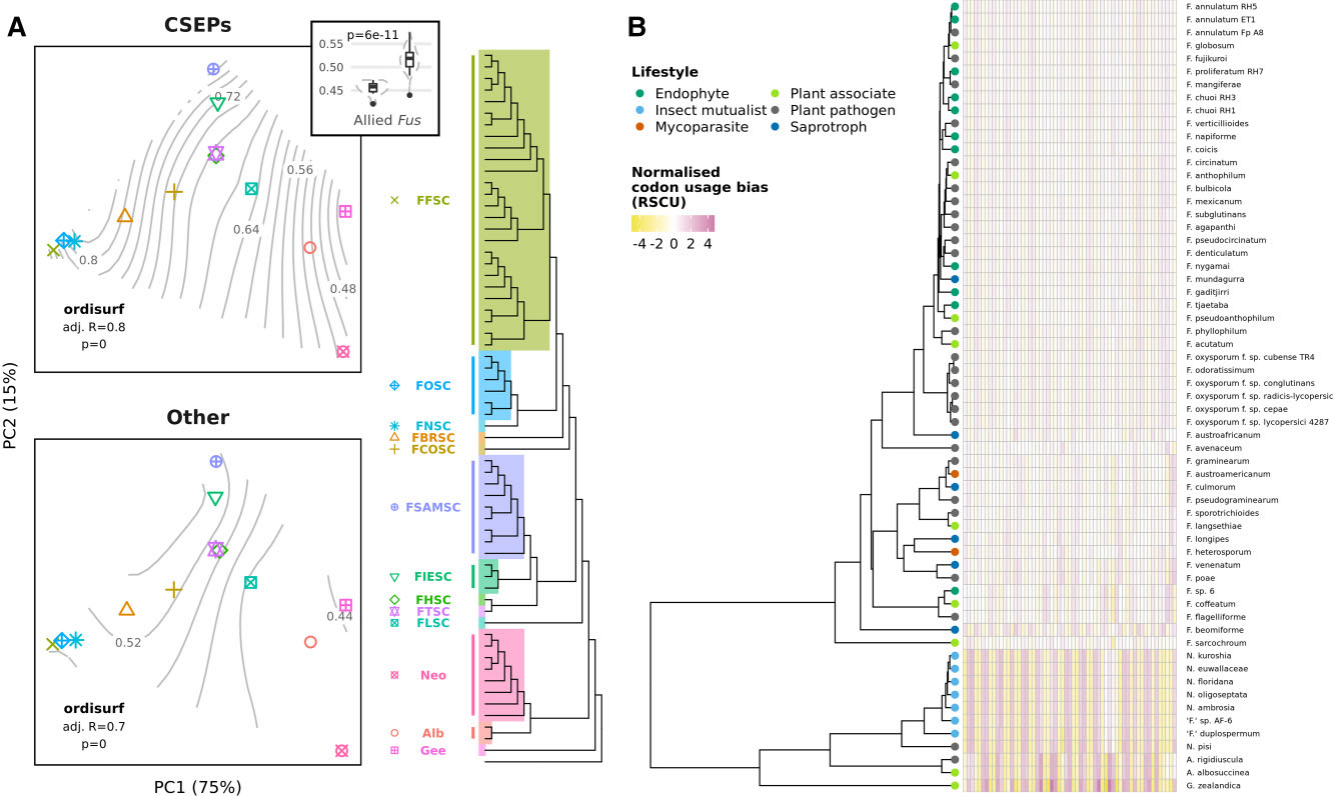
(*A*) PCA of phylogenetic distances between taxa, with points representing centroids for species complexes/allied genera, differentiated by shape and colour, as indicated by the tree legend. The percentage of variance explained by each principal component is shown on axis labels. Contours indicate the fit of codon optimisation (S values), of both core CSEPs and other core genes, to the ordination; the fit of CAZyme codon optimisation is not shown as it was not significant (*P* = 0.2). The inset boxplot shows the significant difference (*t*-test, *P* = 6e−11) in overall S values between *Fusarium* and allied genera. (*B*) Hierarchical clustering of taxa according to normalised codon usage bias (RSCU). Heatmap columns represent codons (excluding Trp, Met, and stop codons) with cells coloured by normalised RSCU, where positive values represent higher than expected codon usage and negative values represent lower than expected codon usage.

There was significantly higher codon optimisation in species complexes belonging to *Fusarium s. str.* compared with allied genera (*t*-test, *P* = 6e−11; fig. 5*A* inset). Codon optimisation for CSEPs was shown to be strongly correlated with phylogeny as shown by the fit of S values to a principal component analysis (PCA) of phylogenetic distances (fig. 5*A*). This was not the case for CAZymes, however, for which the fit of codon optimisation to the PCA was not significant (*P* = 0.2). Hierarchical clustering of taxa by normalised relative synonymous codon usage (RSCU) was also reasonably concordant with the species tree, with a Robinson–Foulds distance of 0.4 (*P* = 0; fig. 5*B*), indicating that codon usage bias, for CSEPs if not CAZymes, is likely to be influenced by shared ancestry more than lifestyle.

## Discussion

In this study, we inferred a phylogeny of *Fusarium* and allied genera using the greatest number of loci to date, with almost all branches significantly supported (fig. 2*A*). This adds to numerous recent efforts to produce high quality fungal phylogenies from genome-scale data (e.g., Spatafora et al. 2016; Steenwyk et al. 2019; Varga et al. 2019; Li et al. 2021). Trimming method and inclusion/exclusion of multi-copy genes had some impact on species tree topology (fig. 1, supplementary fig. S2, Supplementary Material online), but the *Fusarium s. str.* backbone was consistent across all approaches and in general agreement with the most recently published phylogeny of the group (Crous, Osieck, et al. 2021). Discordance was concentrated in the ambrosia clade in *Neocosmospora*, perhaps due to the occurrence of interspecific hybridization in this lineage (Kasson et al. 2013) or horizontal gene transfer via the exchange of strains by beetles (Hulcr and Cognato 2010). The objectives of this study were not concerned with the taxonomic debates surrounding the *Fusarium* generic concept, but our results did show that the divergence between *Fusarium s. str.* and other fusarioid taxa was associated with positive selection on a considerable number of core genes (fig. 3*A*); an upwards shift in translational selection (fig. 5*A*); and distinct patterns in codon usage bias (fig. 5*B*). While these results obviously do not directly contribute to characterisation of the taxa involved, they might be seen as a symptom of a “larger and more abrupt” divergence than that between species within the same genus (Booth 1978), contrary to *Fusarium s. lat.* (O’Donnell et al. 2020; Geiser et al. 2021).

We generally found the IR molecular clock model to produce dating estimates that were more concordant with estimates from other studies assessing divergence times of fusarioid fungi (e.g., Kasson et al., 2013; O’Donnell et al., 2013, 2015), which was largely to be expected considering that these studies also used IR models (but different secondary calibrations). The IR model estimated the divergence of obligate insect mutualists to correspond more closely to the crown age of their insect hosts, as estimated with insect fossil calibrations (Jordal and Cognato 2012). By contrast, the AR model appeared to produce less congruent ages for recently diverged lineages, such as the highly specialised FOSC strains diverging before their host plants are likely to have existed. AR models have generally been thought appropriate for plants and animals considering the correlation between substitution rate and life-history traits (Lartillot et al. 2016), and it has furthermore been suggested that AR is the norm across all kingdoms of life (Tao et al. 2019). On the other hand, Taylor and Berbee (2006) found no lineage-specific correlation of substitution rates across the kingdom Fungi. Similarly, Linder et al. (2011) did not find strong evidence for rate autocorrelation across plant and simian datasets, instead finding the IR model to have more explanatory power. The AR model is not immune to bias (Lartillot and Delsuc 2012), and has been shown to produce older estimates for simulated datasets across dating tools, including MCMCTree (Miura et al. 2020).The presence of short-term rate fluctuations in mammals suggest that mixed relaxed clock models accounting for both autocorrelation and jumps in rate variation are needed (Ho 2009; Lartillot et al. 2016).

Sources of error in divergence time estimation are manifold, as evidenced by the large confidence intervals in our analysis (supplementary fig. S3, Supplementary Material online). Beyond the difficulty surrounding choice and implementation of molecular clock models, a major source of error is the use of secondary calibrations—a necessity due to the general lack of fungal fossil data (Beimforde et al. 2014)—which can impact the precision and accuracy of divergence time estimates (Shaul and Graur 2002; Graur and Martin 2004; Sauquet et al. 2012; Schenk 2016). For this reason, we incorporated the error from node ages estimated using primary fossil calibrations (Lutzoni et al. 2018) using confidence intervals to provide upper and lower bounds, as recommended when using secondary calibrations (Graur and Martin 2004; Forest 2009; Hipsley and Müller 2014). An alternative approach is to expand taxon sampling until fossil data can be incorporated, although secondary calibrations have been shown to produce divergence time estimates with similar accuracy to those from distant primary calibrations, albeit with lower precision (Powell et al. 2020). Our motivation for divergence time estimation was not to test specific time-dependent hypotheses, but rather to calibrate branch lengths for more realistic measures of phylogenetic distance in subsequent comparative analyses. As with any divergence time analysis, major uncertainties are still associated with the divergence times of fusarioid fungi.

All taxa had a similar number of genes, CSEPs and CAZymes, very few of which were strain-specific (fig. 2*A*). It has previously been suggested that the number of species-specific secreted proteins (and by extension, we assume, effectors) is generally higher in fungal lifestyles which associate with plants without killing or decaying them, such as mutualistic symbionts and biotrophic pathogens, compared with saprotrophs and necrotrophic pathogens (Kim et al. 2016), the reasoning being that the former have to negotiate the plant–fungal interaction for an extended period. In the genus *Colletotrichum*, however, a reduction in the number of species-specific CSEPs was observed alongside the transition from phytopathogenicity to beneficial endophytism (Hacquard et al. 2016), showing that CSEPs and their impact on the plant–fungal interaction can be highly lineage-specific. We saw no significant difference in the number of strain-specific CSEPs (or genes) between any lifestyles (supplementary fig. S4, Supplementary Material online). This, combined with the fact that plant pathogens are often also reported as endophytes and vice versa (fig. 2*A*), and that plant pathogen and endophyte strains were not significantly different in terms of gene and CSEP content (fig. 2*B*), suggests that fusarioid taxa have a shared genetic capacity for phytopathogenicity and/or endophytism. Having a high proportion of species-specific CSEPs has also been associated with the connected factor of host specialisation (Spanu et al. 2010), which, considering we report very low numbers of strain-specific genes, may also explain the status of many *Fusarium* taxa as host generalists. Our results were also similar to those comparing pathogenic and non-pathogenic taxa in another genus of broad generalists, *Aspergillus* (Mead et al. 2021).

We did not identify common genetic signatures for the endophytic lifestyle in terms of gene, CSEP or CAZyme content, reinforcing the current understanding that there is no universal “toolkit” associated with the endophytic lifestyle (Hacquard et al. 2016; Knapp et al. 2018). This contrasts with other well-defined lifestyles such as that of mycorrhizal fungi, for which specific genetic features have been associated with lifestyle in both ascomycetes and basidiomycetes (Martin et al. 2010; Delaux et al. 2013; Kohler et al. 2015; Peter et al. 2016; Miyauchi et al. 2020; Rich et al. 2021). One observed hallmark of the transition to mycorrhizal symbiosis is the loss of genes encoding PCWDEs (Kohler et al. 2015; Peter et al. 2016; Miyauchi et al. 2020), but, as we found here, these are retained in various endophytic taxa (Zuccaro et al. 2014; Lahrmann et al. 2015; Hacquard et al. 2016; Franco et al. 2022; Mesny et al. 2021). As PCWDEs have often been treated predominantly as features of saprotrophy, this has fed into the hypothesis that many endophytes are latent saprotrophs, but in a broad comparison of CAZymes across the Dikarya, Zhao et al. (2013) demonstrated that plant pathogens have on average more CAZymes belonging to typical PCDWE families than saprotrophs. As there was no significant difference in total number or repertoire of CAZymes between plant pathogens, endophytes and saprotrophs, it indicates that fusarioid fungi retain the same machinery for plant cell wall degradation and/or remodelling, regardless of lifestyle. We did, however, find a significant difference in CSEP content between saprotrophs and endophytes (fig. 2*B*), which could suggest that fusarioid endophytes are more likely to be latent pathogens than saprotrophs.

The major exception to the apparent lifestyle flexibility among fusarioid fungi is the insect mutualist lifestyle, which formed a monophyletic group (the ambrosia clade) in *Neocosmospora* (fig. 2*A*). The insect mutualist lifestyle was also the most distinct in terms of gene and CSEP content, being significantly different from all other lifestyles apart from the mycoparasitic lifestyle (fig. 2*B*), but the very small sample size for the latter will have impacted the test’s power in that case (Alekseyenko 2016). The transition to symbiotic mutualism in *Neocosmospora* was not associated with a reduction in total number of genes, CSEPs or CAZymes, in agreement with results from other ectosymbiotic insect mutualists (Biedermann and Vega 2020). As the representative strains used in this study are all known to cause disease on the trees they colonise with their beetle partner (Freeman et al. 2013; O’Donnell et al. 2016; Na et al. 2018; Aoki et al. 2019), it follows that they would have retained many of the genetic mechanisms from their (presumably) plant associated ancestors. Some strains have been found to cause disease *in vitro* in the absence of their beetle partners (e.g., Eskalen et al., 2012; Na et al., 2018), however, to our knowledge, fusarioid ambrosia fungi have never been reported as free-living in the wild.

Although we did not identify significant differences in the genetic repertoires between fusarioid endophytes and plant pathogens, we did find some evidence that copy number variation—genes or regions that are either duplicated or deleted in reference to other taxa—may be contributing to lifestyle. There was no significant difference in mean gene copy number between lifestyles, but plant pathogens included extreme outliers in gene copy number compared with other lifestyles (fig. 2*C*). Extensive gene duplication has been suggested as a key strategy for pathogenicity in basidiomycete rusts (Pendleton et al. 2014), and copy number of the pectin degrading CAZyme subfamily PL1_7 across 41 root-colonising fungi was shown to correlate with pathogenicity in *Arabidopsis* (Mesny et al. 2021). Gene duplication is regarded as the primary resource for the evolution of functional novelties, and the persistence of gene duplicates is indicative of neofunctionalisation and/or subfunctionalisation, as a functionally redundant gene copy will be rapidly lost due to the absence of selective pressure to retain it (Lynch and Conery 2000; He and Zhang 2005). The most common functional innovations of gene copies in fungi are regulatory changes (Wapinski et al. 2007). Indeed, copy number variation is known to be correlated with differential gene expression (Stranger et al. 2007; Steenwyk and Rokas 2018; Shao et al. 2019), and has been shown to contribute to phenotypic or pathological differences in fungi (Steenwyk et al. 2016; Zhao and Gibbons 2018).

This aligns with mounting evidence that a major factor impacting lifestyle of closely related phytopathogens and endophytes is not gene repertoire itself, but expression profiles. Returning to *Colletotrichum*, Hacquard et al. (2016) found that a pathogenic taxon had a different pattern of gene expression during host colonisation, including upregulation of CSEPs, compared with a closely related and genetically similar beneficial endophyte. The authors noted that this also makes the beneficial endophyte genetically capable of reverting to pathogenicity (and, presumably, the closely related pathogens capable of inhabiting plants as endophytes). The aforementioned CAZyme subfamily PL1_7, which we found between 2 and 4 copies of in all fusarioid taxa (supplementary fig. S7, Supplementary Material online), was also more highly expressed in the pathogenic *Colletotrichum* taxon. The importance of expression has already been seen in *Fusarium*, where expression of secondary metabolites differed between endophytic and pathogenic strains of the same species, *F. annulatum* (as *F. proliferatum,* FFSC), despite generally sharing secondary metabolite gene clusters (Niehaus et al. 2016). Generating *in planta* expression profiles for both pathogenic and non-pathogenic strains across the group could reveal whether there is convergence in expression patterns for certain lifestyles.

Regulation of certain genes located on accessory chromosomes has also been seen to direct plant infection phenotypes in an endophytic versus pathogenic FOSC strain (Guo et al. 2021). Accessory chromosomes—chromosomes that are not essential for survival, but potentially confer functional advantages (Bertazzoni et al. 2018)—are likely another important factor impacting lifestyle in *Fusarium*. The first acc. chromosomes in fungi were discovered in the fusarioid species *Neocosmospora haematococca* (as *Nectria haematococca*) (Coleman et al. 2009), with further reports in at least nine other fusarioid strains (Bertazzoni et al. 2018). They have mostly been studied in the FOSC, in which horizontal transfer of acc. chromosomes can confer pathogenicity (Ma et al. 2010; Li et al. 2020). Not only are acc. chromosomes deemed to be a key innovation for rapid adaptation by plant pathogens (Croll and McDonald 2012) they have also been implicated in adaptation of FOSC strains to human pathogenicity (Zhang, Yang, et al. 2020). Exploring the extent of acc. chromosomes broadly across fusarioid fungi, as well as phenomena impacting genomic architecture such as transposable elements (Muszewska et al. 2019), may shed light on the mechanisms underlying lifestyle flexibility in the group (Ma et al. 2013).

As effectors are highly diverged and often lineage-specific, if not strain-specific, only a small proportion of the CSEPs predicted here could be matched to experimentally verified genes from PHI-base. Of these, the majority were genes known to impact virulence to some degree or not at all in the hosts they have been tested on (supplementary fig. S6, Supplementary Material online), although the knockout mutant phenotype for a certain gene will not necessarily be the same for different fungal strains or on different hosts. PHI-base is also explicitly dedicated to pathogen-host genes, and similar high quality, curated resources are needed for genes involved in non-pathogenic fungal–host interactions. Nonetheless, our results give us a broad perspective on CSEP distributions across fusarioid fungi. Some CSEPs exhibited phylogenetic patterns (such as lower copy number in *Fusarium s. lat.* compared with *Fusarium s. str.* for MoCDIP4, which was first discovered in *Magnaporthe oryzae* (Chen et al. 2013) and since reported in *F. oxysporum* f. sp. *pisi* (Achari et al. 2021)), but most had scattered distributions across the group (supplementary fig. S6, Supplementary Material online), which may be the result of frequent horizontal gene transfer (e.g., van Dam and Rep 2017; Peck et al. 2021).

A slightly lower proportion of core CSEPS were found to be positively selected than non-CSEPs according to dN/dS calculations (fig. 3*A*). This may be seen as surprising, as effectors that promote virulence are assumed to be under strong selective pressure during the evolutionary arms race between fungus and host (De Jonge et al. 2011; Lo Presti et al. 2015). For instance, CSEPs have been found to more frequently be under positive selection compared with non-CSEPs in phytopathogenic *Microbotryum* species (Beckerson et al. 2019). High rates of selection on CSEPs are not only a hallmark of pathogenicity, however, as these have also been observed for obligate, host-specific *Epichloë* endophytes (Schirrmann et al. 2018); the arbuscular mycorrhizal fungus *Rhizophagus irregularis* (Schmitz et al. 2019); and the saprotroph *Verticillium tricorpus* (Seidl et al. 2015), emphasizing the broader roles played by effectors in host–fungal interactions. Our results could be explained by the fact that we focused on core genes, and so the CSEPs in questions are presumably contributing to integral host–fungal interactions that would be under similar selective pressure as other core functions, rather than specialised CSEPs more likely to be under strong selective pressure from the host. We should also note that detection of positive selection with dN/dS methods is biased against shorter genes (Derbyshire et al. 2021), which CSEPs by definition are, and so this may have impacted our results.

We identified five cases where positive selection of core CSEPs and CAZymes may be connected to lifestyle by comparing aBSREL analysis of positive selection on external branches to Contrast-FEL analysis of relative selection pressures between lifestyles. Interestingly, there were no core CSEPs with higher selective pressure in plant pathogens relative to other lifestyles, which could be interpreted as evidence that the ancestral state of the group is phytopathogenic rather than endophytic, but the unbalanced sample sizes for the different lifestyles will have influenced the Contrast-FEL results. Once again, the insect mutualist lifestyle was shown to be distinct, with a greater number of sites per gene undergoing higher selective pressure relative to other lifestyles (fig. 3*B*). This may be associated with the fact that these ambrosia taxa have evolved via insect farming, in what could be interpreted as some level of “artificial selection” (Mueller et al. 2005). We were only able to tentatively link the positive selection of one core CAZyme to lifestyle: 5′-oxoaverantin cyclase in the AA3_2 subfamily, which was positively selected for in the insect mutualist *N. oligoseptata* (fig. 3*A*). Other members of the same subfamily are implicated in lignin degradation (Levasseur et al. 2013; Miyauchi et al. 2017), but 5′-oxoaverantin cyclase was first identified as an intermediate in aflatoxin biosynthesis in *Aspergillus parasiticus* (Sakuno et al. 2003). Another insect-fungus mutualism between the navel orangeworm and *A. flavus* has shown that aflatoxin tolerance is a key adaptation of the insect to its fungal diet (Niu et al. 2009; Ampt et al. 2016), and as fusarioid fungi are known to produce an array of mycotoxins (Desjardins and Proctor 2007), it would be interesting to determine whether there is a similar dynamic in the evolution of the ambrosia mutualism.

Conventional dN/dS methods to detect selection such as aBSREL and BUSTED make the assumption that synonymous substitutions are always selectively neutral, but we now know that selection does occur on synonymous mutations (Ohta 1996; Chen et al. 2004; Hershberg and Petrov 2008). Subsequently dN/dS methods have been shown to overestimate the frequency of positive selection and underestimate the strength of negative selection in bacteria, even when selection on synonymous sites is weak (Rahman et al. 2021). Furthermore, using dN/dS > 1 as a signifier of positive selection has been declared arbitrary (Tamuri and dos Reis 2021). As flexible dN/dS methods accounting for selection on synonymous substitutions have yet to be integrated into the widely used tools for detecting positive selection, this remains a caveat of our dN/dS analyses. Additionally, even a low incidence of sequence inaccuracies can results in false-positive signals of selection (Mallick et al. 2009), so ideally candidate genes should be resequenced to detect errors and confirm whether sites are truly under selection. A further limitation of the selection analyses is that they were restricted to core genes due to the requirement of a robust species tree to estimate dN/dS across lineages, which necessarily excludes a large proportion of the gene content (Derbyshire et al. 2021). Further exploration of selection dynamics in the extensive accessory content would undoubtedly shed more light on the evolution of the group.

When exploring the issue of selection on synonymous substitutions, we showed that codon optimisation of the core single-copy genes—that is, the extent of translational selection on codon usage—was higher in CSEPs and CAZymes than other genes (fig. 4*B*), as was previously found in the *F. oxysporum* f. sp. *cepae* pangenome (Armitage et al. 2018). Insect mutualists had a much larger difference in codon optimisation between CSEPs and CAZymes (fig. 4*B*). One possible explanation for this result is that these taxa may have less translational selective pressure on CSEPs that are required for plant invasion—being farmed by insects which excavate and weaken the plant hosts—but retain higher translational selective pressure on CAZymes that are required for assimilation of nutrients, which ultimately maintains the insect-fungus mutualism. Following this broad perspective on codon optimisation, further functional annotation could allow the use of a “reverse ecology framework” to explore whether genes with the highest codon optimisation correspond with lifestyle (LaBella et al. 2021).

We also found that correlation between lifestyle range and codon optimisation was not significant after correcting for phylogenetic relationships (supplementary fig. S8, Supplementary Material online), contrary to expectation from previous studies (Botzman and Margalit 2011; Badet et al. 2017). Our approach to assess lifestyle range was limited by the availability of published reports of fusarioid taxa, and so we will undoubtedly have underestimated the number of lifestyles exhibited by some species. Furthermore, fusarioid species are often hard to distinguish, and lifestyle reports may therefore be misattributed. To mitigate against this issue, we only included studies that used appropriate genetic markers to distinguish taxa—not, for instance, solely using ITS (Geiser et al. 2004)—and crosschecked phylogenetic analyses for misclassifications. Despite this, we may have inadvertently included lifestyle reports for species that were incorrectly classified in the original study. A comprehensive meta-analysis is needed to better understand the extent of lifestyle and host range for fusarioid taxa.

A major caveat of our comparative analyses is that we were forced to attribute a single lifestyle to the strains being used, despite the current understanding, which our own results support, that these lifestyles are not necessarily mutually exclusive (Selosse et al. 2018). Furthermore, treating lifestyles as categorical traits does not accurately reflect the range of outcomes we know can exist within even one lifestyle, such as different pathogenic strains within the same species varying in “aggressiveness” (e.g., Holtz et al. 2011; Chen et al. 2014; Šišić et al. 2018). These both remain central issues with current approaches to fungal lifestyle comparison at large (e.g., Knapp et al. 2018; Miyauchi et al. 2020; Mesny et al. 2021; Franco et al. 2022). New methods that can effectively incorporate multiple lifestyle hypotheses, or treat lifestyles as points on a continuous spectrum, are sorely needed to encapsulate the nuance of these highly context-dependent interactions.

## Conclusions

We found an apparent shared genetic capacity for phytopathogenicity and endophytism in *Fusarium*, which suggests that, while strains may be reported as plant pathogens or endophytes, their lifestyle is potentially transient. Were fusarioid taxa to make the transition to obligate, mutualistic endophytism, we might expect to see genetic hallmarks more akin to those seen in the transition to obligate symbiosis in mycorrhizal lifestyles (e.g., Delaux et al., 2013). Despite multiple reports of certain endophytic *Fusarium* strains being beneficial to certain plant hosts (e.g., Kavroulakis et al., 2007; Mendoza and Sikora, 2009; Bilal et al., 2018), large uncertainties remain as to the stability of these interactions. Our results depict fusarioid fungi as prolific generalists and highlight the difficulty in predicting pathogenic potential in the group. Considering the importance of plant immune response, biotic and abiotic conditions to the plant–fungal interaction, such endophytes may not be the “silver bullet” for biocontrol that they are sometimes touted to be.

## Materials and Methods

### Genome Sequencing, Assembly, and Structural Annotation

We selected five endophytic *Fusarium* strains for whole-genome seuence which were representatives of species hypotheses that had previously been isolated and clustered into 99% similarity operational taxonomic units by Hill et al. (2021), with taxonomic identification confirmed where possible via morphological assessment by the Westerdijk Institute (supplementary table S2, Supplementary Material online). For DNA extractions, a fragment of mycelium from axenic cultures was transferred to 500 ml of 2% malt extract nutrient broth using a sterile needle and grown at 25 °C in ambient light conditions on an orbital shaker at 120 rpm for ∼1 week. Mycelia were collected via vacuum filtration and frozen at −80 °C before being pulverised with two sterile stainless-steel beads in a 2 ml Eppendorf using a Mixer Mill MM 400 (Retsch, Germany).

DNA was extracted using the DNeasy Plant Mini Kit (Qiagen, CA, USA) according to the manufacturer’s protocol and eluted in 70 μl of TE buffer. Sufficient DNA concentration (more than 20 ng/µl) was confirmed with a Quantus^™^ Fluorometer (Promega, WI, USA) and purity (260/280 absorbance ratio of approximately 1.8) confirmed with a NanoDrop spectrophotometer (Thermo Fisher Scientific, MA, USA). DNA extractions were sent to Macrogen (Macrogen Inc., South Korea) for library preparation and sequencing: library preparation was performed using the TruSeq DNA PCR-free Sample Preparation Kit with a 550 bp insert size and 151 bp paired-end reads were sequenced using the NovaSeq 6000 platform (Illumina, San Diego, CA, USA).

Our assembly and annotation methodology are described in full detail in Supplementary material. In brief, reads were assembled with ABySS v2.0.2 (Simpson et al. 2009) and polished with Pilon v1.23 (Walker et al. 2014). Contiguity was assessed using QUAST v5.0.2 (Gurevich et al. 2013); completeness as measured by gene sets was assessed using BUSCO v3.0.1 (Simão et al. 2015); and absence of contamination was checked using BlobTools v1.1 (Laetsch and Blaxter 2017). Assemblies were annotated following the MAKER pipeline (Cantarel et al. 2008). See supplementary table S6, Supplementary Material online for a summary of assembly quality statistics.

### Phylogenomic Analyses

Predicted genes from 57 additional publicly available strains of *Fusarium* and allied genera were downloaded from NCBI (supplementary table S1, Supplementary Material online) and orthogroups (referred to here as genes) were inferred from amino acid sequences of the total 62 strains using OrthoFinder v2.4.0 (Emms and Kelly 2019). We aligned 1,060 core (i.e., shared between all fusarioid taxa including the outgroup) single-copy genes using MAFFT v7.310 with default settings (Katoh and Standley 2013) and removed ambiguously aligned regions using both BMGE v1.12 (Criscuolo and Gribaldo 2010) and trimAl v1.4.rev15 with the gappyout option (Capella-Gutiérrez et al. 2009) to compare the impact of trimming tools on the resulting species trees.

For a concatenation-based approach, core single-copy gene alignments were concatenated with AMAS v0.98 (Borowiec 2016). We compared two tools for maximum likelihood (ML) species tree estimation: IQ-TREE v2.1.2 (Minh et al. 2020) and RAxML-NG v1.0.1 (Kozlov et al. 2019), with the concatenated alignment partitioned by gene in both cases. For IQ-TREE, the best-fit amino acid substitution model for each partitioned gene was selected by the inbuilt tool ModelFinder (Kalyaanamoorthy et al. 2017) using Bayesian information criterion (BIC) values, and branch support was computed via 1,000 ultrafast bootstrap replicates (UFBoot) (Hoang et al. 2018). For RAxML-NG, ModelTest-NG v0.1.6 (Darriba et al. 2020) was used to select substitution models for each gene using Akaike information criterion (AIC) values, and branch support was computed via 100 Felsenstein’s bootstrap replicates (FBPs). FBP convergence was confirmed with the --bsconverge option using the default 3% cutoff for weighted Robinson–Foulds distances (Pattengale et al. 2010).

For a coalescent-based approach, ML gene trees were inferred from each core single-copy gene alignment with RAxML-NG using the best-fit model selected by ModelTest-NG during the concatenated analysis. The resulting ML gene trees were used for coalescent-based species tree reconstruction using ASTRAL-III v5.7.3 (Zhang, Yohe, et al. 2018) with local posterior probability (LPP) branch support estimation (Sayyari and Mirarab 2016). ASTRAL-Pro v1.2 (Zhang, Scornavacca, et al. 2020) was additionally run with LPP support estimation on the 20,343 gene trees produced by OrthoFinder, which represented both single- and multi-copy “total” genes. OrthoFinder itself also produces a coalescent-based species tree topology by default using STAG (Emms and Kelly 2018), which used 3,449 core single- and multi-copy genes. All species tree topologies were compared by computing the normalised Robinson–Foulds metric using the RF.dist function from the phangorn v2.7.0 package (Schliep et al. 2017) in R v4.0.4 (R Core Team 2020).

### Molecular Clock Analyses

The species tree topology inferred by RAxML-NG was used to perform molecular clock analyses with MCMCTree (Yang and Rannala 2006) in PAML v4.9 (Yang 2007) using the top 10 “clock-like” core single-copy genes, as inferred by SortaDate based on root-to-tip variance (Smith et al. 2018). Divergence times were estimated using the approximate likelihood method (dos Reis and Yang 2011) with the WAG amino acid substitution model (Whelan and Goldman 2001). Due to the sparse fossil record for the fungi at large, a previous fossil-calibrated study of the kingdom including *Fusarium* species was used to inform secondary calibrations of the tree root at 0.9–1.35 (1 time unit being 100 My) and the node representing the split between *F. graminearum* and “*F.*” *solani* at 0.5–0.9 (Lutzoni et al. 2018). For details of MCMCTree priors and run settings, see Supplementary material.

### Computational Prediction of CSEPs and CAZymes

CSEPs were identified from predicted genes using a framework inspired by Beckerson et al. (2019) and summarised in supplementary figure 9*A*, Supplementary Material online including the following: 1) signal peptide detection; 2) filtering for contradictory cellular localisation signals; and 3) cross-checking against machine learning-based effector prediction using EffectorP 3.0 (Sperschneider and Dodds 2021). A custom bash script, CSEPfilter, was written to perform the filtering of gene sets at each stage. To match CSEPs to experimentally verified genes, sequences were searched against the PHI-base database (downloaded 09/02/2022; Urban et al. 2020) using BLAST 2.7.1+ (Camacho et al. 2009). CAZymes were identified from predicted genes using run_dbCAN v3.0.2 (https://github.com/linnabrown/run_dbcan) from the dbCAN2 CAZyme annotation server (Zhang, Yohe, et al. 2018) and assigned names using the ExplorEnz website (McDonald et al. 2009). For full details on both CSEP and CAZyme prediction, see Supplementary material.

CSEPs and CAZymes were matched to gene orthogroups with a custom R script, orthogroup_parser.r, where a gene was defined as a CSEP/CAZyme if it was predicted to be so in at least one taxon. We checked that genome assembly quality did not significantly influence the number of predicted genes, CSEPs or CAZymes by confirming that there was no correlation between assembly N50 (extracted from NCBI metadata for assemblies produced outside this study) and number of genes/CSEPs/CAZymes using the cor.test function in R.

### Comparative Genomics of Lifestyle

Lifestyles of all the strains used in this study were inferred from the host/substrate and other relevant data (such as pathogenicity tests) sourced from the literature, NCBI BioSample metadata, and online culture collection metadata (supplementary table S1, Supplementary Material online). If a strain was reported from a plant host but without sufficient clarification of whether the plant was exhibiting disease symptoms or the fungus was isolated from inside plant tissues, the strain was classified ambiguously as a “plant associate”. In addition to the lifestyle of the specific strains used in the analyses, other lifestyle reports were collected from the literature with the help of the PlutoF platform (Abarenkov et al. 2010) in order to show the range of reported lifestyles for taxa.

The impact of strain lifestyle on CSEP, CAZyme, and all gene content was explored using an approach developed by Mesny and Vannier (2020) which accounts for confounding phylogenetic signal. Full details are described in Supplementary material, as well as a full description of the statistical analyses to test the significant difference in number of strain-specific genes and mean gene copy number between lifestyles.

### Selection Analyses

To assess whether core single-copy genes have evolved under positive selection we used HyPhy v2.5.30 (Kosakovsky Pond et al. 2005), which offers a suite of tools for assessing selective pressures based on the ratio of nonsynonymous to synonymous substitutions (dN/dS)— that is, the ratio of nucleotide substitutions which alter the transcribed amino acid to those that do not. Notably, this approach assumes that synonymous substitutions are selectively neutral. For full details see Supplementary material; briefly, codon alignments and ML trees were run in BUSTED v3.1 (Murrell et al. 2015) and aBSREL v2.2 (Smith et al. 2015) to detect episodic positive selection (dN/dS > 1). We then used Contrast-FEL to compare differences in relative selective pressures between lifestyles (Kosakovsky Pond et al. 2021).

Codon optimisation of all core single-copy genes to the ribosomal protein gene pool (S) was calculated from each taxon’s codon adaptation index (CAI; Sharp and Li, 1987), effective number of codons (Nc) and GC3 values with the get.s function from the tAI v0.2 package (dos Reis et al. 2004). For full details, see Supplementary material. S values were calculated for CSEP, CAZyme, non-CSEP/CAZyme, and all core single-copy genes in turn. To then assess the relationship between codon optimisation and lifestyle range, we calculated Pearson’s correlation on uncorrected data using the cor.test function in R, and used PGLS regression to assess correlation while correcting for phylogenetic signal in the data with the R package nlme v 3.1-152 (Pinheiro et al. 2021). For number of reported lifestyles, only taxa identified to species level were included, and for species with multiple representative strains the mean S value was used. To visualise the relationship between codon optimisation and phylogeny, we used the ordisurf function from the R package vegan v2.5-7 (Oksanen et al. 2019) to fit S values to the PCA of phylogenetic distances produced in comparative analyses above (recreated in R with the vegan prcomp function). See Supplementary material for full details of statistical analyses on the difference in S values between *Fusarium s. str.* and allied genera, as well as between gene types and lifestyles.

The uco function from seqinr v4.2-8 (Charif and Lobry 2007) was used to calculate codon usage bias in terms of relative synonymous codon usage (RSCU)—the ratio of observed codon usage to expected codon usage—for all codons across each taxon, excluding non-redundant codons encoding methionine and tryptophan and stop codons. RSCU values were then normalised using the scale function and used to produce a Euclidean distance matrix with the dist function, which was used for hierarchical clustering of taxa with the hclust function using the average agglomeration method. We compared the topology produced by hierarchical clustering with the RAxML-NG species tree topology by again computing the normalised Robinson–Foulds metric using the RF.dist function from phangorn. We calculated the *P*-value by computing the metric for 1,000 random trees with the same number of taxa against the species tree topology to determine the number of simulations for which the metric was lower (i.e., topologically closer) than that from the hierarchical clustering.

All results were plotted in R v4.0.4 using packages listed in Supplementary material. Scripts of all analyses are available at https://github.com/Rowena-h/FusariumLifestyles.

## Supplementary Material

Supplementary data are available at *Molecular Biology and Evolution* online.

## Supplementary Material

msac085_Supplementary_Data

## Data Availability

WGS data and structurally annotated genome assemblies generated in this study are available on GenBank under the BioProject accession PRJNA761077. Additional data files of the raw phylogenetic trees; CSEP and CAZyme amino acid sequences; OrthoFinder output; and orthogroup metadata are deposited in Zenodo doi:10.5281/zenodo.6353640. All scripts are available at https://github.com/Rowena-h/FusariumLifestyles.
