# Supplementary material for: Lifestyle Transitions in Fusarioid Fungi are Frequent and Lack Clear Genomic Signatures
Source: Mol Biol Evol. 2022 Apr 29;39(4):msac085. doi: 10.1093/molbev/msac085 (PMC9051438; doi:10.1093/molbev/msac085)
Supplement: msac085_Supplementary_Data [file msac085_supplementary_data.docx]

**SUPPLEMENTARY MATERIAL**

Lifestyle transitions in fusarioid fungi are frequent and lack clear genomic signatures

Rowena Hill, Richard JA Buggs, Dang Toan Vu, Ester Gaya

**MATERIAL AND METHODS**

Our bioinformatics analysis pipeline is summarised in supplementary fig. 1. Scripts of all analyses are available at https://github.com/Rowena-h/FusariumLifestyles.

**Comparison of *de novo* assembly tools**

Reads were trimmed using Trimmomatic v0.36 ^1^ and quality checked using FastQC v0.11.5 ^2^. The performance of three *de novo* assembly tools was compared – ABySS v2.0.2 ^3^, MEGAHIT v1.2.9 ^4^ and SPAdes v3.11.1 ^5^. In the case of ABySS, which requires the user to specify k-mer size, multiple assemblies were run with varying k-mer sizes to converge on an optimal k-mer size according to N50, which was calculated with the abyss-fac function. Alternatively, both MEGAHIT and SPAdes use a multiple k-mer sizes strategy. For MEGAHIT assemblies were run with k-mer sizes from 51 to 131 in steps of 8, while for SPAdes the default recommended k-mer sizes for the read length were used: 21, 33, 55, 77. Trimmed reads were mapped back onto contigs using BWA-MEM v0.7.17-r1188 ^6^ and the resulting BAM files then used for polishing with Pilon v1.23 ^7^, which helps to corrects misassemblies and gaps. The flagstat option from SAMtools v1.9 ^8^ was used to produce read mapping statistics from the BAM files in order to calculate sequencing coverage. Contigs shorter than 200bp were removed using seqtk v1.2-r94 (https://github.com/lh3/seqtk) for compliance with NCBI assembly standards.

The ‘best’ assembly was chosen by assessing contiguity via QUAST v5.0.2 ^9^, and completeness as measured by gene sets via BUSCO v3.0.1 ^10^ using the hypocreales_odb10.2019-11-20 lineage dataset of 4,494 single-copy orthologues (<https://busco.ezlab.org/list_of_lineages.html>). The difference in assembly completeness between the three different assembly tools that were tested – ABySS ^3^, MEGAHIT ^4^ and SPAdes ^5^ – was generally minimal, with single-copy BUSCOs ^10^ differing by no more than 0.11% across tools for each strain (supplementary table 6), which can largely be attributed to the high sequencing coverage. QUAST Nx plots ^9^, which show the smallest contig length at which x% of the assembly is contained in contigs of at least that size, found that ABySS produced assemblies with the best contiguity in four out of five cases (supplementary fig. 10). Although no single tool produced the highest completeness or best contiguity statistics for all strains, ABySS was selected as the best-performing tool on-average for consistency’s sake during later biological comparison across strains. Finally, BlobTools v1.1 ^11^ was used to screen the selected ABySS assemblies and confirm the absence of contaminants using the BAM file of mapped reads and a blastn hit file of assemblies against the NCBI nucleotide database created with BLAST 2.7.1+ ^12^ (supplementary fig. 11). Mitochondrial contaminations flagged by NCBI during the assembly submission process were trimmed using bedtools v2.28.0 ^13^.

**Structural annotation with the MAKER pipeline**

A *de novo* repeat library was generated for the ABySS assembly for each strain with RepeatModeler v2.0.1 ^14^ and used as a custom library for softmasking with RepeatMasker v4.0.9 ^15^. Masked assemblies were annotated following the MAKER pipeline ^16^ using proteins and EST clusters downloaded from MycoCosm (https://mycocosm.jgi.doe.gov/; Grigoriev et al. 2014; Mesny et al. 2021) to inform gene prediction: from *F. oxysporum* MPI-SDFR-AT-0094 (Fusoxy1) for *F. chuoi* RH1, *F. chuoi* RH3, *F. annulatum* RH5 and *F. proliferatum* RH7 belonging to the *F. fujikuroi* species complex; and *F. equiseti* MPI-CAGE-AA-0113 (Fuseq1) for *F.* sp. RH6 belonging to the *F. incarnatum-equiseti* species complex. The first evidence-based round of MAKER was used to train SNAP v2006-07-28 ^19^ and the resulting parameters input into a second *ab initio* MAKER round alongside the AUGUSTUS v3.2.3 ^20^ pre-trained parameter set for *Fusarium*. A second iteration of SNAP training and *ab initio* prediction was then performed. Misannotations in the form of artefactually fused genes that were flagged by NCBI during the assembly submission process were checked against existing annotations in NCBI’s Genome Data Viewer ^21^, and then manually edited. For compliance with NCBI standards, Genome Annotation Generator v2.0.1 ^22^ was used with the options -ris 10, --fix_terminal_ns and --fix_start_stop to remove introns shorter than 10bp, remove terminal strings of Ns and ensure start and stop codons were correctly annotated.

**MCMCTree divergence time analyses**

We used uniform node age priors by setting both the birth rate (λ) and death rate (μ) to 1 and the sampling fraction (ρ) to 0. For the substitution rate (r) prior, the shape parameter (α) was set to 1, and the scaling parameter (β) was estimated as 4.5 using the following equation: β = (α × root-time) / tip-to-root, where the mean tip-to-root distance was calculated as 0.22 for both ML species trees using the distRoot function from the package adephylo v1.1-11 ^23^ in R v4.0.4 ^24^, and the root-time being approximately 1 MY as described above. We set the rate drift (σ^2^) prior parameters of α and β to 1 and 10, respectively.

Two MCMC chains were run for both the independent rates (IR) and autocorrelated rates (AR) relaxed clock models, with 20,000 generations, posterior sampling every 10 generations and a 10% burnin per chain. Chain convergence was confirmed by plotting the posterior mean times for both chains for each clock model, and infinite-sites plots were made to confirm that sufficient molecular data was used (supplementary fig. 12).

**Computational prediction of candidate secreted effector proteins (CSEPs) and carbohydrate-active enzymes (CAZymes)**

CSEPs were identified from predicted genes using a framework inspired by Beckerson et al. (2019) and summarised in supplementary fig. 9A, including the following steps:

(1) The putative secretome was identified via prediction of signal peptides with SignalP v5.0b ^26^. Signal peptide prediction was additionally cross-checked against TargetP v2.0 ^27^ and Phobius v1.01 ^28^.

(2) Genes were removed if their predicted cellular localisation contradicted secretion: (i) more than 1 transmembrane domain according to TMHMM v2.0c ^29^ and Phobius; (ii) endoplasmic reticulum retention according to ps_scan v1.86 ^30^; (iii) nuclear localisation according to NucPred v1.1 ^31^; or (iv) GPI-anchored according to PredGPI ^32^, accessed using the R package ragp ^33^.

(3) The remaining genes were cross checked with machine learning-based effector prediction using EffectorP 3.0 ^34^. CSEPs were predominantly less than 300 amino acids in length (supplementary fig. 9B), an oft-quoted cut-off for small secreted proteins in fungi ^35^.

To match CSEPs to experimentally verified genes, sequences were searched against the PHI-base database (downloaded 09/02/2022; Urban et al. 2020) using a blastp search with an e-value of 1e-25 from BLAST 2.7.1+. For CSEPs with multiple successful hits, the hit with the top bitscore was used.

CAZymes were identified from predicted genes using the standalone tool run_dbCAN v3.0.2 (<https://github.com/linnabrown/run_dbcan>) of the dbCAN2 CAZyme annotation server ^37^. This process involved (i) HMMER v3.3.2 ^38^ search against the dbCAN HMM (hidden Markov model) database; (ii) DIAMOND v2.0.14 ^39^ search against the CAZy pre-annotated CAZyme sequence database ^40^ and (iii) eCAMI ^41^ search against a CAZyme short peptide library for classification and motif identification. Only genes which were predicted to be a CAZyme by all three methods were classified as such. Accepted names were retrieved via an automated search of Enzyme Commission (EC) numbers against the ExplorEnz website ^42^ and webscraping of the results using the R package rvest v1.0.2 ^43^. CAZyme families known to act on the major plant cell wall substrates of cellulose, cutin, hemicellulose, lignin and pectin were classified from the literature ^18,44–48^.

**Comparison of lifestyles**

The impact of strain lifestyle on both CSEP-only gene content and all gene content was explored using an approach developed by Mesny and Vannier (2020). This included principal component analysis (PCA) of phylogenetic distances from the dated species tree using the PCA function from the Python package scikit-learn v0.23.2 ^50^ – run in Python v3.7.9 (<https://www.python.org/>) – which was used for global permutational analysis of variance (PERMANOVA) of gene/CSEP content with the adonis2 function from the R package vegan v2.5-7 ^51^ using the model JaccardDistMatrix ~ PC1 + PC2 + Lifestyle. Two principal components were deemed sufficient to represent phylogenetic signal as together they explained over 90% of the variance (see supplementary fig. 13 for PCA plots and variance explained for the first 6 principal components). Pairwise PERMANOVA was then performed using the pairwise.perm.manova function from RVAideMemoire v0.9-78 ^52^ with Bonferroni multiple test correction.

For statistical analyses to test the difference in number of genes; number of strain-specific genes; and mean gene copy number between lifestyles, we first assessed the assumption of normality by making Q-Q plots using the ggqqplot function from ggpubr v0.4.0 ^53^ to ascertain approximate normality of residuals. We then assessed the assumption of homogeneity of variance using the levene_test function from the package rstatix v0.7.0 ^54^, where a significant p value (p < 0.05) means that the assumption is violated.

If we could assume homogeneity of variance (i.e., Levene’s p ≥ 0.05), we used the rstatix function anova_test to compute analysis of variance (ANOVA) using the model value ~ PC1 + PC2 + lifestyle, as with the PERMANOVA, to once again account for phylogeny. If the ANOVA was significant (p < 0.05), a multiple comparison test between lifestyles was performed with the tukey_hsd rstatix function using the model value ~ lifestyle. If we could not assume homogeneity of variance (i.e., Levene’s p < 0.05), we used an aligned rank transform (ART) ANOVA with the aligned.rank.transform function from the ART v1.0 ^55^ R package, again using the model value ~ PC1 + PC2 + lifestyle. If the ART ANOVA was significant, the games_howell_test rstatix function was used for multiple comparison testing using the formula value ~ lifestyle, as is recommended for multiple comparisons when classical ANOVA assumptions are violated ^56^.

**dN/dS analyses**

To produce the codon alignments necessary to calculate dN/dS, nucleotide sequences corresponding to the 1,060 core single-copy genes used in the phylogenomic analysis were retrieved from MAKER outputs and – for previously published taxa – GBFF files, using a custom Python script, pull_nucleotides.py. Occasionally, the corresponding nucleotide sequences were the incorrect length, and in these cases they were manually cross-checked with amino acid sequences and trimmed in AliView v1.25 ^57^. Nucleotide sequences were then used to convert amino acid alignments into codon alignments using PAL2NAL v14.0 ^58^ with the -nogap option. Six genes were filtered out due to alignments not having sufficient gapless sites, leaving 1,054 core single-copy genes for selection analyses.

Codon alignments and ML gene trees were run in BUSTED v3.1 ^59^ to detect gene-wide episodic positive selection (dN/dS > 1). To then identify specific lineages under episodic positive selection for each gene, codon alignments were run with the ML species tree in aBSREL v2.2 ^60^, which employs Holm-Bonferroni multiple testing p-value correction. For both methods, all ingroup lineages were selected as foreground branches for testing. The significant difference in number of genes undergoing positive selection on external branches between different lifestyles was statistically tested as described above, the ANOVA model being value ~ PC1 + PC2 + lifestyle.

To assess whether the inferred positive selection of core CSEPs on external branches could be associated with lifestyle, we used Contrast-FEL to compare differences in relative selective pressures between lifestyles ^61^. This method finds site-level differences in dN/dS between two sets of branches, and so for each gene tree we labelled branches associated with each lifestyle in turn as the ‘test’ set and all other lifestyles as the ‘background’ set. Only external branches were labelled, as we cannot definitively know the lifestyle of common ancestors associated with internodes in the tree. The labelled trees were then run with codon alignments in Contrast-FEL to calculate sites with higher or lower selective pressure in the test set relative to the background set. We used the most conservative statistic reported by Contrast-FEL to determine differences in selective pressures, the multiple testing corrected q-value (p ≤ 0.05, false discovery rate ≤ 0.2), which has the highest precision but lowest recall ^61^. The significant difference in number of sites with a higher or lower relative selective pressure between lifestyles was statistically tested using the same process as described above, using the model sites ~ lifestyle.

**Codon optimisation analyses**

To assess the extent to which selection was acting on synonymous substitutions, we first calculated the codon adaptation index (CAI; Sharp and Li, 1987), which compares codon usage in a given gene to a reference set of highly expressed genes. For our reference set, known ribosomal protein genes were extracted from the functionally annotated protein set of *F. graminearum* PH1 ^63^ downloaded from Mycocosm ^17^ with the getfasta tool from bedtools v2.28.0 ^13^. These ribosomal protein genes were used as input for a blastp search against the predicted genes of all taxa used in this study using BLAST 2.7.1+, and then matched to core single-copy genes with a custom R script, codon_optimisation.r. A gene was defined as encoding a ribosomal protein if it had a blast hit in at least 1 taxon. The codonTable function from the coRdon v1.1.3 ^64^ R package was used to produce a table of codon counts for each core single-copy gene for each taxon, from which CAI was calculated in reference to the identified ribosomal protein genes using the CAI function from coRdon.

The effective number of codons (Nc) was also calculated from the codon count table using the ENC function from coRdon, and GC content at the third codon position (GC3) was calculated using the GC function from seqinr v4.2-8 ^65^. Codon optimisation of all core single-copy genes to the ribosomal protein gene pool (S) was calculated from each taxon’s CAI, Nc and GC3 values with the get.s function from the tAI v0.2 package ^66^. S values were calculated for CSEP, CAZyme, non-CSEP/CAZyme and all core single-copy genes in turn. The significant difference in S values between lifestyles and between gene types (i.e., CSEP, CAZyme or other) for each lifestyle was statistically tested using the same process as described above, the ANOVA/ART ANOVA model being S ~ PC1 + PC2 + lifestyle and TukeyHSD/Games Howell test model being S ~ lifestyle.

To assess the relationship between codon optimisation and number of reported lifestyles, we calculated Pearson’s correlation on uncorrected data using the cor.test function in R, and used phylogenetic generalised least squares (PGLS) regression to assess correlation while correcting for phylogenetic signal in the data with the R package nlme v 3.1-152 ^67^. For PGLS, which specifies that trait covariance between pairs of taxa decreases with time since divergence, we tested Brownian, Pagel and Blomberg phylogenetic correlation structures for the dated species tree, implemented in the R package ape v5.6-1 ^68^, and selected Brownian as the best model fit based on AIC values. For number of reported lifestyles, only taxa identified to species level were included, and for species with multiple representative strains the mean S value was used. To assess the relationship between codon optimisation and phylogeny, we used the vegan ordisurf function to fit S values to the PCA of phylogenetic distances produced in comparative analyses above (recreated in R with the vegan prcomp function). The significant difference in overall S values between *Fusarium* *s. str.* and allied genera was tested using the t.test function in R (having confirmed normality of residuals and homogeneity of variance with a Q-Q plot and Levene’s test as above).

**Data visualisation**

All results were plotted in R v4.0.4 using the packages ape v5.6-1 ^68^, cowplot v1.1.1 ^69^, deeptime v0.0.6.0 ^70^, dendextend v1.15.2 ^71^, dplyr v1.0.6 ^72^, eulerr v6.1.0 ^73^, ggplot2 v3.3.3 ^74^, ggalluvial v0.12.3 ^75^, ggforce v0.3.2.9000 ^76^, ggnewscale v0.4.6 ^77^, ggplotify v0.0.7 ^78^, ggpubr v0.4.0 ^53^, ggrepel v0.9.1 ^79^, ggthemes v4.2.4 ^80^, ggtree v2.4.2 ^81^, jsonlite v1.7.2 ^82^, matrixStats v0.61.0 ^83^, MCMCtreeR v1.1 ^84^, metR v0.9.2 ^85^, multcompView v0.1-8 ^86^, pBrackets v1.0.1 ^87^, phytools v0.7-80 ^88^, plyr v1.8.6 ^89^, reshape2 v1.4.4 ^90^, scales v1.1.1 ^91^, stringi v1.6.2 ^92^, stringr v1.4.0 ^93^ and tidyr v1.1.3 ^94^. R scripts were written using RStudio v1.3.1093 ^95^. This research utilised Queen Mary's Apocrita HPC facility, supported by QMUL Research-IT ^96^.

**Supplementary Table 1.** Metadata for all the strains used in this study. Lifestyle reports were excluded if the taxonomic classification relied solely on ITS sequences, which is an unreliable barcode for *Fusarium* spp. ^97^. Names of strains that were WGS and assembled in this study are in bold.

| Species | Assembly accession | Strain/Voucher | Host/substrate | Lifestyle | Other reported lifestyles |
| --- | --- | --- | --- | --- | --- |
| *Albonectria albosuccinea* =*Fusarium albosuccineum* | GCA_012931995.1 | NRRL 20459 | tree | **plant associate** ^98^ | **endophyte** (*Nectandra lineatifolia* (as Nectriaceae) ^99^)  **saprotroph** (sterile *Nectandra lineatifolia* wood (as Nectriaceae) ^99^) |
| *Albonectria rigidiuscula* =*Fusarium decemcellulare* | GCA_013266205.1 | NRRL 13412 | *Coffea* sp. | **plant pathogen** ^100^ | **endophyte** (*Theobroma gileri* ^101^)  **plant pathogen** (*Dimocarpus longan*, *Mangifera indica, Nephelium lappaceum* ^102^; *Malus pumila* ^103^; *Magnolia denudata* ^104^; *Persea americana* ^105^) |
| *Fusarium acutatum* | GCA_012932015.1 | NRRL 13308 | ? | **plant associate** ^106^ | **animal associate** (*Homoptera* sp. ^106^)  **human pathogen** ^107–109^  **plant associate** (*Cajanus* sp., *Triticum* sp. ^106^)  **plant pathogen** (*Cyamopsis tetragonoloba*) |
| *Fusarium agapanthi* | GCA_001654555.2 | NRRL 31653 | *Agapanthus praecox* | **plant pathogen** ^110^ | **plant pathogen** (*Agapanthus africanus* ^111^) |
| *Fusarium annulatum* ET1^[[1]](#footnote-2)^ | GCA_900067095.1 | ET1 | *Dendrobium moschatum* | **endophyte** ^112^ | **animal associate (***Hylurgops palliatus* ^113^)  **endophyte** (*Austrostipa aristiglumis* ^114^; grapevine ^115^; *Hevea brasiliensis* ^116^; *Lilium longiflorum* ^117^)  **human pathogen** ^118^  **mycoparasite** (Smut*, Stereum hirsutum*^119^)  **plant pathogen** (*Albizia julibrissin* ^120^; *Allium cepa* ^121^; *Allium sativum* ^122^; *Allium tuberosum* ^123^; *Cannabis sativa* ^124^; *Carthamus tinctorius* ^125^; *Colchicum kotschyi* ^126^ ; *Echeveria desmetiana* ^127^; *Gladiolus* spp. ^128^; *Gypsophila paniculata* ^129^; *Ilex cornuta* ^130^; *Laelia* spp. ^131^; *Lilium longiflorum* ^117^; *Malus sieversii* ^132^; *Musa* ABB ^133^; *Oryza sativa* ^134^; *Polygonatum cyrtonema* ^135^; *Prunus persica* ^136^; *Sansevieria trifasciata* ^137^*;* sunflower ^138^; *Vaccinium corymbosum* ‘O'Neal’ ^139^)  **saprotroph** (*Arctoscopus japonicus* egg masses ^140^; buried *Cunninghamia lanceolata* sticks ^141^; petroleum-contaminated soil ^142^; washing machines ^143^) |
| *Fusarium annulatum* Fp A8 ^a^ | GCA_003615215.1 | Fp_A8 | *Allium cepa* | **plant pathogen** ^144^ |  |
| ***Fusarium annulatum* RH5** | GCA_022627115.1 | RH5  880149-04 | *Musa itinerans* | **endophyte** (seed) ^145^ |  |
| *Fusarium anthophilum* | GCA_013364935.1 | NRRL 25214 | *Hippeastrum* sp. | **plant associate** ^146^ | **endophyte** (*Austrostipa aristiglumis* ^114^; *Vigna unguiculata* ^147^)  **plant pathogen** (millets ^148^; sunflower ^149^) |
| *Fusarium austroafricanum* | GCA_012932025.1 | NRRL 53441 | plant debris in soil | **saprotroph** ^150^ | **endophyte** (*Pennisetum clandestinum*) ^150^ |
| *Fusarium austroamericanum* | GCA_013364965.1 | NRRL 2903 | polypore fungus | **mycoparasite** ^151^ | **plant pathogen** (*Hordeum vulgare* ^152^; wheat ^153,154^) |
| *Fusarium avenaceum* | GCA_000769215.1 | Fa05001 | *Hordeum vulgare* | **plant pathogen** ^155^ | **animal pathogen** (*Astacus astacus* ^156^; *Austrostipa aristiglumis* ^114^; *Sitophilus oryzae* ^157^)  **endophyte** (*Abies alba* ^158^; *Cucurbita maxima* ^159^; *Lilium longiflorum* bulb ^117^; *Salicornia europaea* ^160^)  **plant associate** (*Salix* spp. ^161^)  **plant pathogen** (*Actinidia chinensis* var. *chinensis* and var. *deliciosa* ^162^; *Allium giganteum* ^163^; *Cucurbita maxima* ^159^; *Glycine max* ^164^; Malus sieversii ^132^; *Lepidium meyenii* ^165^; *Lupinus angustifolius* ^166^*; Pisum sativum* ^167^;  *Racomitrium japonicum* ^168^; *Tanacetum cinerariifolium* ^163^)  **saprotroph** (*Arctoscopus japonicus* egg masses ^140^; burnt *Pinus mugo* stumps ^169^; saline/acidic soil ^170^) |
| *Fusarium beomiforme* | GCA_002980475.2 | NRRL 25174 | soil | **saprotroph** ^171^ | **plant associate** (*Sorghum bicolor* stalk ^172^)  **plant pathogen** (wheat cultivar Norm ^173^) |
| *Fusarium bulbicola* | GCA_013758895.1 | NRRL 25176 | *Nerine bowdenii* | **plant pathogen** ^106^ | **plant pathogen** (*Glycine max* roots ^174^; *Haemanthus and Vallota* bulbs ^106^)  **endophyte** (*Euterpe oleracea* (as *F. sacchari* var. *elongatum)* ^175^; *Xanthorrhoea* ^176^)  **saprotroph** (soil ^176,177^) |
| ***Fusarium chuoi* RH1** | GCA_022627125.1 | RH1  836515-16 | *Musa itinerans* | **endophyte** (seed) ^145^ |  |
| ***Fusarium chuoi* RH3** | GCA_022627105.1 | RH3  836445-12-1 | *Musa itinerans* | **endophyte** (seed) ^145^ |  |
| *Fusarium circinatum* | GCA_013396185.1 | NRRL 25331 | *Pinus radiata* | **plant pathogen** ^106^ | **animal associate** (*Brachyderes incanus*, *Hylastes attenuatus*, *Hylurgops palliatus*, *Hypothenemus eruditus, Ips sexdentatus*, *Orthotomicus erosus, Pityophthorus pubescens* ^113^)  **endophyte** (*Zea mays* ^178^)  **plant pathogen** (*Solanum lycopersicum* ^179^) |
| *Fusarium coffeatum* | GCA_003316985.1 | FIESC_28 | *Sorghum bicolor* | **plant associate** ^180^ | **endophyte** (Carapichea ipecacuanha leaves and roots (as F. chlamydosporum var. fuscum) ^181^)  **plant associate** (*Cynodontis lemfuensis* (as F. chlamydosporum var. fuscum) ^182^)  **plant pathogen** (*Penniscti* *dandestini* (as F. chlamydosporum var. fuscum) ^182^  **saprotroph** (soil (as F. chlamydosporum var. fuscum) ^182^) |
| *Fusarium coicis* | GCA_013781345.1 | NRRL 66233 | *Coix gasteenii* | **endophyte** ^183^ |  |
| *Fusarium culmorum* | GCA_003033665.1 | PV | soil | **saprotroph** ^184^ | **animal associate** (*Hypothenemus eruditus* , *Orthotomicus erosus* ^113^; *Placospongia intermedia* ^185^)  **endophyte** (*Austrostipa aristiglumis* ^114^; *Citrus sinensis* xylem ^186^; *Leymus mollis* ^187^)  **mycoparasite** (*Verticillium dahlia* ^188^)  **plant associate** (*Ammophila arenaria* ^189^; *Salix* spp. ^161^)  **plant pathogen** (*Brassica napus* ^190^; *Cucurbita maxima* ^159^; *Hordeum distichon*, *Hordeum vulgare,Triticum aestivum*, *Triticum turgidum* var. *durum* ^191^; oat cv. Gerald and wheat cv. Claire ^192^; *Solanum tuberosum* ^193^)  **saprotroph** (saline soil ^194^) |
| *Fusarium denticulatum* | GCA_013396175.1 | NRRL 25311 | *Ipomoea batatas* | **plant pathogen** ^106^ | **endophyte** (*Zea mays*) ^195^ |
| *Fusarium flagelliforme* | GCA_003012295.1 | NRRL 13405 | *Zea mays* | **plant pathogen** ^196,197^ | **plant associate** (*Hordeum vulgare* ^198^; *Pinus nigra*, *Thuja* sp., wheat ^199^) |
| *Fusarium fujikuroi* | GCF_900079805.1 | IMI 58289 | *Saccharum officinarum* | **plant pathogen** ^200,201^ | **endophyte** (*Debregeasia salicifolia* ^202^; *Glycine max* ^203^)  **human pathogen** ^118^  **plant pathogen** (*Aspidosperma polyneuron* ^204^; *Bletilla striata* ^205^; *Canna edulis* ^206^; *Lactuca serriola* ^207^; *Lasia spinosa* ^208^; *Oryza sativa* ^134^; plum ^209^; millets ^148^)  **saprotroph** (*Arctoscopus japonicus* egg masses ^140^; *Diaphorina citri* cadavers ^210^; washing machines ^143^) |
| *Fusarium gaditjirri* | GCA_013266175.1 | NRRL 45417 FRC M-8754 | *Heteropogon triticeus* | **endophyte** ^211^ |  |
| *Fusarium globosum* | GCA_013396165.1 | NRRL 26131 | *Zea mays* | **plant associate** (seed) ^212^ | **endophyte** (*Austrostipa aristiglumis* ^114^)  **plant pathogen** (*Arundo donax* ^213^; *Hordeum vulgare* ^214^) |
| *Fusarium graminearum* | GCA_000240135.3 | NRRL 31084 | ? | **plant pathogen** ^215^ | **endophyte** (*Cucurbita maxima* ^159^; *Solanum lycopersicum* ^216^)  **plant associate** (*Agarum clathratum* (marine) ^217^; *Rumohra adiantiformis* ^218^)  **plant pathogen** (*Avena*, *Hordeum*, *Zea* spp. ^219^; *Glycine max* ^203^; *Ipomoea batatas* ^220^; *Oryza sativa* cv. Doongara ^221^; *Setaria italica* ^222^; *Solanum tuberosum* ^193^)  **saprotroph** (*Arctoscopus japonicus* egg masses ^140^) |
| *Fusarium heterosporum* | GCA_013396295.1 | NRRL 20693 | *Claviceps purpurea* | **mycoparasite** ^223^ | **endophyte** (*Austrostipa aristiglumis* ^114^)  **saprotroph** (soil ^224^) |
| *Fusarium langsethiae* | GCA_001292635.1 | Fl201059  9821-16-1 | *Avena sativa* | **plant associate** (seed) ^225^ | **endophyte** (oat cv. Gerald and wheat cv. Claire ^192^ ^[[2]](#footnote-3)^)  **plant pathogen** (barley, oat, wheat ^155^) |
| *Fusarium longipes* | GCA_003012285.1 | NRRL 20695 | soil | **saprotroph** ^226^ | **endophyte** (Musa sp. var. Pisang Awak pseudostem ^227^)  **mycoparasite** (*Sclerospora graminicola* ^228^)  **plant pathogen** (wheat roots and stalks ^229^) |
| *Fusarium mangiferae* | GCA_900044065.1 | MRC7560 | *Mangifera indica* | **plant pathogen** ^230^ | **endophyte** (*Sansevieria trifasciata* ^137^*)* |
| *Fusarium mexicanum* | GCA_013396015.1 | NRRL 53147 | *Mangifera indica* | **plant pathogen** ^231^ | **plant pathogen** (*Swietenia macrophylla* ^232^) |
| *Fusarium mundagurra* | GCA_013396205.1 | NRRL 66235 | soil | **saprotroph** ^183^ | **human pathogen** ^233^  **plant associate** (*Mangifera indica*) ^183^ |
| *Fusarium napiforme* | GCA_013396005.1 | NRRL 25196 | millet | **endophyte** (seed) ^234,235^ | **endophyte** (*Rhizophora mucronate)* ^236^  **human pathogen** ^237^  **plant associate (**Sorghum caffrorum ^234^)  **plant pathogen** (*Cucurbita maxima* ^159^)  **saprotroph** (soil ^234^) |
| *Fusarium nygamai* | GCA_002894225.1 | CS10214 | *Triticum* sp. | **endophyte** ^238^ | **endophyte** (*Austrostipa aristiglumis* ^114^; *Solanum lycopersicum* roots ^239^)  **human pathogen** ^240^  **plant associate** (*Phaseolus vulgaris* ^241^)  **plant pathogen** (*Oryza sativa* ^242^; millets ^148^); *Sorghum* ^241,243^; *Solanum tuberosum* ^244^; *Striga hermonthica* ^245^)  **saprotroph** (soil ^241^; petroleum-  contaminated soil ^142^) |
| *Fusarium odoratissimum* = *Fusarium oxysporum* f. sp. *cubense* TR4 | GCA_000350365.1 | Foc4_1.0 | *Musa* spp. AAA cv. Brazilian | **plant pathogen** ^246^ |  |
| *Fusarium odoratissimum* = *Fusarium oxysporum* f. sp. *cubense* TR4 | GCA_000260195.2 | NRRL 54006 | *Musa* sp. | **plant pathogen** ^247^ |  |
| *Fusarium oxysporum* f. sp. *cepae* | GCA_003615085.1 | FoC_Fus2 | *Allium cepa* | **plant pathogen** ^248,249^ | **endophyte** (>10 crop species ^250,251^) |
| *Fusarium oxysporum* f. sp. *conglutinans* | GCA_014154955.1 | Fo5176 | *Brassica oleracea* | **plant pathogen** ^252^ | **plant pathogen** (*Arabidopsis* ^252^) |
| *Fusarium oxysporum* f. sp. *lycopersici* | GCA_000149955.2 | 4287 | *Solanum lycopersicum* | **plant pathogen** ^253^ |  |
| *Fusarium oxysporum* f. sp. *radicis-lycopersici* | GCA_000260155.3 | 26381 | *Solanum lycopersicum* | **plant pathogen** ^247^ | **plant pathogen** (>30 crop species ^254^) |
| *Fusarium phyllophilum* | GCA_013396025.1 | NRRL 13617 | *Dracaena dermensis* | **plant pathogen** ^106^ | **plant pathogen** (*Aloe arborescens* ^255^; *Gasteria excavata , Sansevieria dooneri*, ^106^) |
| *Fusarium poae* | GCA_001675295.1 | 2516 | *Triticum aestivum* | **plant pathogen** ^256^ | **endophyte** (*Austrostipa aristiglumis* ^114^; oat cv. Gerald, wheat cv. Claire ^192^)  **plant pathogen** (alfalfa, barley, bent grasses, corn, fescue, Kentucky bluegrass, oat, rice, soybean, sunflower, timothy, tomato ^257^) |
| ***Fusarium proliferatum* RH7** | GCA_022627135.1 | RH7  836489-13 | *Musa balbisiana* | **endophyte** (seed) ^145^ | **saprotroph** (soil ^258^) |
| *Fusarium pseudoanthophilum* | GCA_013395995.1 | NRRL 25211 | *Zea mays* | **plant associate** ^259^ | **plant pathogen** (*Capsicum annuum* var. *grossum*, *Capsicum annuum* var. *longum*, *Solanum lycopersicum* ^260^) |
| *Fusarium pseudocircinatum* | GCA_013396035.1 | NRRL 36939 | ? | **plant associate** ^[[3]](#footnote-4)^ | **animal associate** *(Heteropsylla incisa* ^106^)  **human pathogen** ^261^  **endophyte** (*Handroanthus chrysotrichus* ^262^)  **plant associate** (*Oryza sativa* ^263^; *Pinus kesiya, Solanum* sp. ^106^)  **plant pathogen** (*Acacia koa* ^264^; *Mangifera indica* ^231^; *Sansevieria trifasciata* ^137^; *Swietenia macrophylla* ^232^)  **saprotroph** (dead leaves and textile ^106^) |
| *Fusarium pseudograminearum* | GCA_000303195.2 | CS3096 | wheat | **plant pathogen** ^265^ | **endophyte** (*Austrostipa aristiglumis* ^114^)  **plant associate** (barley, oat, *Medicago truncatula*, *Phalaris paradoxa* ^266^)  **plant pathogen** (*Hordeum distichon*, *Hordeum vulgare, Triticum aestivum*, *Triticum turgidum* var. *durum* ^191^)  **saprotroph** (soil ^266^) |
| *Fusarium sarcochroum* | GCA_013266185.1 | NRRL 20472 | *Viscum album* | **plant associate** ^267^ | **endophyte** (*Citrus reticulata* and *Citrus limon* twigs and trunks ^268^) |
| ***Fusarium* sp. RH6** | GCA_022627095.1 | RH6  836490-20 | *Musa itinerans* | **endophyte** (seed) ^145^ |  |
| *Fusarium sporotrichioides* | GCA_003012315.1 | NRRL 3299 | *Zea mays* | **plant pathogen** ^269^ | **endophyte** (*Abies alba* ^158^; *Salicornia europaea* ^160^)  **plant pathogen** (*Glycine max* ^174^; *Malus sieversii* ^132^; sunflower ^149^; *Zea mays* ^270^)  **saprotroph** (*Arctoscopus japonicus* egg masses ^140^) |
| *Fusarium subglutinans* | GCA_013396075.1 | NRRL 66333 | *Zea mays* | **plant pathogen** ^271^ | **endophyte** (*Austrostipa aristiglumis* ^114^)  **plant associate** (*Oryza sativa* cv. Doongara ^221^)  **plant pathogen** (*Aspidosperma polyneuron* ^204^; *Cymbidium hybridum* ^272^; *Helianthus annuus* ^149^; millets ^148^) |
| *Fusarium tjaetaba* | GCA_013396195.1 | NRRL 66243 | *Sorghum interjectum* | **endophyte** ^183^ |  |
| *Fusarium venenatum* | GCA_900007375.1 | A3/5 | soil | **saprotroph** ^273^ | **plant associate** (*Solanum tuberosum* ^193^; *Trifolium subterraneum* ^274^) |
| *Fusarium verticillioides* | GCA_000149555.1 | NRRL 20956 | *Zea mays* | **plant pathogen** ^275^ | **animal associate** (*Brachyderes incanus*, *Hylurgops palliatus*, *Ips sexdentatus*, *Orthotomicus erosus* ^113^)  **endophyte** (*Cucurbita* sp ^159^; *Oryza sativa* cv. Quest ^221^; *Glycine max* ^203^; *Solanum lycopersicum* roots ^239^)  **human pathogen** ^261^  **plant pathogen** (*Aspidosperma polyneuron* ^204^; millets ^148^; *Musa* spp. ^276^; Sorghum ^243^; sugarcane ^277^)  **saprotroph** (buried *Cunninghamia lanceolata* sticks ^141^; raw milk and cheese ^278^) |
| *Geejayessia zealandica* =*Fusarium zealandicum* | GCA_013266195.1 | NRRL 22465 | ? | **plant associate** ^279^ | **plant associate** (Hoheria populnea. Plagianthus sp. bark ^280^) |
| *Ilyonectria sp.* | Ilysp1 | Ilysp1 | Populus deltoides | **endophyte** ^281^ |  |
| *Neocosmospora ambrosia* =*Fusarium ambrosium* | GCA_003947045.1 | NRRL 20438 | *Camelia sinensis* | **insect mutualist** (*Euwallacea* ‘*fornicatus*’) ^282^ | **plant pathogen** (*Camellia sinensis* ^283^) |
| *Neocosmospora euwallaceae* =*Fusarium euwallaceae* | GCA_003957675.1 | UCR1854 | *Persea americana* | **insect mutualist** (*Euwallacea* sp.) ^284^ | **plant pathogen** (>100 tree species ^285^) |
| *Neocosmospora floridana* =*Fusarium floridanum* | GCA_003947005.1 | NRRL 62606 | *Acer negundo* | **insect mutualist** (*Euwallacea interjectus*) ^286^ | **plant pathogen** (*Acer negundo* ^286^) |
| *Neocosmospora kuroshia* =*Fusarium kuroshium* | GCA_003698175.1 | UCR3666 | *Persea americana* | **insect mutualist** (*Euwallacea* sp.) ^287^ | **plant pathogen** (*Acer negundo*, *Albizia julibrissin*, *Baccharis salicifolia*, *Baccharis pilularis*, *Dombeya cacuminum*, *Erythrina humeana*, *Persea americana*, *Populus fremontii*, *Populus nigra*, *Platanus racemosa*, *Quercus agrifolia*, *Quercus suber*, *Ricinus communis*, *Robinia pseudoacacia*, *Salix gooddingii*, *Salix laevigata*, *Salix lasiolepis*, *Tamarix ramosissima* ^288^) |
| *Neocosmospora oligoseptata* | GCA_003946995.1 | NRRL 62579  AF-4 | *Ailanthus altissima* | **insect mutualist** (*Euwallacea validus*) ^284^ | **plant pathogen** ^284^ |
| *Neocosmospora pisi* =*Fusarium vanettenii =Fusarium solani* f. sp. *pisi* | GCA_000151355.1 | NRRL 44580  77-13-4 | Pisum sativum ^[[4]](#footnote-5)^ | **plant pathogen** ^289^ | **endophyte** (*Lathyrus aphaca*, *L. ochrus*, *Lotus pedunculatus,* *Medicago arabica*, *M. polymorpha*, *Trifolium angustifolium*, *T. arvense*, *T. campestre*, *T. repens*, *T. subterraneum*, *Vicia benghalensis*, *V. hirsute*, *V. villosa* ^290^)  **plant pathogen** *(Crotalaria* *ochroleuca*, *Galega officinalis*, *Lathyrus dymenum*, *L.* *gorgoni*, *L. inconspicuus*, *L. ochrus*, *L. sativus*, *L. sylvestris*, *Medicago arabica*, *M. orbicularis*, *Melilotus albus*, *Scorpiurus muricatus*, *Trifolium diffusum*, *T. palaestinum*, *T. subterraneum*, *Trigonella foenum−graecum Vicia articulata*, *V. ervilia*, *V. fulgens*, *V. sativa*, *V. villosa* subsp. *varia* ^290^) |
| *‘Fusarium’* sp. AF-6 | GCA_003947015.1 | NRRL 62590 | *Persea americana* | **insect mutualist** (*Euwallacea* sp.) ^282^ | **plant pathogen** ^282^ |
| *‘Fusarium’ duplospermum* | GCA_003946985.1 | NRRL 62584  AF-8 | *Persea americana* | **insect mutualist** (*Euwallacea* *perbrevis*) ^282,291^ | **plant pathogen** ^282^ |

**Supplementary Table 2.** Voucher and collection information for the strains used in this study. FFSC = *Fusarium fujikuroi* species complex, FIESC = *Fusarium incarnatum-equiseti* species complex, ^T^=type material.

| Name | Species hypothesis ^145^ | Voucher | Species complex | Collection location | Host |
| --- | --- | --- | --- | --- | --- |
| *Fusarium chuoi* | RH1 | CBS 148465  836515-16 | FFSC | Vietnam, Nghệ An Province, Con Cuông District, Châu Khê commune  N19°1'48.73" E104°43'31.97" | *Musa itinerans* (seed) |
| *Fusarium chuoi* | RH3 | CBS 148464^T^  836445-12-1 | FFSC | Vietnam, Hà Tĩnh Province, Hương Sơn District, Sơn Kim commune  N18°25'37.38" E105°12'53.95" | *Musa itinerans* (seed) |
| *Fusarium annulatum* | RH5 | 880149-04 | FFSC | Vietnam, Lào Cai Province, Sa Pa, Hoàng Liên National Park  N22°15'10.2" E103°56'35.5" | *Musa itinerans* (seed) |
| *Fusarium* sp. | RH6 | 836490-20 | FIESC | Vietnam, Nghệ An Province, Thanh Chương District, Thanh Thủy commune  N18°38'14.89" E105°14'50.83" | *Musa itinerans* (seed) |
| *Fusarium proliferatum* | RH7 | 836489-13 | FFSC | Vietnam, Nghệ An Province, Thanh Chương District, Thanh Thủy commune  N18°38'29.8" E105°14'15.87" | *Musa balbisiana* (seed) |

**Supplementary Table 3.** Statistical test results for Levene’s test for homogeneity of residual variance and ANOVA, or ART ANOVA (*****) if Levene’s test was significant (p < 0.05).

|  | Levene’s test | | | | | | ANOVA / ART ANOVA (*****) | | | | |
| --- | --- | --- | --- | --- | --- | --- | --- | --- | --- | --- | --- |
|  | Formula | df1 | df2 | statistic | p | Formula | | Effect | Df | F | p |
| # genes  (fig 2A) | All genes ~ lifestyle | 5 | 55 | 0.90 | 0.5 | All genes ~ PC1 + PC2 + lifestyle | | PC1 | 1 | 1.32 | 0.3 |
|  |  |  |  |  |  |  |  | PC2 | 1 | 14.47 | 4E-04 |
|  |  |  |  |  |  |  |  | lifestyle | 5 | 2.29 | 0.06 |
|  | CSEPs ~ lifestyle | 5 | 55 | 1.52 | 0.2 | CSEPs ~ PC1 + PC2 + lifestyle | | PC1 | 1 | 1.50 | 0.2 |
|  |  |  |  |  |  |  |  | PC2 | 1 | 7.26 | 0.009 |
|  |  |  |  |  |  |  |  | lifestyle | 5 | 2.13 | 0.08 |
|  | CAZymes ~ lifestyle | 5 | 55 | 1.71 | 0.2 | CAZymes ~ PC1 + PC2 + lifestyle | | PC1 | 1 | 0.37 | 0.5 |
|  |  |  |  |  |  |  |  | PC2 | 1 | 6.75 | 0.01 |
|  |  |  |  |  |  |  |  | lifestyle | 5 | 2.15 | 0.07 |
| # strain specific genes  (supp. fig. 4A) | All genes ~ lifestyle | 5 | 55 | 1.13 | 0.4 | All genes ~ PC1 + PC2 + lifestyle | | PC1 | 1 | 3.88 | 0.05 |
|  |  |  |  |  |  |  |  | PC2 | 1 | 0.50 | 0.5 |
|  |  |  |  |  |  |  |  | lifestyle | 5 | 1.10 | 0.4 |
|  | CSEPs ~ lifestyle | 5 | 55 | 0.67 | 0.6 | CSEPs ~ PC1 + PC2 + lifestyle | | PC1 | 1 | 7.73 | 0.007 |
|  |  |  |  |  |  |  |  | PC2 | 1 | 2.25 | 0.1 |
|  |  |  |  |  |  |  |  | lifestyle | 5 | 1.51 | 0.2 |
| Mean gene copy number  (supp. fig. 4B) | All genes ~ lifestyle | 5 | 55 | 2.02 | 0.09 | All genes ~ PC1 + PC2 + lifestyle | | PC1 | 1 | 0.00 | 1 |
|  |  |  |  |  |  |  |  | PC2 | 1 | 3.14 | 0.08 |
|  |  |  |  |  |  |  |  | lifestyle | 5 | 1.76 | 0.1 |
|  | CSEPs ~ lifestyle | 5 | 55 | 1.64 | 0.2 | CSEP ~ PC1 + PC2 + lifestyle | | PC1 | 1 | 0.00 | 1 |
|  |  |  |  |  |  |  |  | PC2 | 1 | 2.53 | 0.1 |
|  |  |  |  |  |  |  |  | lifestyle | 5 | 1.25 | 0.3 |
|  | CAZymes ~ lifestyle | 5 | 55 | 1.75 | 0.1 | CAZymes ~ PC1 + PC2 + lifestyle | | PC1 | 1 | 0.02 | 0.9 |
|  |  |  |  |  |  |  |  | PC2 | 1 | 2.18 | 0.1 |
|  |  |  |  |  |  |  |  | lifestyle | 5 | 1.61 | 0.2 |
| # positively selected genes on external branches | num ~ lifestyle | 5 | 52 | 1.50 | 0.206 | num ~ PC1 + PC2 + lifestyle | | PC1 | 1 | 1.08 | 0.3 |
|  |  |  |  |  |  |  |  | PC2 | 1 | 0.05 | 0.8 |
|  |  |  |  |  |  |  |  | lifestyle | 5 | 0.63 | 0.7 |
| Sites with different relative evolutionary rate (fig. 3B) | (Higher) sites ~ lifestyle | 5 | 867 | 14.94 | 4.00E-14 | (Higher) sites ~ lifestyle * | | lifestyle | 5 | 9.77 | 4E-09 |
|  | (Lower) sites ~ lifestyle | 5 | 142 | 1.64 | 0.2 | (Lower) sites ~ lifestyle | | lifestyle | 5 | 1.61 | 0.2 |
| Codon optimisation between lifestyles (fig. 4A) | S ~ lifestyle | 5 | 55 | 1.97 | 0.1 | S ~ PC1 + PC2 + lifestyle | | PC1 | 1 | 21.84 | 2E-05 |
|  |  |  |  |  |  |  |  | PC2 | 1 | 45.37 | 1E-08 |
|  |  |  |  |  |  |  |  | lifestyle | 5 | 2.95 | 0.02 |
| Codon optimisation between gene types (fig. 4B) | (Endophyte)  S ~ gene type | 2 | 30 | 4.21 | 0.02 | (Endophyte)  S ~ PC1 + PC2 + gene type * | | PC1 | 1 | 0.64 | 0.4 |
|  |  |  |  |  |  |  |  | PC2 | 1 | 0.02 | 0.9 |
|  |  |  |  |  |  |  |  | gene type | 2 | 35.81 | 2E-08 |
|  | (Insect mutualist)  S ~ gene type | 2 | 18 | 1.90 | 0.2 | (Insect mutualist)  S ~ PC1 + PC2 + gene type | | PC1 | 1 | 0.29 | 0.6 |
|  |  |  |  |  |  |  |  | PC2 | 1 | 0.29 | 0.6 |
|  |  |  |  |  |  |  |  | gene type | 2 | 308.38 | 2E-13 |
|  | (Plant associate)  S ~ gene type | 2 | 24 | 1.83 | 0.2 | (Plant associate)  S ~ PC1 + PC2 + gene type | | PC1 | 1 | 2.73 | 0.1 |
|  |  |  |  |  |  |  |  | PC2 | 1 | 2.03 | 0.2 |
|  |  |  |  |  |  |  |  | gene type | 2 | 12.39 | 2E-04 |
|  | (Plant pathogen)  S ~ gene type | 2 | 75 | 5.79 | 0.005 | (Plant pathogen)  S ~ PC1 + PC2 + gene type * | | PC1 | 1 | 14.21 | 3E-04 |
|  |  |  |  |  |  |  |  | PC2 | 1 | 24.25 | 5E-06 |
|  |  |  |  |  |  |  |  | gene type | 2 | 80.18 | 4E-19 |
|  | (Saprotroph)  S ~ gene type | 2 | 15 | 1.28 | 0.3 | (Saprotroph)  S ~ PC1 + PC2 + gene type | | PC1 | 1 | 0.26 | 0.6 |
|  |  |  |  |  |  |  |  | PC2 | 1 | 0.46 | 0.5 |
|  |  |  |  |  |  |  |  | gene type | 2 | 55.57 | 4E-07 |

|  | Formula | Effect | Df | SumOfSqs | R2 | F | p |
| --- | --- | --- | --- | --- | --- | --- | --- |
| All genes | JaccardDistMatrix ~ PC1 + PC2 + lifestyle | PC1 | 1 | 1.04 | 0.20 | 18.88 | 1E-04 |
|  |  | PC2 | 1 | 0.80 | 0.15 | 14.54 | 1E-04 |
|  |  | lifestyle | 5 | 0.45 | 0.09 | 1.62 | 0.0063 |
|  |  | Residual | 53 | 2.91 | 0.56 |  |  |
|  |  | Total | 60 | 5.19 | 1.00 |  |  |
| CSEPs | JaccardDistMatrix ~ PC1 + PC2 + lifestyle | PC1 | 1 | 2.57 | 0.21 | 19.49 | 1E-04 |
|  |  | PC2 | 1 | 1.63 | 0.13 | 12.39 | 1E-04 |
|  |  | lifestyle | 5 | 1.13 | 0.09 | 1.71 | 0.002 |
|  |  | Residual | 53 | 6.99 | 0.57 |  |  |
|  |  | Total | 60 | 12.32 | 1.00 |  |  |
| CAZymes | JaccardDistMatrix ~ PC1 + PC2 + lifestyle | PC1 | 1 | 0.87 | 0.26 | 27.85 | 1E-04 |
|  |  | PC2 | 1 | 0.52 | 0.16 | 16.59 | 1E-04 |
|  |  | lifestyle | 5 | 0.30 | 0.09 | 1.91 | 0.0039 |
|  |  | Residual | 53 | 1.66 | 0.50 |  |  |
|  |  | Total | 60 | 3.35 | 1.00 |  |  |

**Supplementary Table 4.** Statistical test results for the PERMANOVA.

**Supplementary Table 5.** Statistical test results for pairwise multiple comparisons using Tukey HSD or, if Levene’s test was significant (see supplementary table 3), Games Howell test (*****).

|  | Formula | group1 | group2 | estimate | conf.low | conf.high | p.adj |
| --- | --- | --- | --- | --- | --- | --- | --- |
| Sites with different relative evolutionary rate (fig. 3B) | (Higher)  sites ~ lifestyle * | endophyte | insect mutualist | 2.86 | 0.81 | 4.91 | 0.001 |
|  |  | endophyte | mycoparasite | -0.70 | -1.62 | 0.23 | 0.3 |
|  |  | endophyte | plant associate | -0.18 | -1.21 | 0.86 | 1 |
|  |  | endophyte | plant pathogen | 0.57 | -1.19 | 2.32 | 0.9 |
|  |  | endophyte | saprotroph | -0.06 | -1.15 | 1.03 | 1 |
|  |  | insect mutualist | mycoparasite | -3.55 | -5.42 | -1.69 | 2E-06 |
|  |  | insect mutualist | plant associate | -3.03 | -4.96 | -1.11 | 1E-04 |
|  |  | insect mutualist | plant pathogen | -2.29 | -4.67 | 0.09 | 0.07 |
|  |  | insect mutualist | saprotroph | -2.92 | -4.87 | -0.96 | 4E-04 |
|  |  | mycoparasite | plant associate | 0.52 | -0.04 | 1.08 | 0.09 |
|  |  | mycoparasite | plant pathogen | 1.26 | -0.28 | 2.80 | 0.2 |
|  |  | mycoparasite | saprotroph | 0.64 | -0.03 | 1.31 | 0.07 |
|  |  | plant associate | plant pathogen | 0.75 | -0.86 | 2.35 | 0.8 |
|  |  | plant associate | saprotroph | 0.12 | -0.69 | 0.93 | 1 |
|  |  | plant pathogen | saprotroph | -0.63 | -2.27 | 1.02 | 0.9 |
| Codon optimisation  between lifestyles (fig. 4A) | S ~ lifestyle | endophyte | insect mutualist | -0.06 | -0.10 | -0.03 | 8E-05 |
|  |  | endophyte | mycoparasite | 0.01 | -0.05 | 0.07 | 1 |
|  |  | endophyte | plant associate | -0.03 | -0.07 | 0.00 | 0.1 |
|  |  | endophyte | plant pathogen | -0.01 | -0.04 | 0.02 | 1 |
|  |  | endophyte | saprotroph | 0.01 | -0.03 | 0.05 | 1 |
|  |  | insect mutualist | mycoparasite | 0.07 | 0.01 | 0.13 | 0.02 |
|  |  | insect mutualist | plant associate | 0.03 | -0.01 | 0.07 | 0.1 |
|  |  | insect mutualist | plant pathogen | 0.06 | 0.02 | 0.09 | 7E-05 |
|  |  | insect mutualist | saprotroph | 0.07 | 0.03 | 0.12 | 6E-05 |
|  |  | mycoparasite | plant associate | -0.04 | -0.10 | 0.02 | 0.5 |
|  |  | mycoparasite | plant pathogen | -0.01 | -0.07 | 0.04 | 1 |
|  |  | mycoparasite | saprotroph | 0.01 | -0.06 | 0.07 | 1 |
|  |  | plant associate | plant pathogen | 0.02 | -0.01 | 0.05 | 0.2 |
|  |  | plant associate | saprotroph | 0.04 | 0.00 | 0.08 | 0.05 |
|  |  | plant pathogen | saprotroph | 0.02 | -0.02 | 0.05 | 0.7 |
| Codon optimisation between different gene types (fig. 4B) | (Endophyte)  S ~ gene type * | S.CSEP | S.CAZyme | 0.07 | -0.03 | 0.16 | 0.2 |
|  |  | S.CSEP | S.other | -0.21 | -0.26 | -0.16 | 3E-07 |
|  |  | S.CAZyme | S.other | -0.27 | -0.36 | -0.19 | 1E-05 |
|  | (Insect mutualist)  S ~ gene type * | S.CSEP | S.CAZyme | 0.22 | 0.18 | 0.25 | 2E-12 |
|  |  | S.CSEP | S.other | -0.07 | -0.10 | -0.04 | 6E-05 |
|  |  | S.CAZyme | S.other | -0.28 | -0.31 | -0.25 | 4E-14 |
|  | (Plant associate)  S ~ gene type * | S.CSEP | S.CAZyme | 0.00 | -0.12 | 0.11 | 1 |
|  |  | S.CSEP | S.other | -0.19 | -0.31 | -0.08 | 9E-04 |
|  |  | S.CAZyme | S.other | -0.19 | -0.31 | -0.08 | 0.001 |
|  | (Plant pathogen)  S ~ gene type * | S.CSEP | S.CAZyme | 0.07 | 0.01 | 0.13 | 0.03 |
|  |  | S.CSEP | S.other | -0.23 | -0.28 | -0.18 | 4E-11 |
|  |  | S.CAZyme | S.other | -0.30 | -0.34 | -0.26 | 3E-14 |
|  | (Saprotroph)  S ~ gene type * | S.CSEP | S.CAZyme | 0.06 | -0.02 | 0.13 | 0.1 |
|  |  | S.CSEP | S.other | -0.24 | -0.31 | -0.16 | 1E-06 |
|  |  | S.CAZyme | S.other | -0.29 | -0.36 | -0.22 | 8E-08 |

**Supplementary Table 6.** Assembly and annotation statistics for the five *Fusarium* strains sequenced in this study.

|  |  |  | QUAST v5.0.2 | | | | | | | BUSCO v3.0.1 (n=4,494) | MAKER v2.31.9 |
| --- | --- | --- | --- | --- | --- | --- | --- | --- | --- | --- | --- |
|  |  | Coverage | # contigs ≥ 500bp | Largest contig (bp) | Total size (bp) | GC (%) | N50 | L50 | # N's/ 100kbp | Assembly completeness (single-copy BUSCOs) | # annotated gene models |
| *F. chuoi*  RH1 | ABySS v2.0.2 k124 | 286× | 111 | 4,298,088 | 45,254,299 | 46.81 | 1,615,464 | 10 | 11.81 | 4,484 (99.78%) | 13,380 |
|  | MEGAHIT v1.2.9 | 290× | 1,874 | 553,685 | 44,681,473 | 47.2 | 115,300 | 117 | 0 | 4,481 (99.71%) | - |
|  | SPAdes v3.11.1 | 288× | 457 | 1,232,307 | 45,027,201 | 46.98 | 386,992 | 38 | 0 | 4,480 (99.69%) | - |
| *F. chuoi*  RH3 | ABySS v2.0.2 k90 | 304× | 981 | 1,147,506 | 44,348,592 | 47.75 | 214,555 | 56 | 6.01 | 4,485 (99.80%) | 14,313 |
|  | MEGAHIT v1.2.9 | 296× | 525 | 1,641,087 | 45,474,718 | 46.86 | 308,265 | 43 | 0 | 4,484 (99.78%) | - |
|  | SPAdes v3.11.1 | 300× | 956 | 1,149,846 | 44,908,739 | 47.28 | 222,386 | 60 | 0 | 4,486 (99.82%) | - |
| *F. annulatum* RH5 | ABySS v2.0.2 k121 | 317× | 70 | 3,875,036 | 43,829,649 | 48.3 | 1,803,139 | 9 | 4.75 | 4,478 (99.64%) | 12,880 |
|  | MEGAHIT v1.2.9 | 327× | 64 | 2,611,815 | 43,875,029 | 48.29 | 1,638,693 | 11 | 0 | 4,480 (99.69%) | - |
|  | SPAdes v3.11.1 | 325× | 136 | 2,843,665 | 43,842,946 | 48.31 | 1,163,461 | 14 | 0 | 4,479 (99.67%) | - |
| *F*. sp.  RH6 | ABySS v2.0.2 k128 | 298× | 110 | 4,722,096 | 39,424,294 | 47.66 | 1,717,955 | 7 | 3.56 | 4,476 (99.60%) | 11,533 |
|  | MEGAHIT v1.2.9 | 340× | 940 | 1,573,830 | 39,142,705 | 47.79 | 224,512 | 49 | 0 | 4,478 (99.64%) | - |
|  | SPAdes v3.11.1 | 332× | 176 | 1,999,176 | 39,279,569 | 47.7 | 1,117,341 | 14 | 0 | 4,478 (99.64%) | - |
| *F. proliferatum* RH7 | ABySS v2.0.2 k127 | 322× | 107 | 4,142,881 | 44,857,950 | 48.05 | 1,615,920 | 10 | 16.91 | 4,481 (99.71%) | 13,009 |
|  | MEGAHIT v1.2.9 | 286× | 609 | 1,463,918 | 44,731,912 | 48.09 | 324,653 | 43 | 0 | 4,481 (99.71%) | - |
|  | SPAdes v3.11.1 | 282× | 140 | 3,454,284 | 44,751,997 | 48.07 | 1,124,917 | 14 | 0 | 4,481 (99.71%) | - |

| 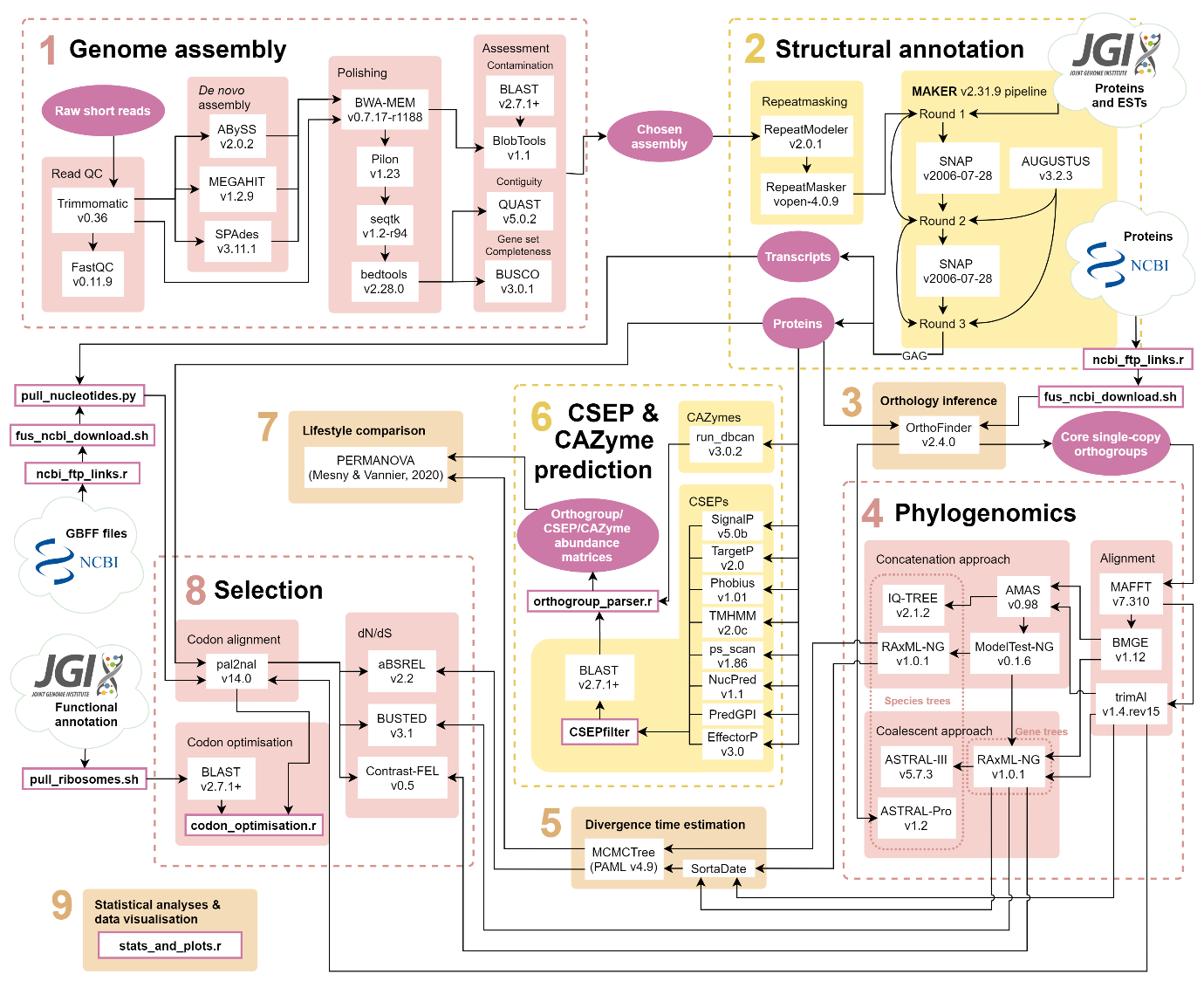 |
| --- |
| **Supplementary Figure 1.** Schematic summarising the bioinformatics analysis pipeline developed in this study, available at https://github.com/Rowena-h/FusariumLifestyles. Boxes outlined in pink indicate custom scripts written for this study. |


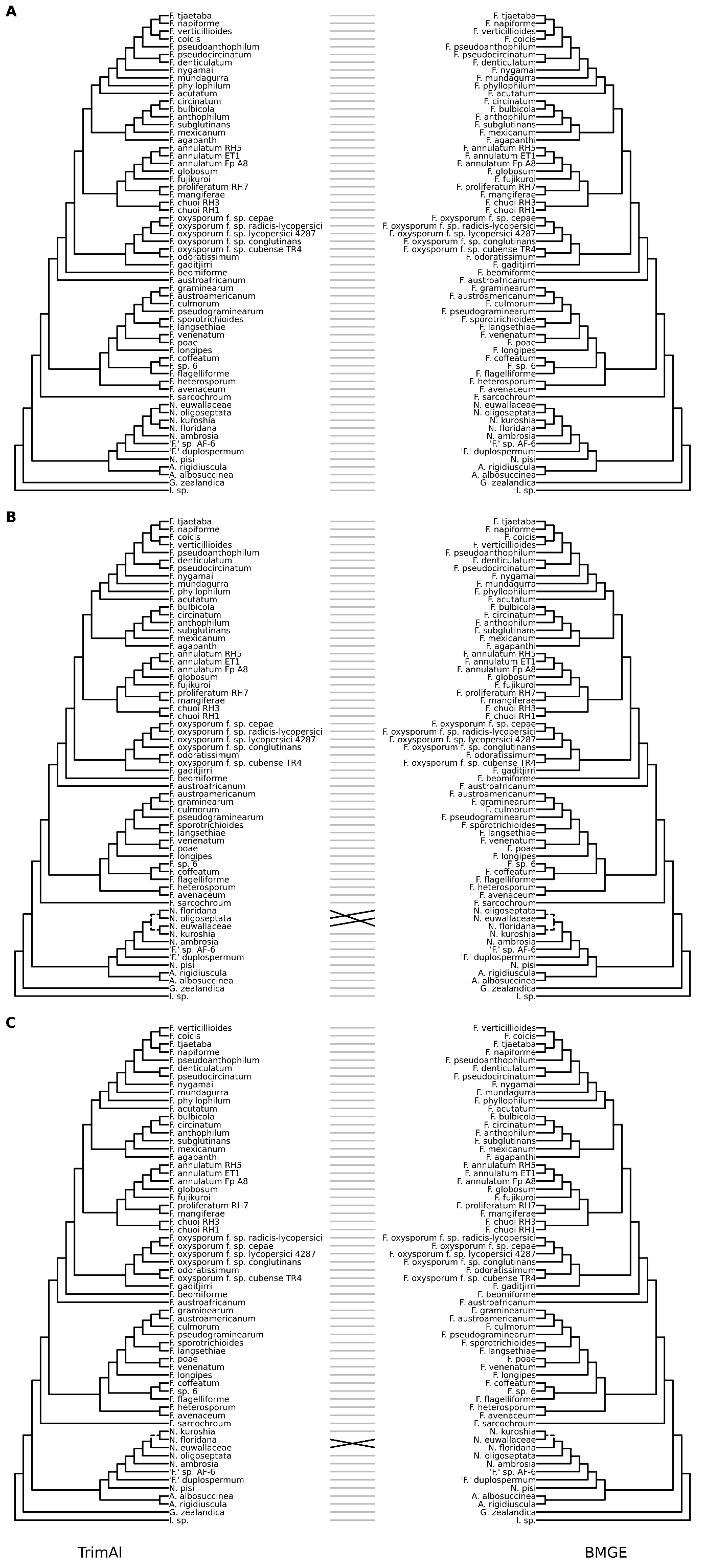

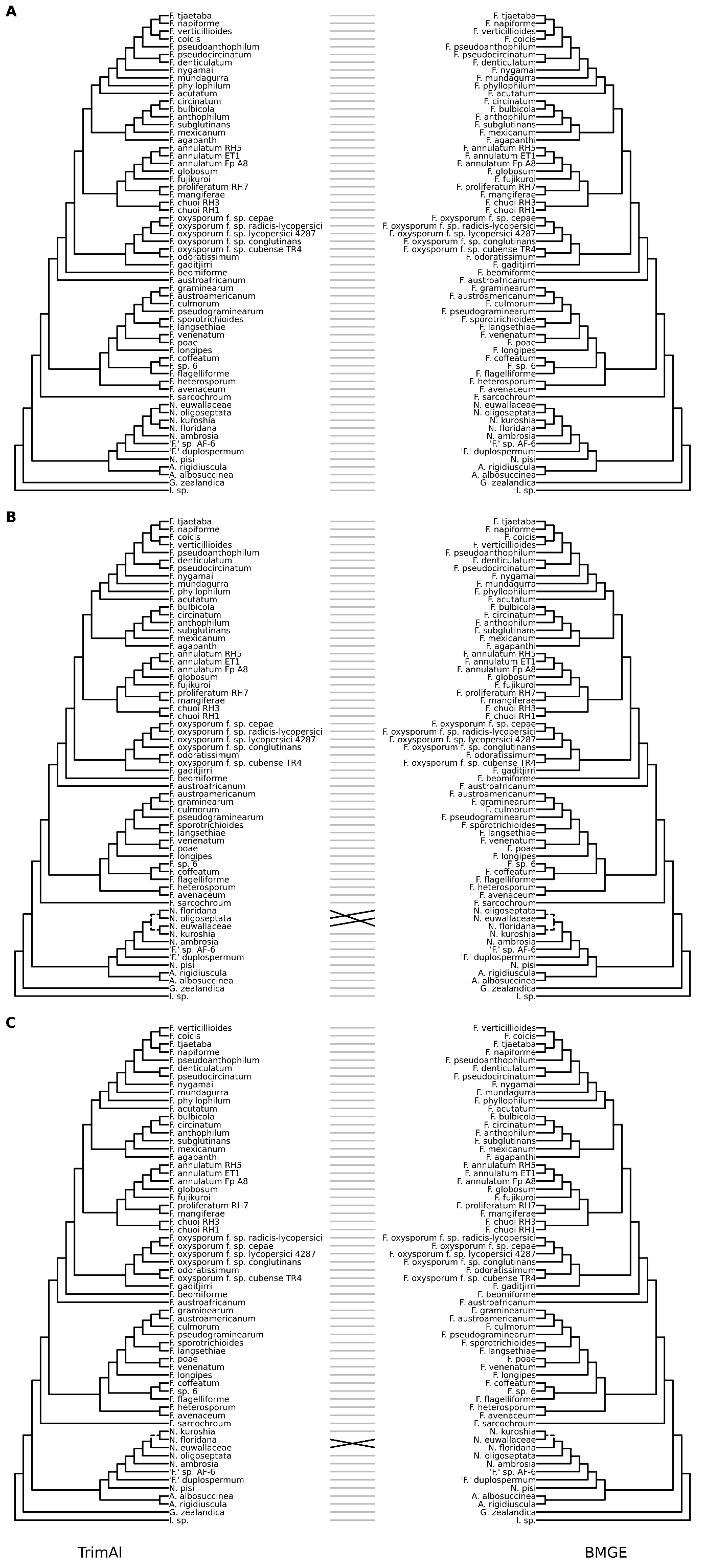
 **Supplementary Figure 2.** Tanglegrams showing the difference in (A) RAxML-NG, (B) IQ-TREE and (C) ASTRAL-III species tree topologies when using different alignment trimming tools: TrimAl (left) and BMGE (right).


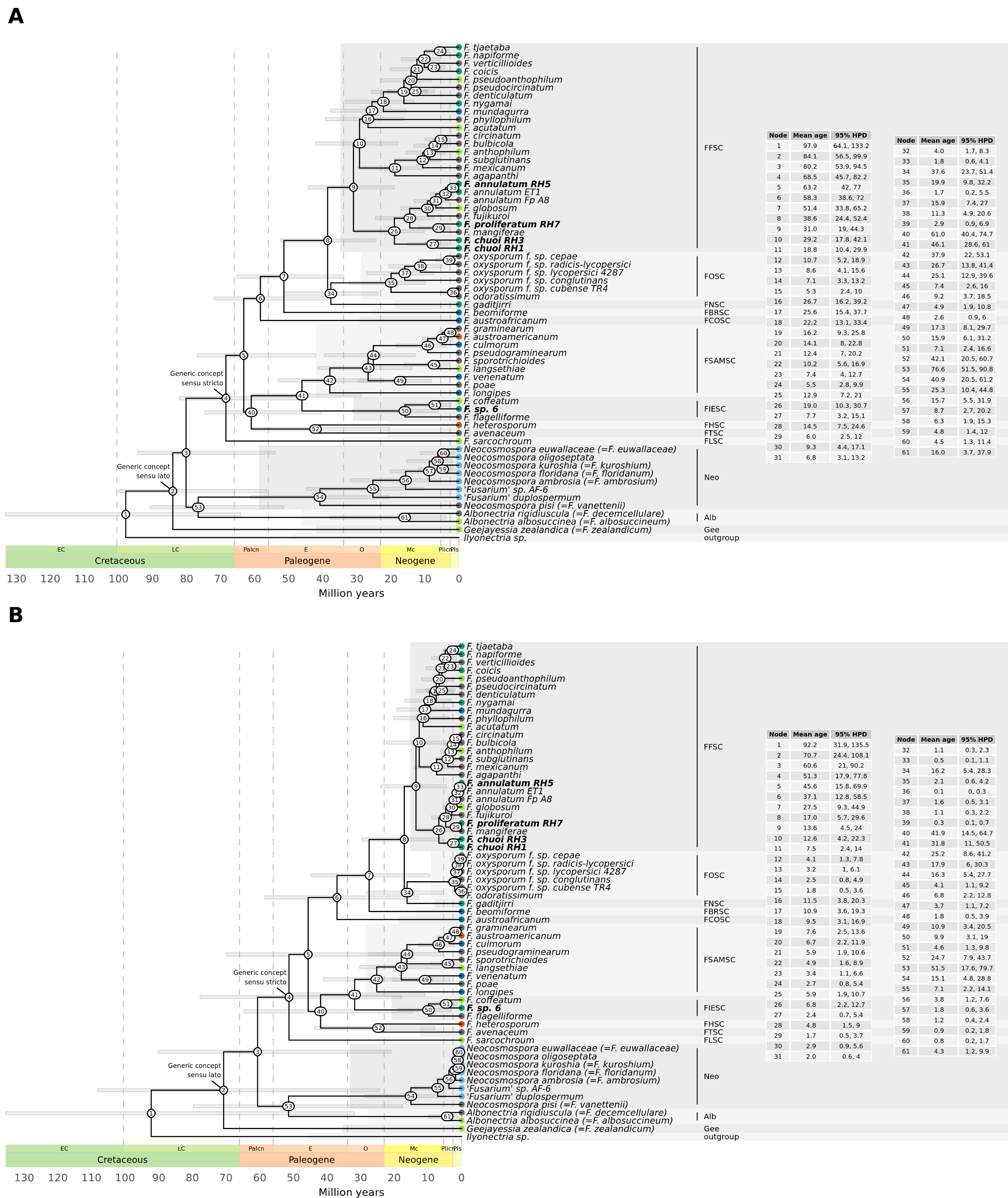


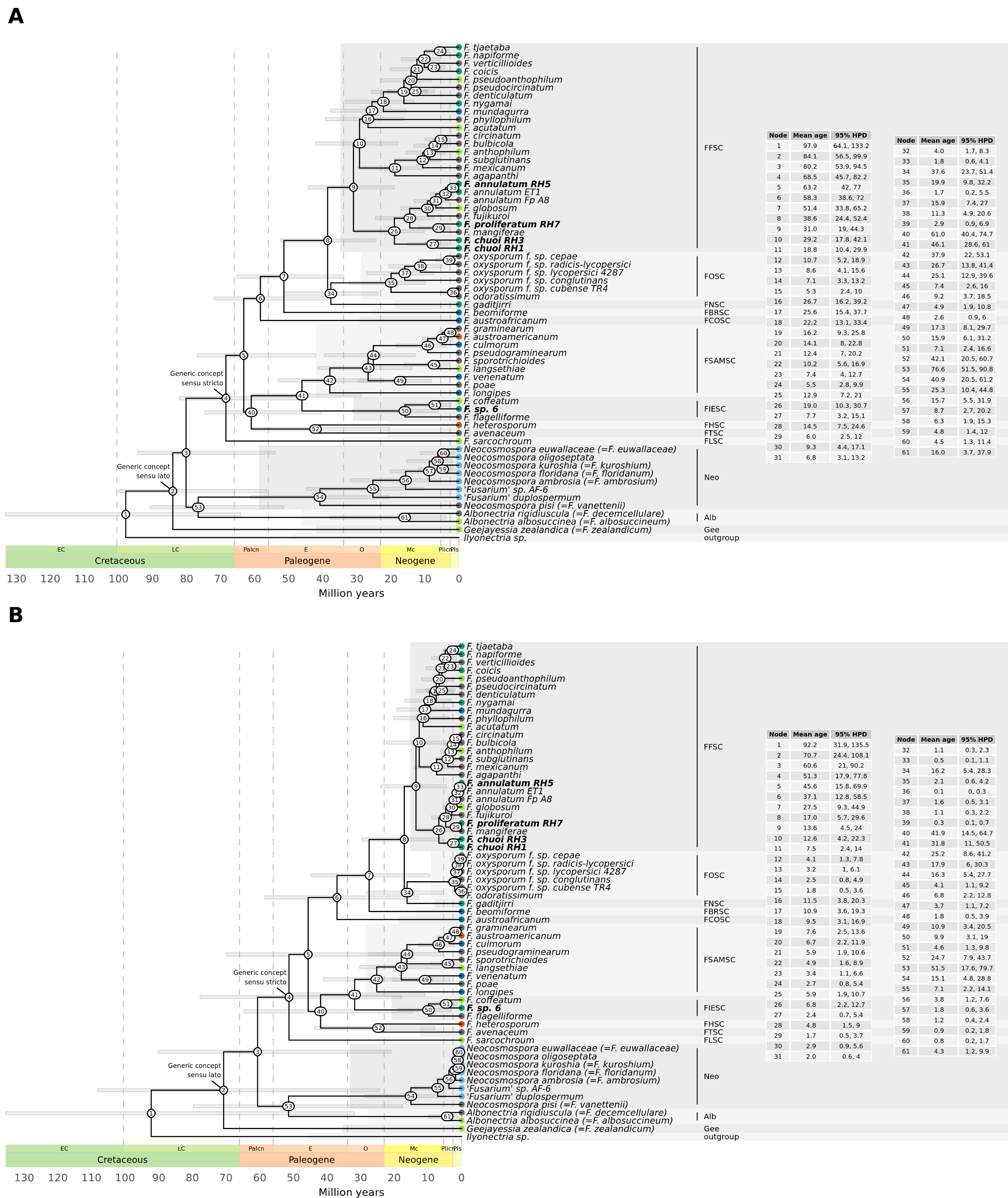
**Supplementary Figure 3.** Mean divergence times and 95% HPD confidence intervals for every node in the phylogeny estimated by MCMCTree using both the AR clock model (A) and the IR clock model (B).


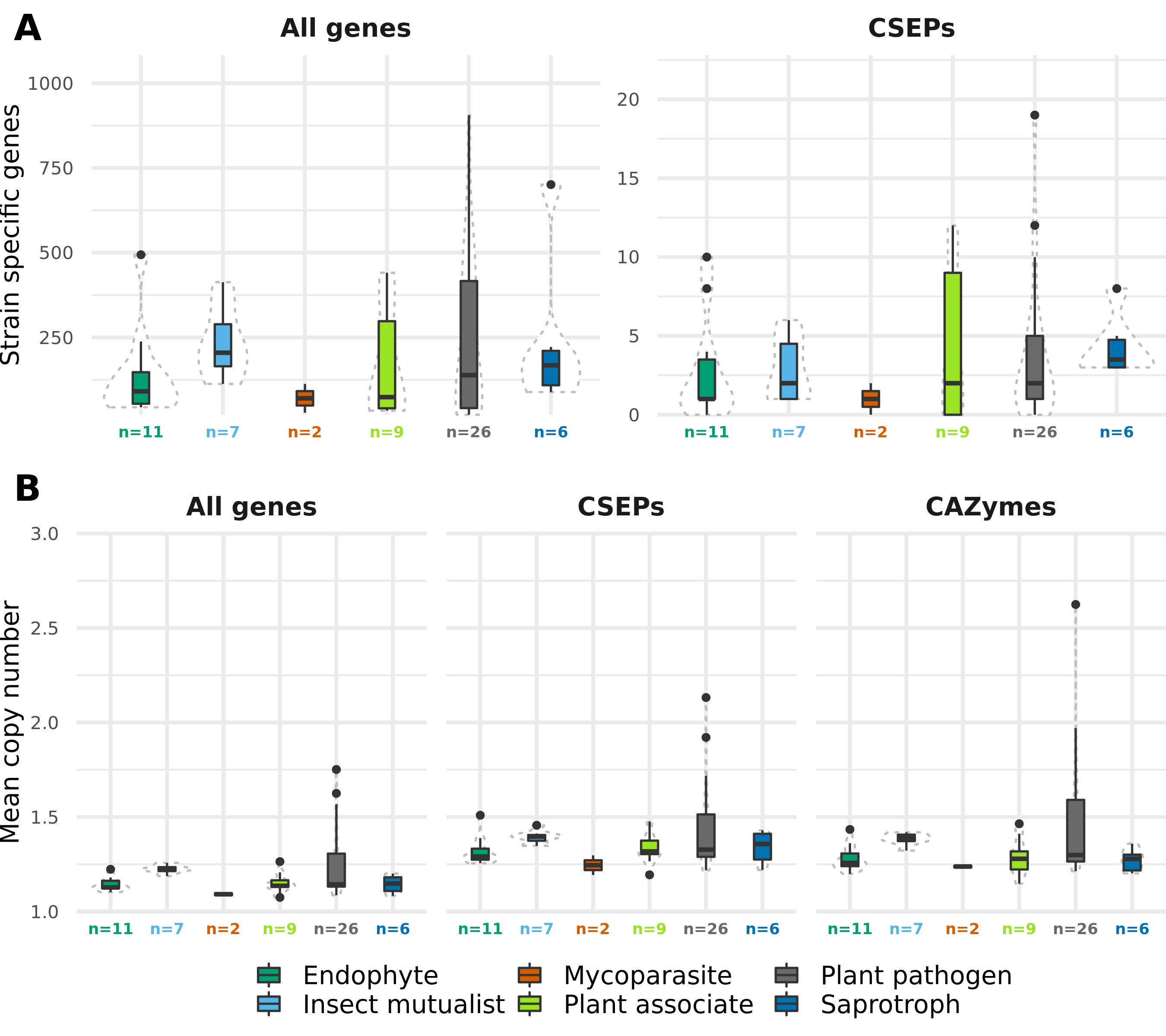


**Supplementary Figure 4.** Boxplots showing the number of strain-specific genes (A) and mean gene copy number (B) for different lifestyles. Sample size (the number of strains) is reported under x axis labels. There were no significant differences according to ANOVA (see supplementary table 3).


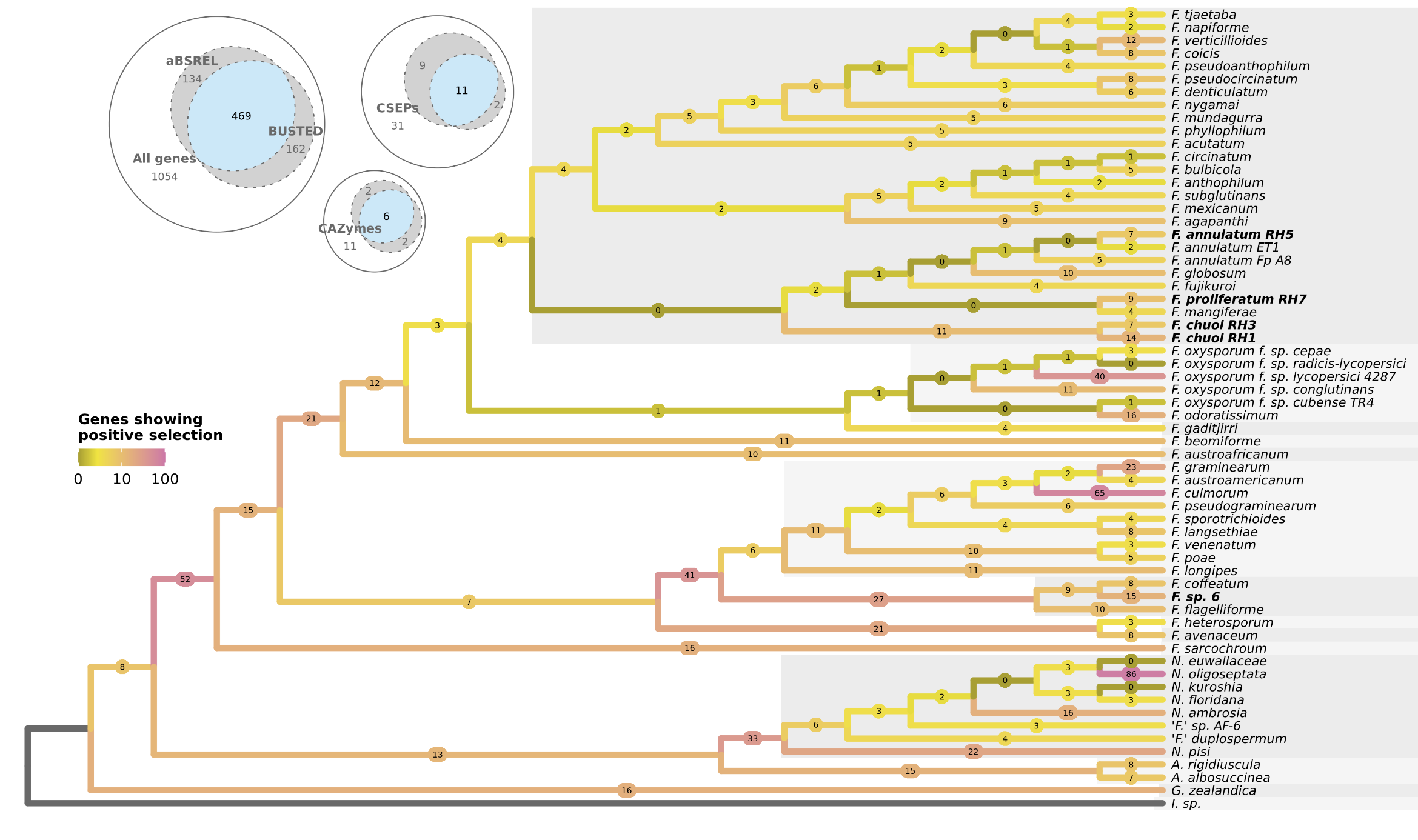


**Supplementary Figure 5.** aBSREL results showing the number of positively selected genes for every branch of the dated species tree.


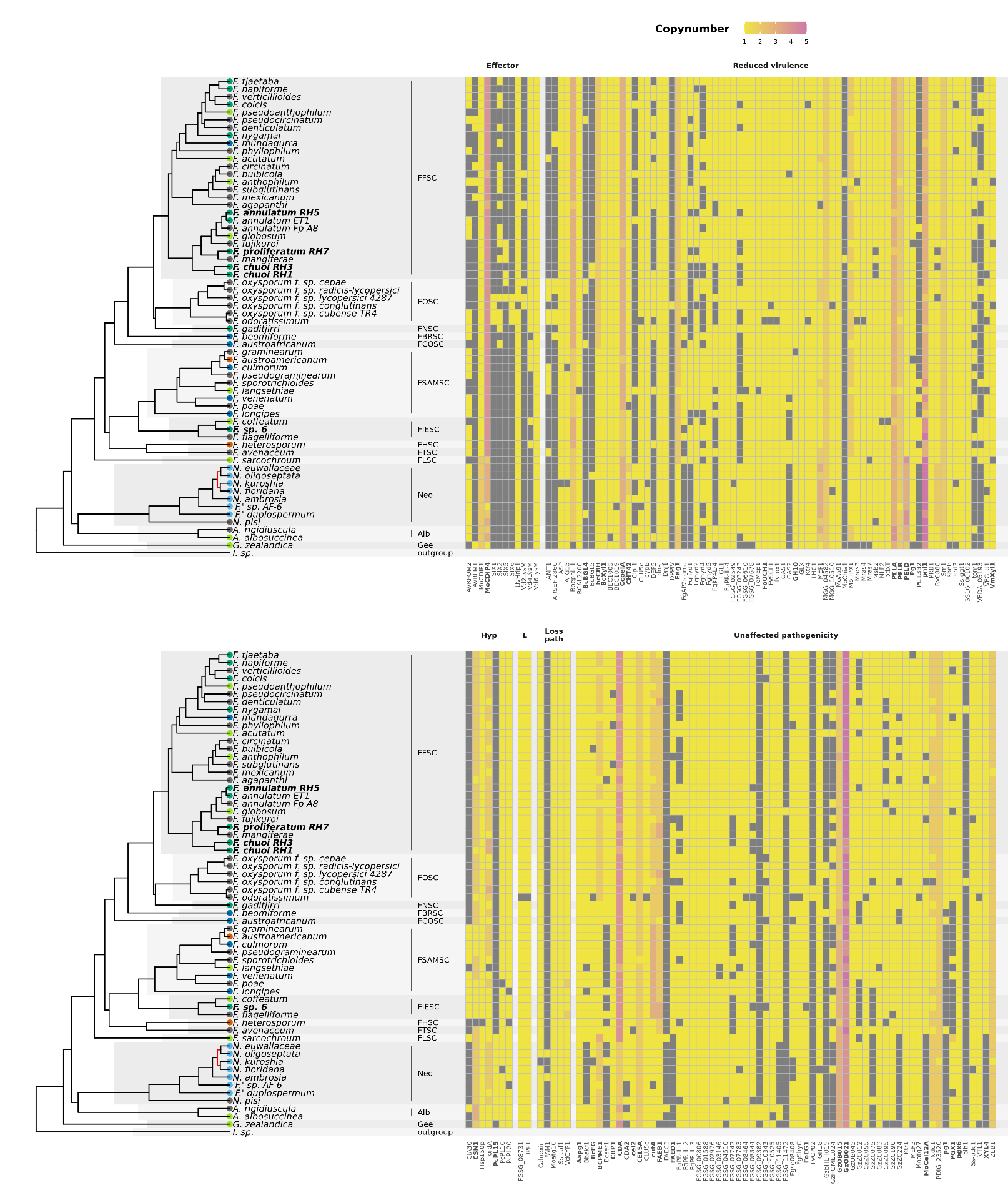

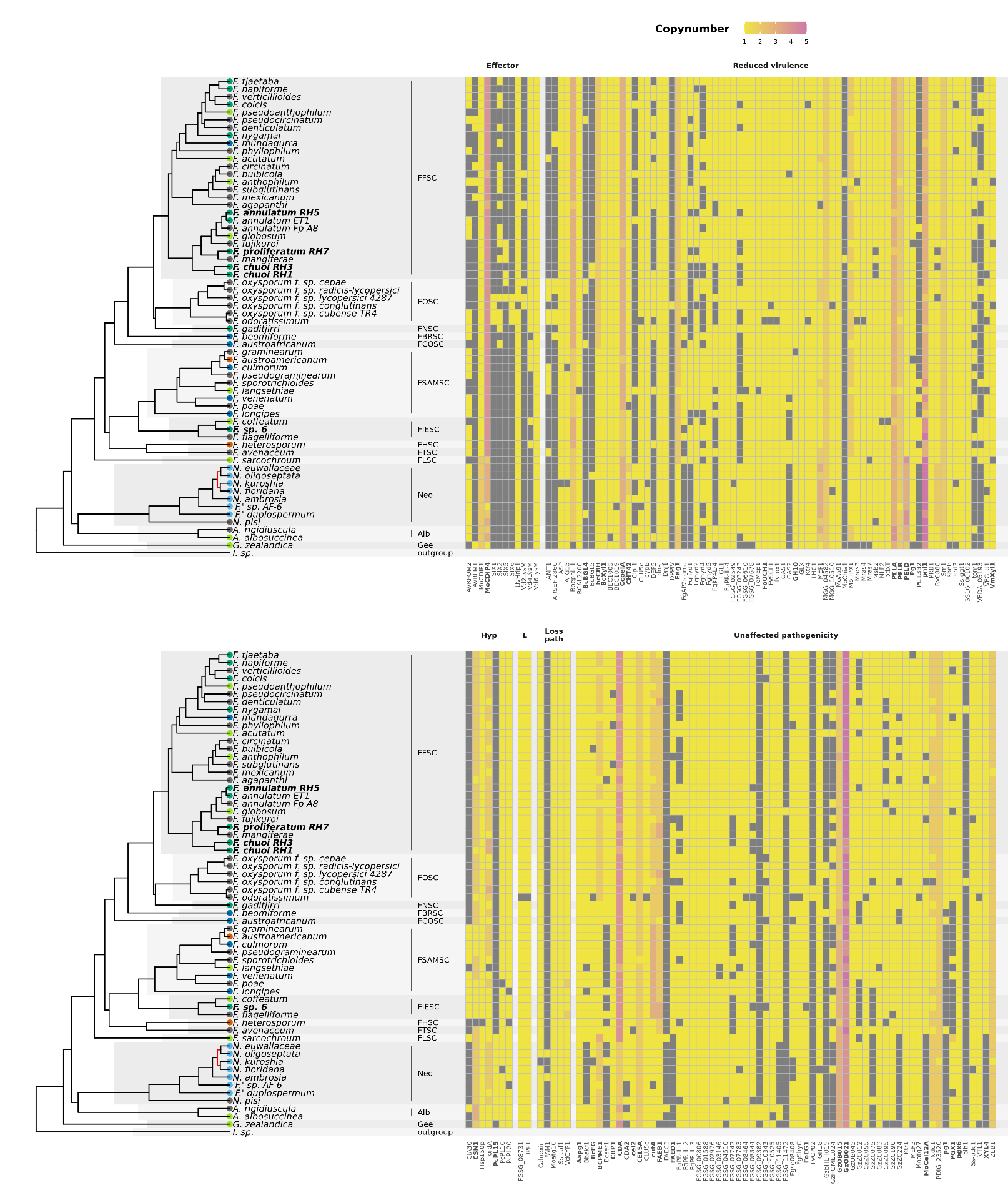


**Supplementary Figure 6.** Abundance matrix showing number of CSEPs in fusarioid taxa that could be matched to experimentally verified genes in PHI-base. Genes are grouped based on knockout mutant phenotypes curated in PHI-base (Hyp=hypervirulance, L=lethal, Loss path=loss of pathogenicity). Dark grey boxes indicate no CSEP for that taxon. Genes that are bold were also predicted to be CAZymes.


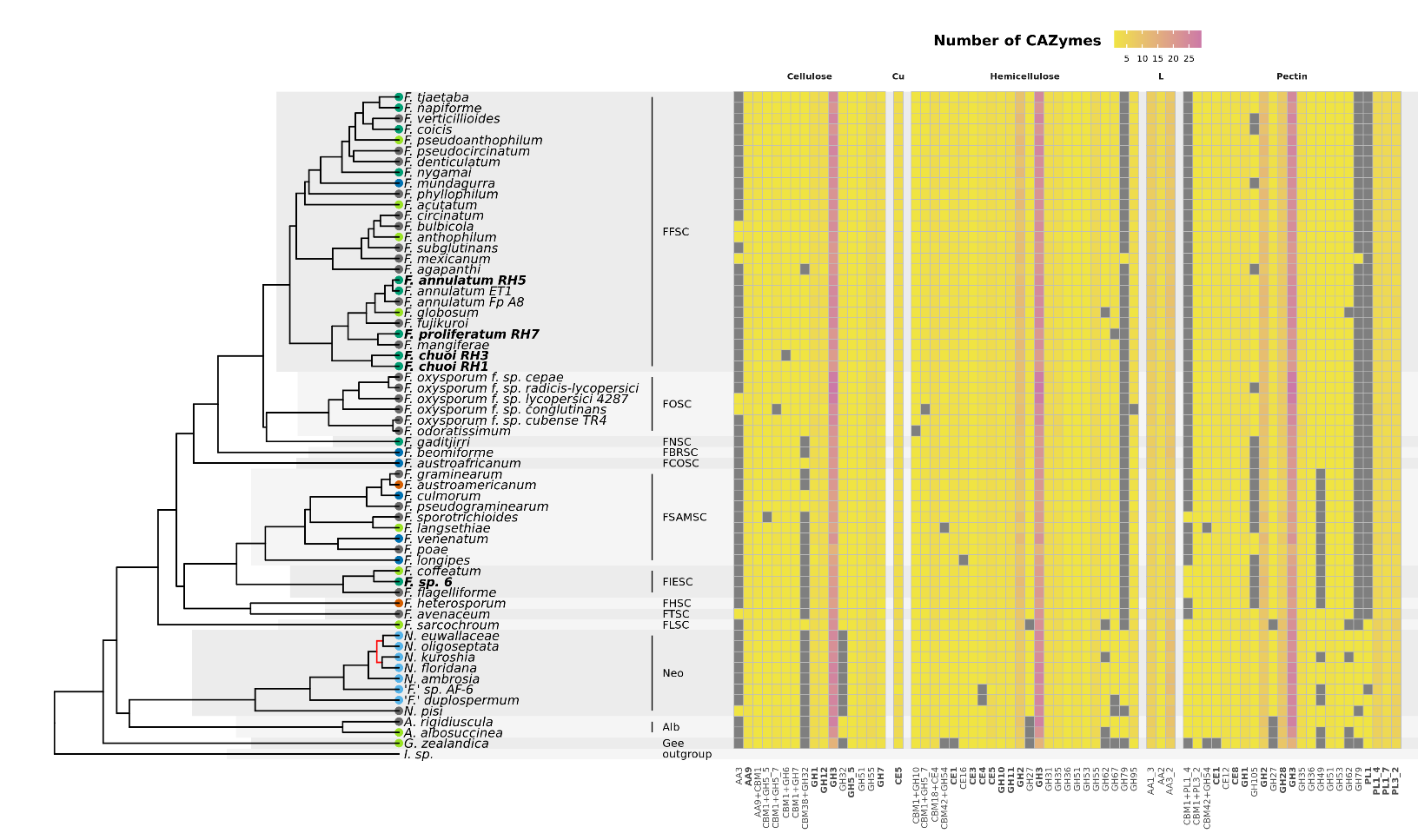
**Supplementary Figure 7.** Abundance matrix showing Number of CAZymes in fusarioid taxa belonging to families with known plant cell wall substrates (Cu=cutin, L=lignin). Dark grey boxes indicate no CAZyme family genes for that taxon. Genes that are bold were also predicted to be CSEPs.

| 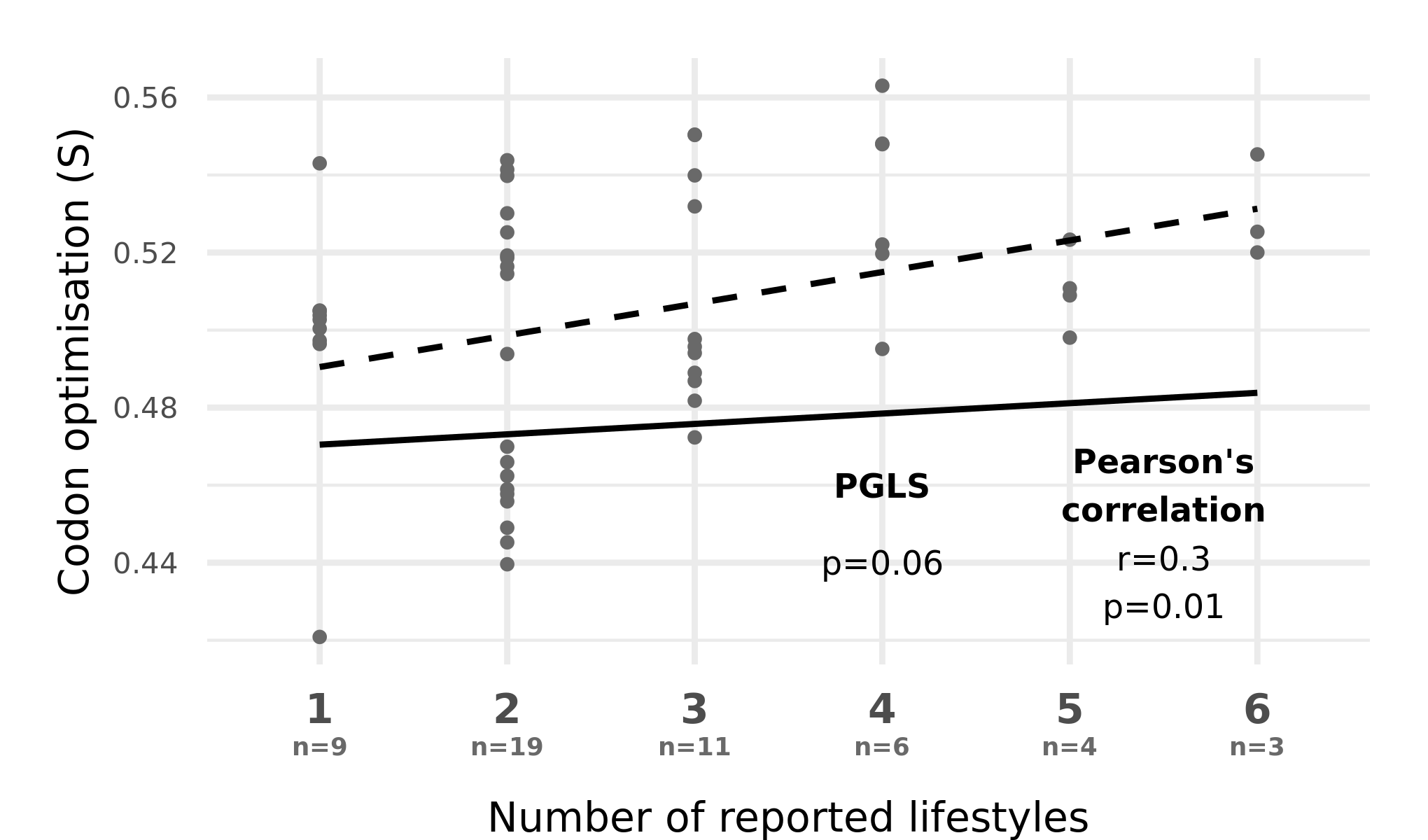 |
| --- |
| **Supplementary Figure 8.** Scatterplot showing the relationship between codon optimisation (S) of core single-copy genes and the number of reported lifestyles for species. The dashed line indicates the best fit of uncorrected data with a linear regression model (Pearson’s adj-R2=0.3, p=0.01), while the solid line indicates the phylogenetically corrected PGLS fit (p=0.06). Sample size (the number of species) is reported under x axis labels. |


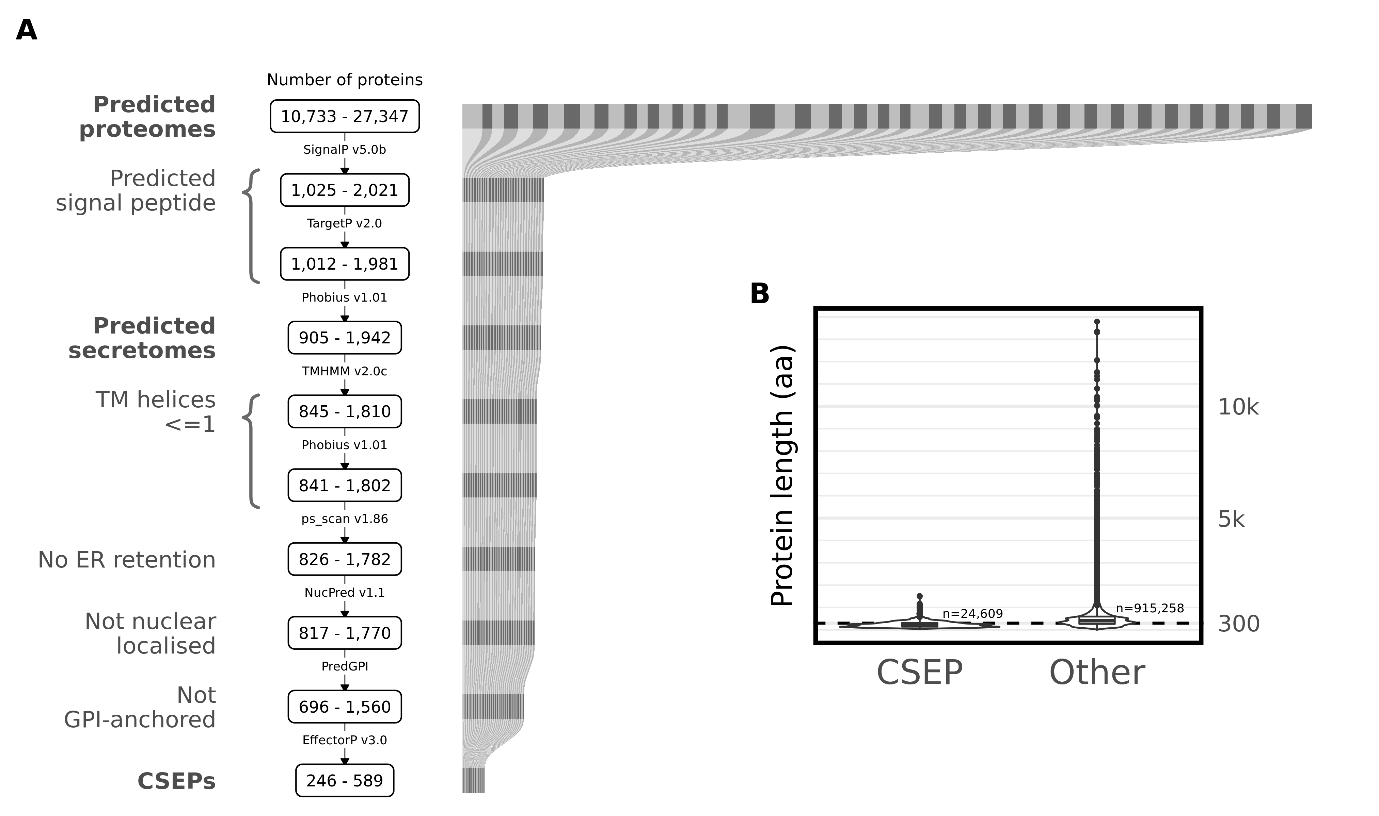


**Supplementary Figure 9.** (A) Alluvial plot indicating the number of proteins retained at each step of the computational CSEP prediction procedure, with different taxa indicated by alternating coloured boxes. The range of number of proteins across all taxa at each step is shown to the left of boxes. (B) The length of CSEPs following the prediction steps in comparison with all other proteins.


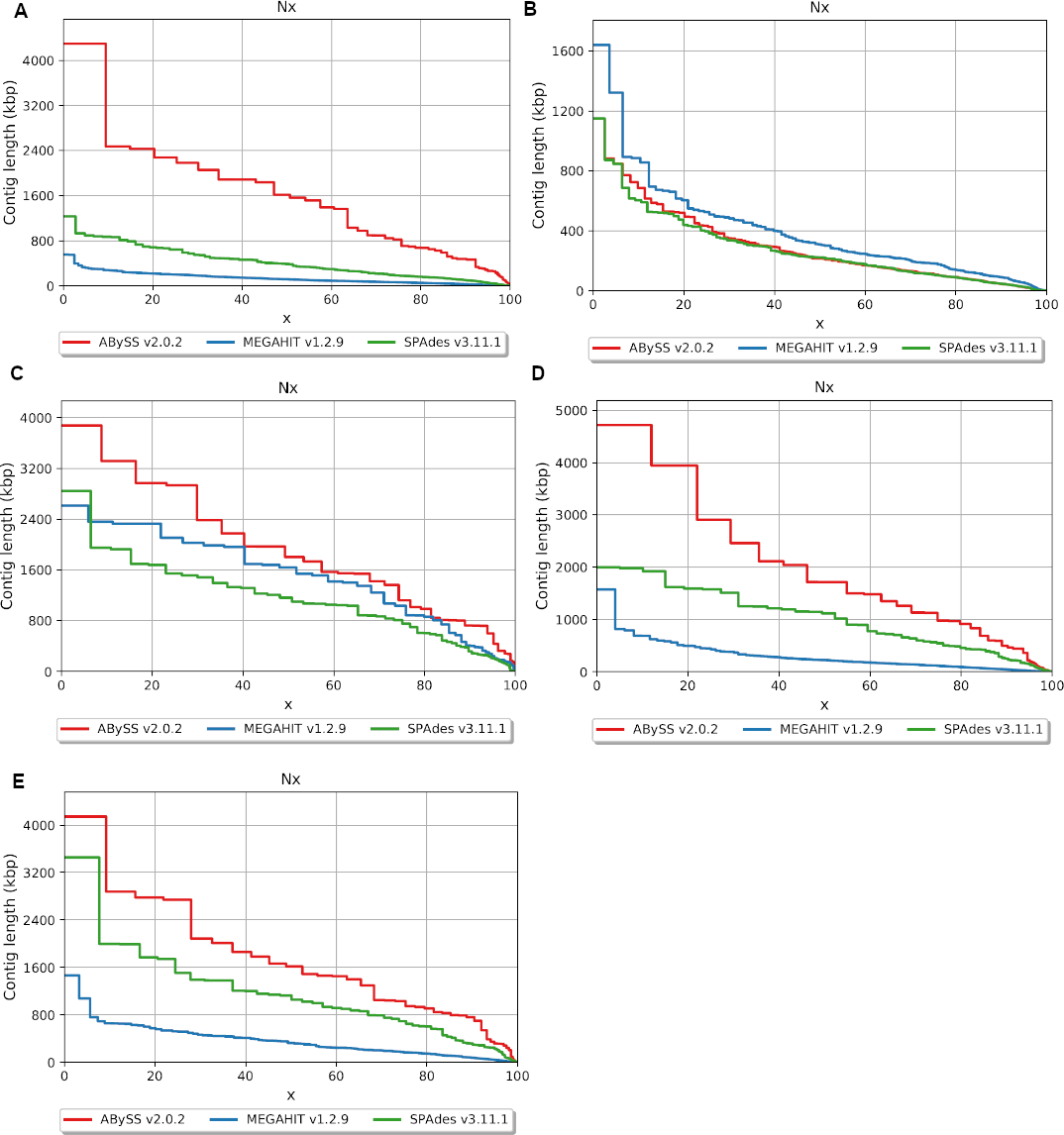


**Supplementary Figure 10.** Nx plots (the smallest contig length at which x% of the assembly is contained in contigs of at least that size) produced by QUAST for each of the strains sequenced in this study: (A) *F.* *chuoi* RH1 (B) *F. chuoi* RH3 (C) *F.* *annulatum* RH5 (D) *F.* sp. RH6 (E) *F.* *proliferatum* RH7.


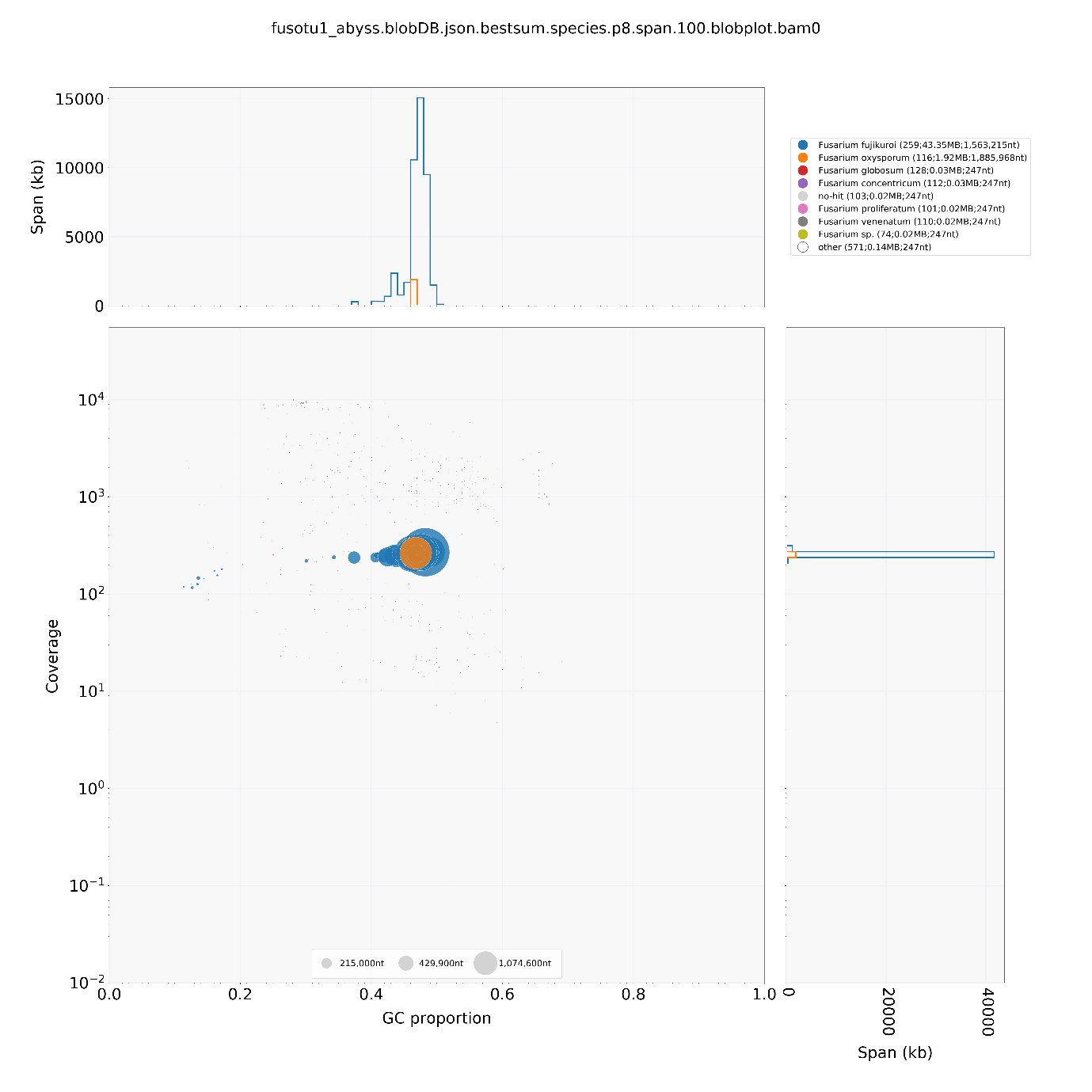


**A**


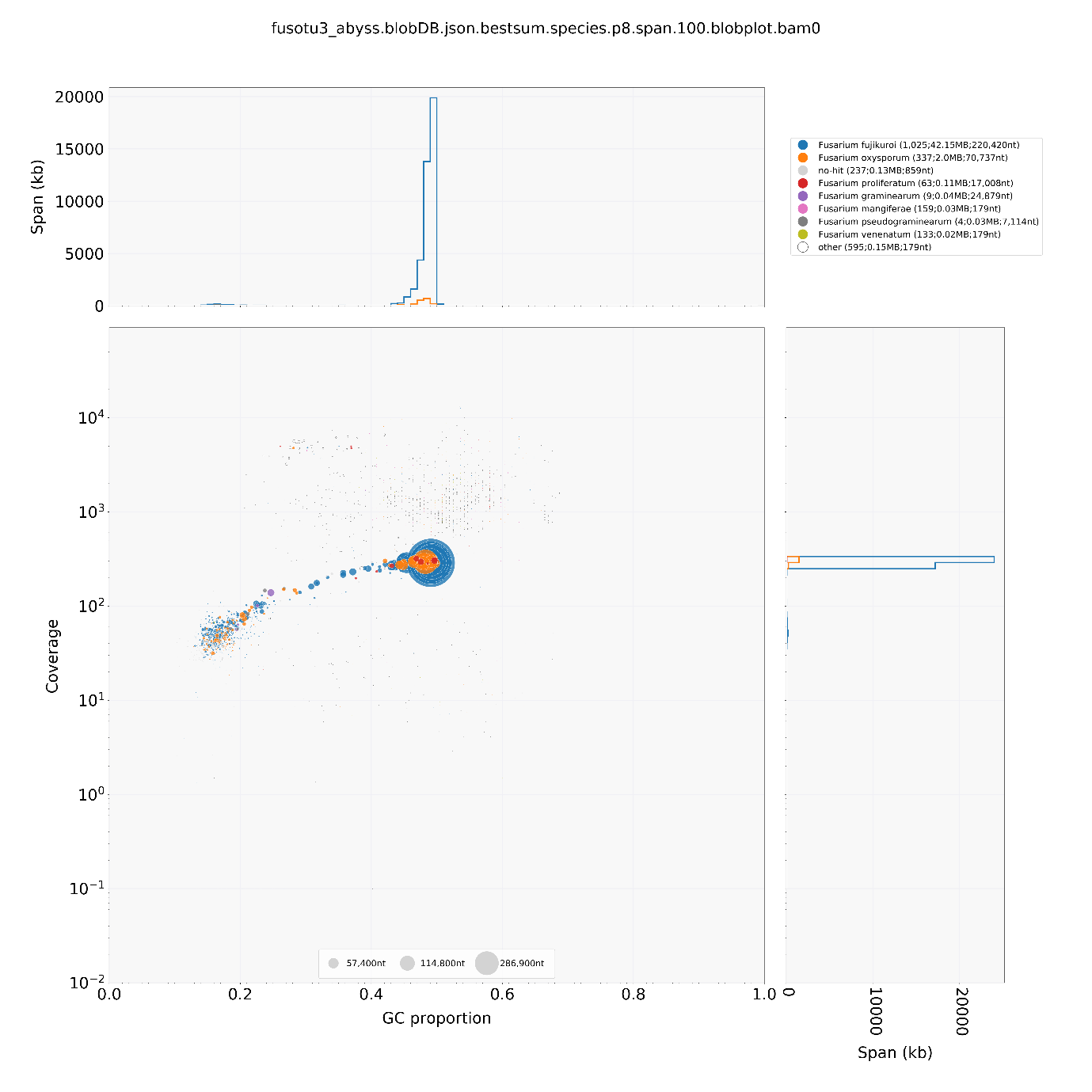


**B**


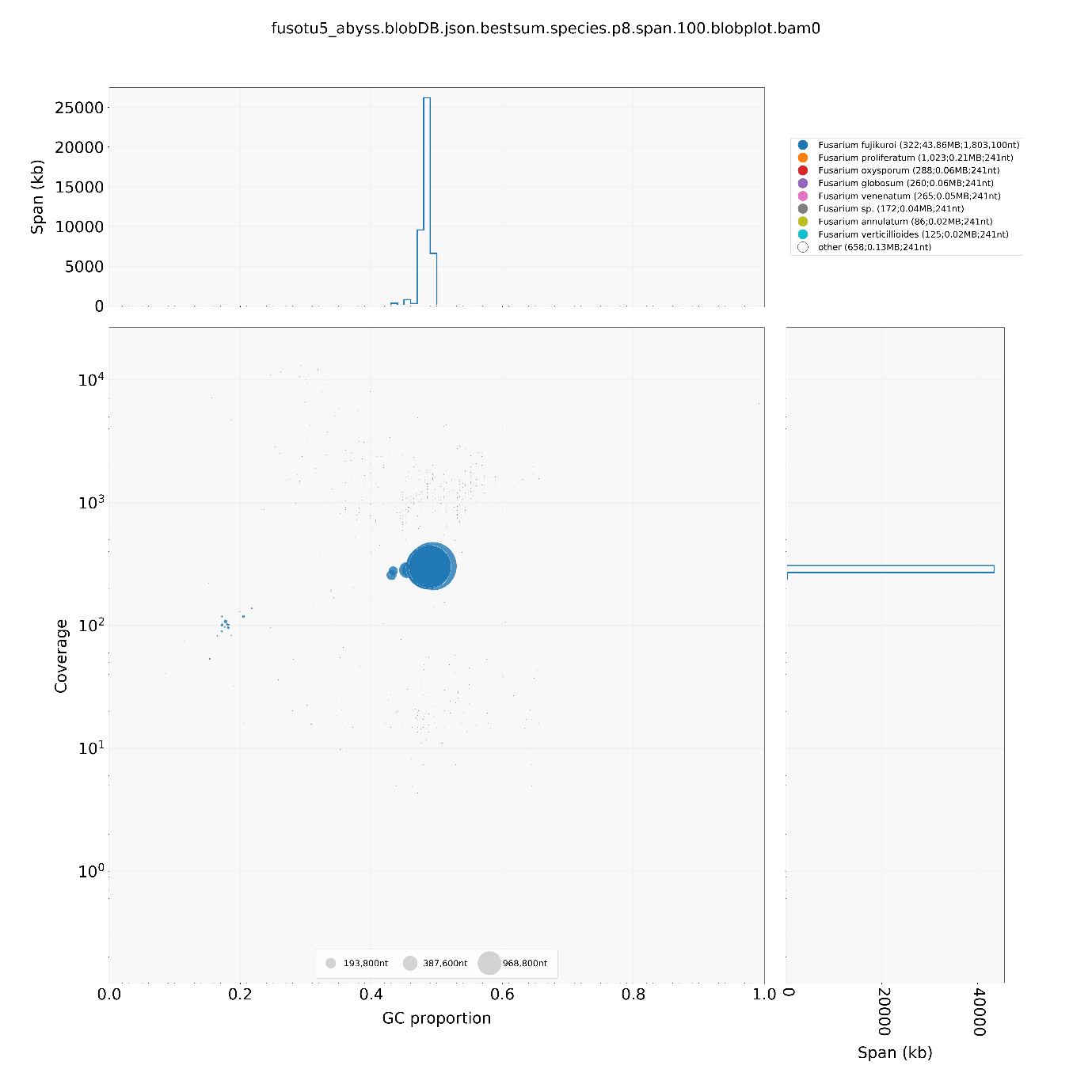


**C**


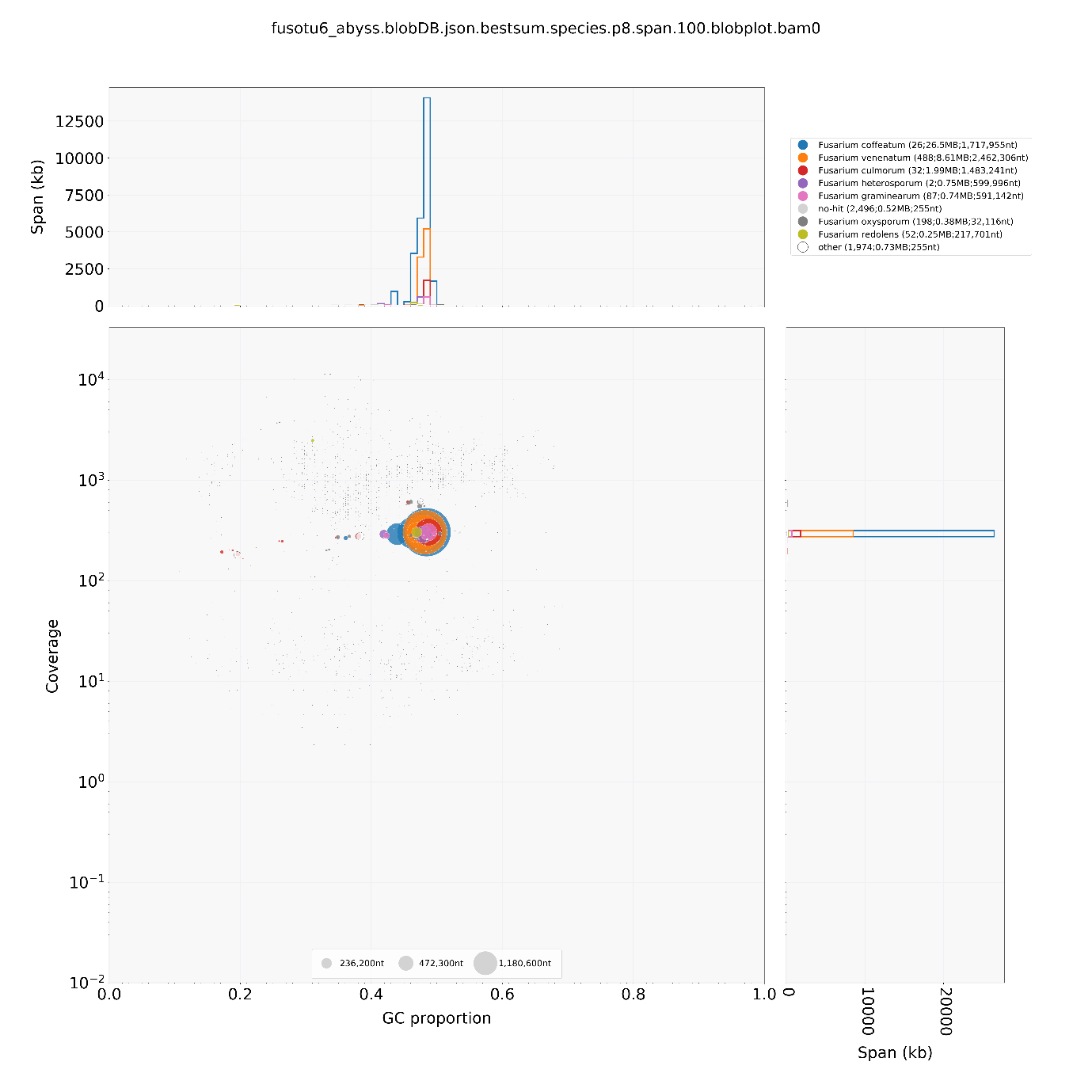


**D**


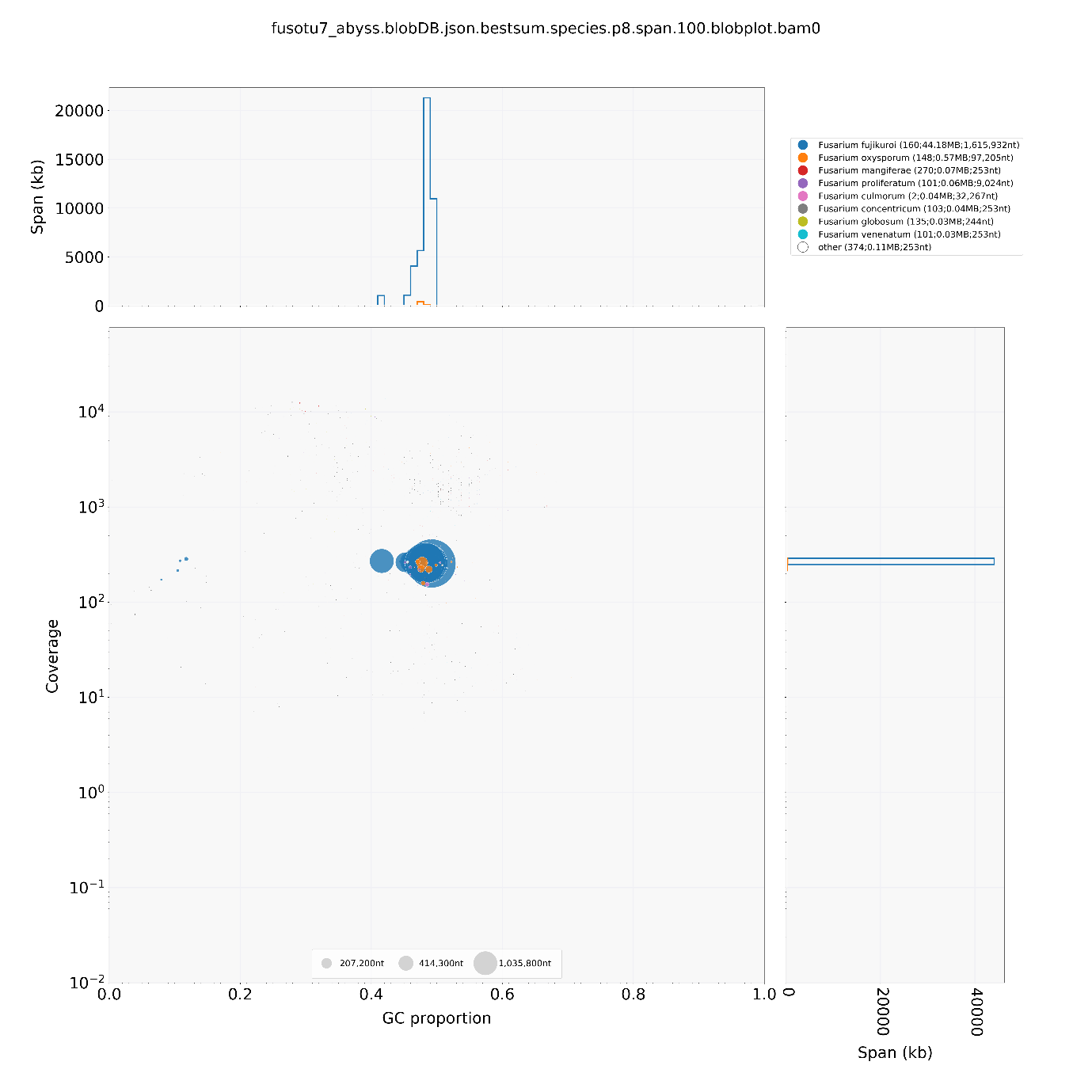


**E**

**Supplementary Figure 11**. BlobPlots showing the taxonomic classification of reads based on coverage and GC content: (A) *F.* *chuoi* RH1 (B) *F. chuoi* RH3 (C) *F.* *annulatum* RH5 (D) *F.* sp. RH6 (E) *F.* *proliferatum* RH7.


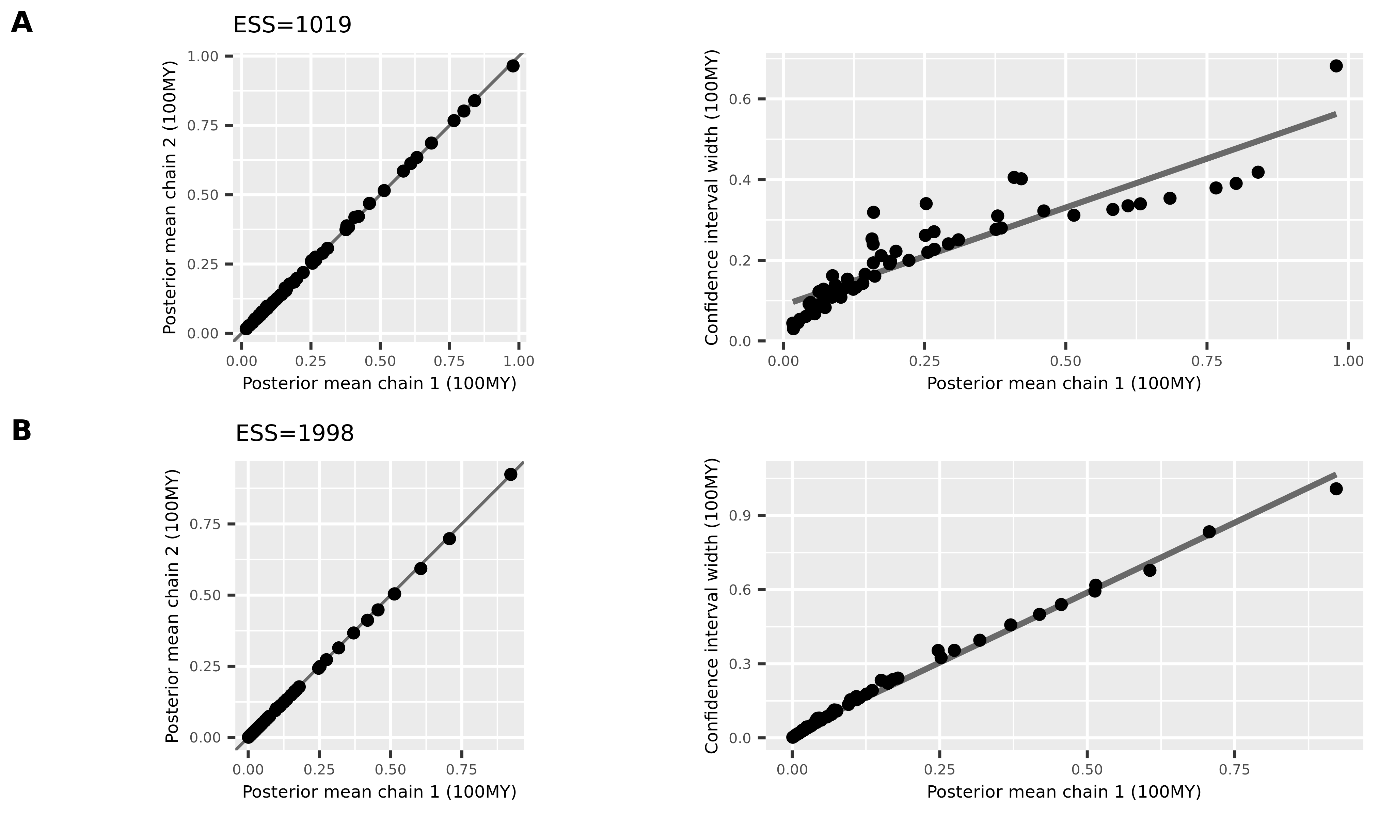


**Supplementary Figure 12.** Convergence of posterior means (left) and infinite-sites plot (right) from both MCMCTree chains for the AR clock model (A) and the IR clock model (B).

| 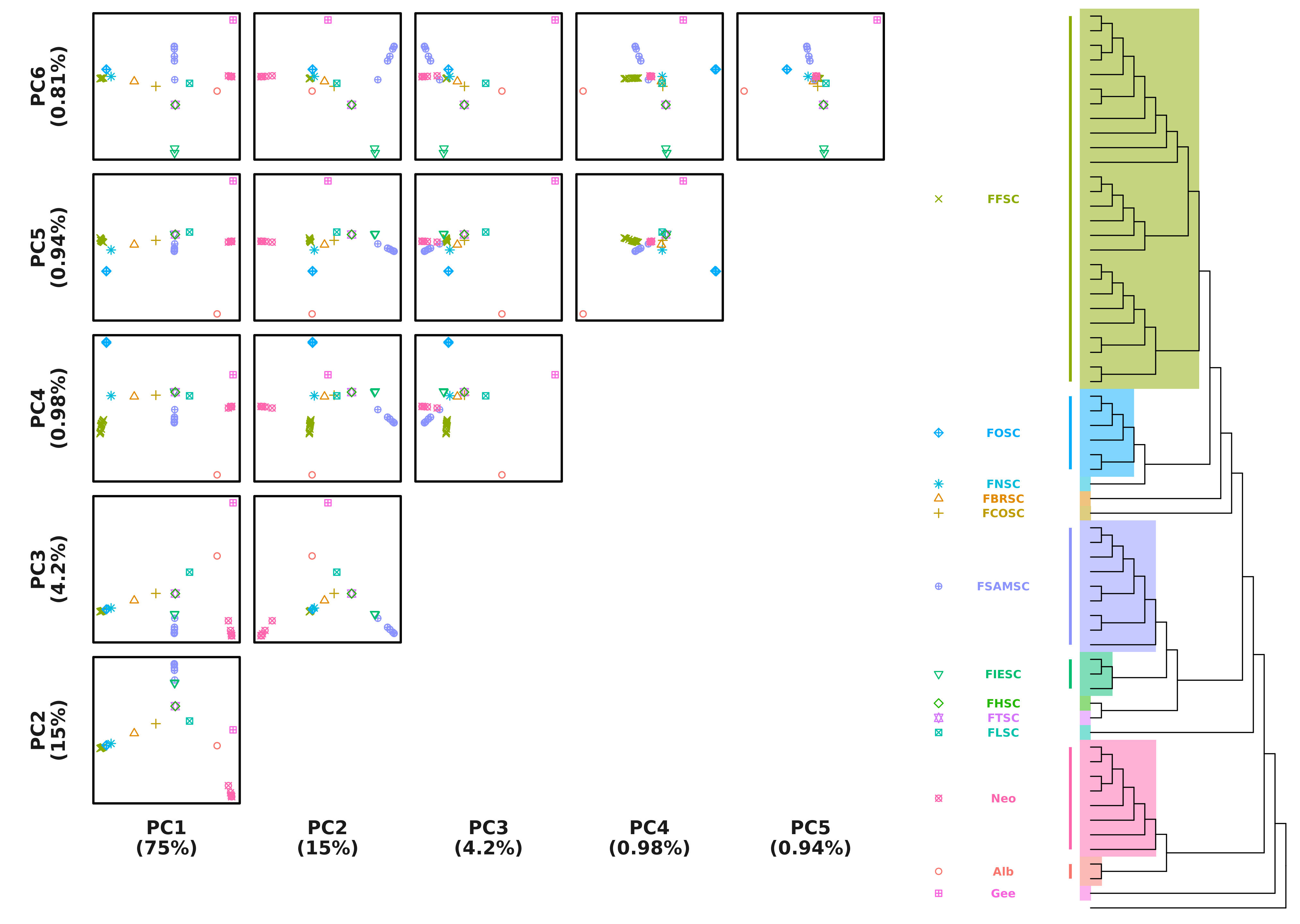 |
| --- |
| **Supplementary Figure 13.** PCAs of phylogenetic distances between taxa for the first 6 principal components, with points representing species complexes/allied genera, differentiated by shape and colour, as indicated by the tree legend. The percentage of variance explained by each principal component is shown on axis labels. |

**References**

1. Bolger, A. M., Lohse, M. & Usadel, B. Trimmomatic: a flexible trimmer for Illumina sequence data. *Bioinformatics* **30**, 2114–2120 (2014).

2. Andrews, S. FastQC: a quality control tool for high throughput sequence data. (2018). Available at: http://www.bioinformatics.babraham.ac.uk/projects/fastqc/.

3. Simpson, J. T. *et al.* ABySS: A parallel assembler for short read sequence data. *Genome Res.* **19**, 1117–1123 (2009).

4. Li, D. *et al.* MEGAHIT v1.0: A fast and scalable metagenome assembler driven by advanced methodologies and community practices. *Methods* **102**, 3–11 (2016).

5. Bankevich, A. *et al.* SPAdes: A New Genome Assembly Algorithm and Its Applications to Single-Cell Sequencing. *J. Comput. Biol.* **19**, 455–477 (2012).

6. Li, H. Aligning sequence reads, clone sequences and assembly contigs with BWA-MEM. (2013).

7. Walker, B. J. *et al.* Pilon: An integrated tool for comprehensive microbial variant detection and genome assembly improvement. *PLoS One* **9**, e112963 (2014).

8. Li, H. *et al.* The Sequence Alignment/Map format and SAMtools. *Bioinformatics* **25**, 2078–2079 (2009).

9. Gurevich, A., Saveliev, V., Vyahhi, N. & Tesler, G. QUAST: quality assessment tool for genome assemblies. *Bioinformatics* **29**, 1072–1075 (2013).

10. Simão, F. A., Waterhouse, R. M., Ioannidis, P., Kriventseva, E. V. & Zdobnov, E. M. BUSCO: assessing genome assembly and annotation completeness with single-copy orthologs. *Bioinformatics* **31**, 3210–3212 (2015).

11. Laetsch, D. R. & Blaxter, M. L. BlobTools: Interrogation of genome assemblies. *F1000Research* **6**, 1287 (2017).

12. Camacho, C. *et al.* BLAST+: architecture and applications. *BMC Bioinformatics* **10**, 421 (2009).

13. Quinlan, A. R. & Hall, I. M. BEDTools: a flexible suite of utilities for comparing genomic features. *Bioinformatics* **26**, 841–842 (2010).

14. Smit, A. & Hubley, R. RepeatModeler Open-1.0. (2015).

15. Smit, A., Hubley, R. & Green, P. RepeatMasker Open-4.0. (2015).

16. Cantarel, B. L. *et al.* MAKER: An easy-to-use annotation pipeline designed for emerging model organism genomes. *Genome Res.* **18**, 188–196 (2008).

17. Grigoriev, I. V *et al.* MycoCosm portal: gearing up for 1000 fungal genomes. *Nucleic Acids Res.* **42**, 699–704 (2014).

18. Mesny, F. *et al.* Genetic determinants of endophytism in the *Arabidopsis* root mycobiome. *Nat. Commun.* **12**, 7227 (2021).

19. Korf, I. Gene finding in novel genomes. *BMC Bioinformatics* **5**, 59 (2004).

20. Stanke, M. *et al.* AUGUSTUS: *ab initio* prediction of alternative transcripts. *Nucleic Acids Res.* **34**, 435–439 (2006).

21. Rangwala, S. H. *et al.* Accessing NCBI data using the NCBI sequence viewer and genome data viewer (GDV). *Genome Res.* **31**, 159–169 (2021).

22. Geib, S. M. *et al.* Genome Annotation Generator: a simple tool for generating and correcting WGS annotation tables for NCBI submission. *Gigascience* **7**, 1–5 (2018).

23. Jombart, T., Balloux, F. & Dray, S. adephylo: new tools for investigating the phylogenetic signal in biological traits. *Bioinformatics* **26**, 1907–1909 (2010).

24. R Core Team. R: A language and environment for statistical computing. (2020).

25. Beckerson, W. C. *et al.* Cause and Effectors: Whole-Genome Comparisons Reveal Shared but Rapidly Evolving Effector Sets among Host-Specific Plant-Castrating Fungi. *MBio* **10**, e02391-19 (2019).

26. Almagro Armenteros, J. J. *et al.* SignalP 5.0 improves signal peptide predictions using deep neural networks. *Nat. Biotechnol.* **37**, 420–423 (2019).

27. Almagro Armenteros, J. J. *et al.* Detecting sequence signals in targeting peptides using deep learning. *Life Sci. Alliance* **2**, e201900429 (2019).

28. Käll, L., Krogh, A. & Sonnhammer, E. L. L. A combined transmembrane topology and signal peptide prediction method. *J. Mol. Biol.* **338**, 1027–1036 (2004).

29. Krogh, A., Larsson, B., Von Heijne, G. & Sonnhammer, E. L. L. Predicting transmembrane protein topology with a hidden Markov model: Application to complete genomes. *J. Mol. Biol.* **305**, 567–580 (2001).

30. Gattiker, A., Gasteiger, E. & Bairoch, A. ScanProsite: a reference implementation of a PROSITE scanning tool. *Appl. Bioinformatics* **1**, 107–108 (2002).

31. Brameier, M., Krings, A. & MacCallum, R. M. NucPred—Predicting nuclear localization of proteins. *Bioinformatics* **23**, 1159–1160 (2007).

32. Pierleoni, A., Martelli, P. & Casadio, R. PredGPI: a GPI-anchor predictor. *BMC Bioinformatics* **9**, 392 (2008).

33. Dragićević, M. B., Paunović, D. M., Bogdanović, M. D., Todorović, S. I. & Simonović, A. D. ragp: Pipeline for mining of plant hydroxyproline-rich glycoproteins with implementation in R. *Glycobiology* **30**, 19–35 (2020).

34. Sperschneider, J. & Dodds, P. N. EffectorP 3.0: prediction of apoplastic and cytoplasmic effectors in fungi and oomycetes. *MPMI* (2021). doi:10.1094/MPMI-08-21-0201-R

35. Lo Presti, L. *et al.* Fungal Effectors and Plant Susceptibility. *Annu. Rev. Plant Biol.* **66**, 513–545 (2015).

36. Urban, M. *et al.* PHI-base: the pathogen-host interactions database. *Nucleic Acids Res.* **48**, D613–D620 (2020).

37. Zhang, H. *et al.* dbCAN2: a meta server for automated carbohydrate-active enzyme annotation. *Nucleic Acids Res.* **46**, W95–W101 (2018).

38. Mistry, J., Finn, R. D., Eddy, S. R., Bateman, A. & Punta, M. Challenges in homology search: HMMER3 and convergent evolution of coiled-coil regions. *Nucleic Acids Res.* **41**, e121 (2013).

39. Buchfink, B., Reuter, K. & Drost, H. G. Sensitive protein alignments at tree-of-life scale using DIAMOND. *Nat. Methods* **18**, 366–368 (2021).

40. Drula, E. *et al.* The carbohydrate-active enzyme database: functions and literature. *Nucleic Acids Res.* **50**, D571–D577 (2022).

41. Xu, J., Zhang, H., Zheng, J., Dovoedo, P. & Yin, Y. eCAMI: simultaneous classification and motif identification for enzyme annotation. *Bioinformatics* **36**, 2068–2075 (2020).

42. McDonald, A. G., Boyce, S. & Tipton, K. F. ExplorEnz: the primary source of the IUBMB enzyme list. *Nucleic Acids Res.* **37**, D593–D597 (2009).

43. Wickham, H. rvest: Easily Harvest (Scrape) Web Pages. (2020).

44. Zhao, Z., Liu, H., Wang, C. & Xu, J. R. Comparative analysis of fungal genomes reveals different plant cell wall degrading capacity in fungi. *BMC Genomics* **14**, (2013).

45. Miyauchi, S. *et al.* Large-scale genome sequencing of mycorrhizal fungi provides insights into the early evolution of symbiotic traits. *Nat. Commun.* **11**, 5125 (2020).

46. Hage, H. & Rosso, M. N. Evolution of Fungal Carbohydrate-Active Enzyme Portfolios and Adaptation to Plant Cell-Wall Polymers. *J. Fungi* **7**, 1 (2021).

47. Glass, N. L., Schmoll, M., Cate, J. H. D. & Coradetti, S. Plant cell wall deconstruction by ascomycete fungi. *Annu. Rev. Microbiol.* **67**, 477–498 (2013).

48. Levasseur, A., Drula, E., Lombard, V., Coutinho, P. M. & Henrissat, B. Expansion of the enzymatic repertoire of the CAZy database to integrate auxiliary redox enzymes. *Biotechnol. Biofuels* **6**, 41 (2013).

49. Mesny, F. & Vannier, N. Detecting the effect of biological categories on genome composition. (2020).

50. Pedregosa, F. *et al.* Scikit-learn: Machine Learning in Python. *J. Mach. Learn. Res.* **12**, 2825–2830 (2011).

51. Oksanen, J. *et al.* vegan: Community Ecology Package. (2019).

52. Hervé, M. RVAideMemoire: Testing and Plotting Procedures for Biostatistics. (2020).

53. Kassambara, A. ggpubr: ‘ggplot2’ Based Publication Ready Plots. (2020).

54. Kassambara, A. rstatix: Pipe-Friendly Framework for Basic Statistical Tests. (2021).

55. Villacorta, P. J. ART: Aligned Rank Transform for Nonparametric Factorial Analysis. (2015).

56. Sauder, D. C. & DeMars, C. E. An Updated Recommendation for Multiple Comparisons. *Adv. Methods Pract. Psychol. Sci.* **2**, 26–44 (2019).

57. Larsson, A. AliView: a fast and lightweight alignment viewer and editor for large datasets. *Bioinformatics* **30**, 3276 (2014).

58. Suyama, M., Torrents, D. & Bork, P. PAL2NAL: robust conversion of protein sequence alignments into the corresponding codon alignments. *Nucleic Acids Res.* **34**, W609–W612 (2006).

59. Murrell, B. *et al.* Gene-wide Identification of Episodic Selection. *Mol. Biol. Evol.* **32**, 1365–1371 (2015).

60. Smith, M. D. *et al.* Less Is More: An Adaptive Branch-Site Random Effects Model for Efficient Detection of Episodic Diversifying Selection. *Mol. Biol. Evol.* **32**, 1342–1353 (2015).

61. Kosakovsky Pond, S. L., Wisotsky, S. R., Escalante, A., Magalis, B. R. & Weaver, S. Contrast-FEL—A Test for Differences in Selective Pressures at Individual Sites among Clades and Sets of Branches. *Mol. Biol. Evol.* **38**, 1184–1198 (2021).

62. Sharp, P. M. & Li, W.-H. The codon adaptation index - a measure of directional synonymous codon usage bias, and its potential applications. *Nucleic Acids Res.* **15**, 1281–1295 (1987).

63. Cuomo, C. A. *et al.* The *Fusarium graminearum* Genome Reveals a Link Between Localized Polymorphism and Pathogen Specialization. *Science.* **317**, 1400–1403 (2007).

64. Elek, A., Kuzman, M. & Vlahovicek, K. coRdon: Codon Usage Analysis and Prediction of Gene Expressivity. (2021).

65. Charif, D. & Lobry, J. R. SeqinR 1.0-2: A Contributed Package to the R Project for Statistical Computing Devoted to Biological Sequences Retrieval and Analysis. in *Structural approaches to sequence evolution: Molecules, networks, populations* (eds. Bastolla, U., Porto, M., Roman, H. E. & Vendruscolo, M.) 207–232 (Springer, 2007). doi:10.1007/978-3-540-35306-5_10

66. dos Reis, M., Savva, R. & Wernisch, L. Solving the riddle of codon usage preferences: A test for translational selection. *Nucleic Acids Res.* **32**, 5036–5044 (2004).

67. Pinheiro, J., Bates, D., DebRoy, S., Sarkar, D. & Team, R. C. nlme: Linear and Nonlinear Mixed Effects Models. (2021).

68. Paradis, E. & Schliep, K. Ape 5.0: An environment for modern phylogenetics and evolutionary analyses in R. *Bioinformatics* **35**, 526–528 (2019).

69. Wilke, C. O. cowplot: Streamlined Plot Theme and Plot Annotations for ‘ggplot2’. (2020).

70. Gearty, W. deeptime: Plotting Tools for Anyone Working in Deep Time. (2021).

71. Galili, T. dendextend: An R package for visualizing, adjusting and comparing trees of hierarchical clustering. *Bioinformatics* **31**, 3718–3720 (2015).

72. Wickham, H., François, R., Henry, L. & Müller, K. dplyr: A Grammar of Data Manipulation. (2020).

73. Larsson, J. eulerr: Area-Proportional Euler and Venn Diagrams with Ellipses. (2020).

74. Wickham, H. ggplot2: Elegant Graphics for Data Analysis. (2016).

75. Brunson, J. ggalluvial: Layered Grammar for Alluvial Plots. *J. Open Source Softw.* **5**, 2017 (2020).

76. Pedersen, T. L. ggforce: Accelerating ‘ggplot2’. (2021).

77. Campitelli, E. ggnewscale: Multiple Fill and Colour Scales in ‘ggplot2’. (2020).

78. Yu, G. ggplotify: Convert Plot to ‘grob’ or ‘ggplot’ Object. (2021).

79. Slowikowski, K. ggrepel: Automatically Position Non-Overlapping Text Labels with ‘ggplot2’. (2020).

80. Arnold, J. B. ggthemes: Extra Themes, Scales and Geoms for ‘ggplot2’. (2021).

81. Yu, G., Smith, D. K., Zhu, H., Guan, Y. & Lam, T. T. Y. GGTREE: an R package for visualization and annotation of phylogenetic trees with their covariates and other associated data. *Methods Ecol. Evol.* **8**, 28–36 (2017).

82. Ooms, J. The jsonlite Package: A Practical and Consistent Mapping Between JSON Data and R Objects. *arXiv* 1403.2805v1 (2014).

83. Bengtsson, H. matrixStats: Functions that Apply to Rows and Columns of Matrices (and to Vectors). (2021).

84. Puttick, M. & Title, P. MCMCtreeR: Prepare MCMCtree Analyses and Plot Bayesian Divergence Time Analyses Estimates on Trees. (2019).

85. Campitelli, E. metR: Tools for Easier Analysis of Meteorological Fields. (2021). doi:10.5281/zenodo.2593516

86. Graves, S., Piepho, H.-P., Selzer, L. & Dorai-Raj, S. multcompView: Visualizations of Paired Comparisons. (2019).

87. Schulz, A. pBrackets: Plot Brackets. (2021).

88. Revell, L. J. phytools: an R package for phylogenetic comparative biology (and other things). *Methods Ecol. Evol.* **3**, 217–223 (2012).

89. Wickham, H. The split-apply-combine strategy for data analysis. *J. Stat. Softw.* **40**, 1–29 (2011).

90. Wickham, H. Reshaping Data with the reshape Package. *J. Stat. Softw.* **21**, 1–20 (2007).

91. Wickham, H. & Seidel, D. scales: Scale Functions for Visualization. (2020).

92. Gagolewski, M. & Tartanus, B. stringi: Character String Processing Facilities. (2021).

93. Wickham, H. stringr: Simple, Consistent Wrappers for Common String Operations. (2019).

94. Wickham, H. & Girlich, M. tidyr: Tidy Messy Data. (2022).

95. RStudio Team. RStudio: Integrated Development for R. (2015).

96. Butcher, S., King, T. & Zalewski, L. Apocrita - High Performance Computing Cluster for Queen Mary University of London. (2017). doi:10.5281/zenodo.438045

97. Geiser, D. M. *et al.* FUSARIUM-ID v. 1.0: A DNA sequence database for identifying *Fusarium*. *Eur. J. Plant Pathol.* **110**, 473–479 (2004).

98. NCBI. *Fusarium albosuccineum* NRRL 20459. Available at: https://www.ncbi.nlm.nih.gov/biosample/SAMN13683636/. (Accessed: 17th May 2021)

99. Nelson, A., Vandegrift, R., Carroll, G. C. & Roy, B. A. Double lives: transfer of fungal endophytes from leaves to woody substrates. *PeerJ* **8**, e9341 (2020).

100. ARS Culture Collection. *Fusarium decemcellulare* NRRL 13412. Available at: https://nrrl.ncaur.usda.gov/cgi-bin/usda/mold/report.html?nrrlcodes=13412. (Accessed: 25th March 2021)

101. Harry, C., Keith, A. & Sarah, E. Endophytes and mycoparasites associated with an indigenous forest tree, *Theobroma gileri*, in Ecuador and a preliminary assessment of their potential as biocontrolagents of cocoa diseases. *Mycol. Prog.* **2**, 149–160 (2003).

102. Serrato-Diaz, L. M., Perez-Cuevas, M., Rivera-Vargas, L. I., Goenaga, R. & French-Monar, R. D. First Report of *Fusarium decemcellulare* Causing Inflorescence Wilt and Vascular and Flower Necrosis of Rambutan (*Nephelium lappaceum*), Longan (*Dimocarpus longan*), and Mango (*Mangifera indica*). *Plant Dis.* **99**, (2015).

103. Lee, S. *et al.* First Report of Fruit Rot Caused by *Fusarium decemcellulare* in Apples in Korea. *Korean J. Mycol.* **45**, 54–62 (2017).

104. Wang, Y. X., Chen, J. Y., Li, D. W., Huang, J. B. & Zheng, L. First Report of Canker of *Magnolia denudata* Caused by *Fusarium decemcellulare* in Hubei, China. *Plant Dis.* **99**, (2015).

105. Darvas, J. & Kotze, J. Fungi associated with pre-and postharvest diseases of avocado fruit at Westfalia Estate, South Africa. *Phytophylactica* **19**, 83–85 (1987).

106. Nirenberg, H. I. & O’Donnell, K. New *Fusarium* species and combinations within the *Gibberella fujikuroi* species complex. *Mycologia* **90**, 434–458 (1998).

107. Taj-Aldeen, S. J. *et al.* Gangrenous necrosis of the diabetic foot caused by *Fusarium acutatum*. *Med. Mycol.* **44**, 547–552 (2006).

108. Gupta, C. *et al.* Genotyping and *In Vitro* Antifungal Susceptibility Testing of *Fusarium* Isolates from Onychomycosis in India. *Mycopathologia* **181**, 497–504 (2016).

109. Muraosa, Y. *et al.* Epidemiological study of *Fusarium* species causing invasive and superficial fusariosis in Japan. *Med. Mycol. J.* **58E**, E5–E13 (2017).

110. Edwards, J. *et al.* *Fusarium agapanthi* sp. nov., a novel bikaverin and fusarubin-producing leaf and stem spot pathogen of *Agapanthus praecox* (African lily) from Australia and Italy. *Mycologia* **108**, 981–992 (2016).

111. Guarnaccia, V., Aiello, D., Polizzi, G., Crous, P. W. & Sandoval-Denis, M. Soilborne diseases caused by *Fusarium* and *Neocosmospora* spp. on ornamental plants in Italy. *Phytopathol. Mediterr.* **58**, 127–137 (2019).

112. Niehaus, E. M. *et al.* Comparative ‘Omics’ of the *Fusarium fujikuroi* Species Complex Highlights Differences in Genetic Potential and Metabolite Synthesis. *Genome Biol. Evol.* **8**, 3574–3599 (2016).

113. Romón, P. *et al.* Fungal communities associated with pitch canker disease of *Pinus radiata* caused by *Fusarium circinatum* in northern Spain: association with insects and pathogen-saprophyte antagonistic interactions. *Can. J. Plant Pathol.* **30**, 241–253 (2008).

114. Bentley, A. R., Petrovic, T., Griffiths, S. P., Burgess, L. W. & Summerell, B. A. Crop pathogens and other *Fusarium* species associated with *Austrostipa aristiglumis*. *Australas. Plant Pathol.* **36**, 434–438 (2007).

115. Akgül, D. S. & Ahioğlu, M. Fungal pathogens associated with young grapevine decline in the Southern Turkey vineyards. *BIO Web Conf.* **15**, 01027 (2019).

116. Gazis, R. & Chaverri, P. Diversity of fungal endophytes in leaves and stems of wild rubber trees (Hevea brasiliensis) in Peru. *Fungal Ecol.* **3**, 240–254 (2010).

117. Rajmohan, N., Gianfagna, T. J., Meca, G., Moretti, A. & Zhang, N. Molecular identification and mycotoxin production of *Lilium longiflorum*-associated fusaria isolated from two geographic locations in the United States. *Eur. J. Plant Pathol.* **131**, 631–642 (2011).

118. O’Donnell, K. *et al.* Phylogenetic diversity and microsphere array-based genotyping of human pathogenic fusaria, including isolates from the multistate contact lens-associated U.S. keratitis outbreaks of 2005 and 2006. *J. Clin. Microbiol.* **45**, 2235–2248 (2007).

119. Torbati, M., Arzanlou, M., Sandoval-Denis, M. & Crous, P. W. Multigene phylogeny reveals new fungicolous species in the *Fusarium tricinctum* species complex and novel hosts in the genus *Fusarium* from Iran. *Mycol. Prog.* **18**, 119–133 (2019).

120. Wang, C.-Y. *et al.* First Report of Silk Tree (*Albizia julibrissin*) Wilt Caused by *Fusarium proliferatum* in Anhui Province of China. *Plant Dis.* **103**, (2019).

121. Carrieri, R., Raimo, F., Pentangelo, A. & Lahoz, E. *Fusarium proliferatum* and *Fusarium tricinctum* as causal agents of pink rot of onion bulbs and the effect of soil solarization combined with compost amendment in controlling their infections in field. *Crop Prot.* **43**, 31–37 (2013).

122. Quesada-Ocampo, L. M., Butler, S., Withers, S. & Ivors, K. First Report of Fusarium Rot of Garlic Bulbs Caused by *Fusarium proliferatum* in North Carolina. *Plant Dis.* **98**, (2014).

123. Yamazaki, M., Morita, Y., Kashiwa, T., Teraoka, T. & Arie, T. *Fusarium proliferatum*, an additional bulb rot pathogen of Chinese chive. *J. Gen. Plant Pathol.* **79**, 431–434 (2013).

124. Punja, Z. K. First report of *Fusarium proliferatum* causing crown and stem rot, and pith necrosis, in cannabis (*Cannabis sativa* L., marijuana) plants. *Can. J. Plant Pathol.* **43**, 236–255 (2021).

125. Kim, S. G. *et al.* First Report of Fusarium Wilt Caused by *Fusarium proliferatum* on Safflower. *Res. Plant Dis.* **22**, 111–115 (2016).

126. Habibi, A. & Safaiefarahani, B. First Report of Fusarium Wilt of *Colchicum kotschyi* Caused by *Fusarium proliferatum* in Iran. *Plant Dis.* **103**, (2019).

127. Liu, N. *et al.* First Report of *Fusarium proliferatum* Causing Crown and Stem Rot of *Echeveria desmetiana* in China. *Plant Dis.* **104**, 3260 (2020).

128. Al Mahmooli, I. H., Al Balushi, F., Doyle, O., Al Sadi, A. M. & Deadman, M. L. First Report of Gladiolus Corm Rot Caused by *Fusarium proliferatum* in Oman. *Plant Dis.* **97**, (2013).

129. Lee, H. B. *et al.* First Report of Crown Rot on Gypsophila (*Gypsophila paniculata*) Caused by *Fusarium proliferatum* in Korea. *Plant Dis.* **95**, (2011).

130. Feng, F. S. & Li, H. First Report of *Fusarium proliferatum* Causing Trunk Canker on *Ilex cornuta* in China. *Plant Dis.* **103**, (2019).

131. Almanza-Álvarez, J. *et al.* Identification and control of pathogenic fungi in neotropical valued orchids (*Laelia* spp.). *Trop. Plant Pathol.* **42**, 339–351 (2017).

132. Cheng, Y. *et al.* *Fusarium* species in declining wild apple forests on the northern slope of the Tian Shan Mountains in north-western China. *For. Pathol.* **49**, e12542 (2019).

133. Huang, S. P. *et al.* First report of sheath rot caused by *Fusarium proliferatum* on Pisang Awak Banana (*Musa* ABB) in China. *J. Plant Pathol.* **101**, 1271–1272 (2019).

134. Choi, H. W., Hong, S. K., Lee, Y. K., Kim, W. G. & Chun, S. Taxonomy of *Fusarium fujikuroi* species complex associated with bakanae on rice in Korea. *Australas. Plant Pathol.* **47**, 23–34 (2018).

135. Zhou, X., Rao, B., Chen, Y. & Cai, C. First report of leaf blight caused by *Fusarium proliferatum* on *Polygonatum cyrtonema* in China. *J. Plant Pathol.* **103**, 369 (2021).

136. Xie, S. H. *et al.* *Fusarium proliferatum*: A New Pathogen Causing Fruit Rot of Peach in Ningde, China. *Plant Dis.* **102**, (2018).

137. Kee, Y. J., Zakaria, L. & Mohd, M. H. Morphology, phylogeny and pathogenicity of *Fusarium* species from *Sansevieria trifasciata* in Malaysia. *Plant Pathol.* **69**, 442–454 (2020).

138. Ren, J. *et al.* First Report of Sunflower Wilt Caused by *Fusarium proliferatum* in Inner Mongolia, China. *Plant Dis.* **99**, (2015).

139. Pérez, B. A., Berretta, M. F., Carrión, E. & Wright, E. R. First Report of Root Rot Caused by *Fusarium proliferatum* on Blueberry in Argentina. *Plant Dis.* **95**, (2011).

140. Park, M. S., Oh, S. Y., Lee, S., Eimes, J. A. & Lim, Y. W. Fungal diversity and enzyme activity associated with sailfin sandfish egg masses in Korea. *Fungal Ecol.* **34**, 1–9 (2018).

141. Ding, S., Hu, H. & Gu, J. D. Fungi colonizing wood sticks of Chinese fir incubated in subtropical urban soil growing with *Ficus microcarpa* trees. *Int. J. Environ. Sci. Technol.* **12**, 3781–3790 (2015).

142. Mohammadian, E., Arzanlou, M. & Babai-Ahari, A. Diversity of culturable fungi inhabiting petroleum-contaminated soils in Southern Iran. *Antonie Van Leeuwenhoek* **110**, 903–923 (2017).

143. Tischner, Z. *et al.* Environmental characteristics and taxonomy of microscopic fungi isolated from washing machines. *Fungal Biol.* **123**, 650–659 (2019).

144. NCBI. *Fusarium proliferatum* FpA8. Available at: https://www.ncbi.nlm.nih.gov/biosample/SAMN05529104/. (Accessed: 25th March 2021)

145. Hill, R. *et al.* Seed Banks as Incidental Fungi Banks: Fungal Endophyte Diversity in Stored Seeds of Banana Wild Relatives. *Front. Microbiol.* **12**, 643731 (2021).

146. NCBI. *Fusarium anthophilum* NRRL 25214. Available at: https://www.ncbi.nlm.nih.gov/biosample/SAMN13683615/. (Accessed: 1st September 2021)

147. Rodrigues, A. A. C. & Menezes, M. Identiﬁcation and pathogenic characterization of endophytic *Fusarium* species from cowpea seeds. *Mycopathologia* **159**, 79–85 (2005).

148. Akanmu, A. O., Abiala, M. A. & Odebode, A. C. Pathogenic Effect of Soilborne *Fusarium* Species on the Growth of Millet Seedlings. *World J. Agric. Sci.* **9**, 60–68 (2013).

149. Sharfun-Nahar & Mushtaq, M. Pathogenecity and transmission studies of seed-borne *Fusarium* species (sec. Liseola and Sporotrichiella) in sunflower. *Pakistan J. Bot.* **38**, 487–492 (2006).

150. Jacobs-Venter, A. *et al.* Molecular systematics of two sister clades, the *Fusarium concolor* and *F. babinda* species complexes, and the discovery of a novel microcycle macroconidium–producing species from South Africa. *Mycologia* **110**, 1189–1204 (2018).

151. NCBI. *Fusarium austroamericanum* NRRL 2903. Available at: https://www.ncbi.nlm.nih.gov/biosample/SAMN13683584. (Accessed: 21st May 2021)

152. Romero Cortes, T., López Pérez, P. A., Ramírez-Lepe, M. & Cuervo-Parra, J. . Aislamiento y caracterización morfológica de diferentes especies de hongos aislados de semillas de plantas de cebada (*Hordeum vulgare*). *Rev. Divulg. Técnica Agrícola y Agroindustrial* **65**, 1–10 (2016).

153. Umpiérrez-Failache, M. *et al.* Regional differences in species composition and toxigenic potential among Fusarium head blight isolates from Uruguay indicate a risk of nivalenol contamination in new wheat production areas. *Int. J. Food Microbiol.* **166**, 135–140 (2013).

154. Tralamazza, S. M., Bemvenuti, R. H., Zorzete, P., de Souza Garcia, F. & Corrêa, B. Fungal diversity and natural occurrence of deoxynivalenol and zearalenone in freshly harvested wheat grains from Brazil. *Food Chem.* **196**, 445–450 (2016).

155. Kokkonen, M., Ojala, L., Parikka, P. & Jestoi, M. Mycotoxin production of selected *Fusarium* species at different culture conditions. *Int. J. Food Microbiol.* **143**, 17–25 (2010).

156. Makkonen, J. *et al.* *Fusarium avenaceum* causes burn spot disease syndrome in noble crayfish (*Astacus astacus*). *J. Invertebr. Pathol.* **113**, 184–190 (2013).

157. Batta, Y. A. The first report on entomopathogenic effect of *Fusarium avenaceum* (Fries) Saccardo (Hypocreales, Ascomycota) against rice weevil (*Sitophilus oryzae* L.: Curculionidae, Coleoptera). *J. Entomol. Acarol. Res.* **44**, e11 (2012).

158. Jankowiak, R., Bilański, P., Paluch, J. & Kołodziej, Z. Fungi associated with dieback of *Abies alba* seedlings in naturally regenerating forest ecosystems. *Fungal Ecol.* **24**, 61–69 (2016).

159. Rivedal, H. M., Stone, A. G., Severns, P. M. & Johnson, K. B. Characterization of the Fungal Community Associated with Root, Crown, and Vascular Symptoms in an Undiagnosed Yield Decline of Winter Squash. *Phytobiomes J.* **4**, 178–192 (2020).

160. Okane, I. & Nakagiri, A. Assemblages of endophytic fungi on *Salicornia europaea* disjunctively distributed in Japan: towards clarification of the ubiquity of fungal endophytes on halophytes and their ecological roles. *Curr. Sci.* **109**, 62–71 (2015).

161. Corredor, A. H., Van Rees, K. & Vujanovic, V. Changes in root-associated fungal assemblages within newly established clonal biomass plantations of *Salix* spp. *For. Ecol. Manage.* **282**, 105–114 (2012).

162. Zhao, Z. *et al.* First Report of Fruit Blotch on Kiwifruit Caused by *Fusarium avenaceum* in China. *Plant Dis.* **104**, (2020).

163. Moslemi, A., Ades, P. K., Groom, T., Nicolas, M. E. & Taylor, P. W. J. *Fusarium oxysporum* and *Fusarium avenaceum* associated with yield-decline of pyrethrum in Australia. *Eur. J. Plant Pathol.* **149**, 43–56 (2017).

164. Zhou, Q. *et al.* Genetic diversity and aggressiveness of *Fusarium* species isolated from soybean in Alberta, Canada. *Crop Prot.* **105**, 49–58 (2018).

165. Wei, M. *et al.* First Report of *Fusarium avenaceum* Causing Root Rot of Maca (*Lepidium meyenii*) in China. *Plant Dis.* **101**, (2017).

166. Holtz, M. D., Chang, K. F., Hwang, S. F., Gossen, B. D. & Strelkov, S. E. Characterization of *Fusarium avenaceum* from lupin in central Alberta: Genetic diversity, mating type and aggressiveness. *Can. J. Plant Pathol.* **33**, 61–76 (2011).

167. Feng, J. *et al.* Genetic variation in *Fusarium avenaceum* causing root rot on field pea. *Plant Pathol.* **59**, 845–852 (2010).

168. Akita, M., Lehtonen, M. T., Koponen, H., Marttinen, E. M. & Valkonen, J. P. T. Infection of the Sunagoke moss panels with fungal pathogens hampers sustainable greening in urban environments. *Sci. Total Environ.* **409**, 3166–3173 (2011).

169. Lygis, V., Vasiliauskaite, I., Matelis, A., Pliūra, A. & Vasaitis, R. Fungi in living and dead stems and stumps of *Pinus mugo* on coastal dunes of the Baltic Sea. *Plant Prot. Sci.* **50**, 221–226 (2014).

170. Hujslová, M., Kubátová, A., Chudíčková, M. & Kolařík, M. Diversity of fungal communities in saline and acidic soils in the Soos National Natural Reserve, Czech Republic. *Mycol. Prog.* **9**, 1–15 (2010).

171. O’Donnell, K., Cigelnik, E. & Nirenberg, Helgard, I. Molecular systematics and phylogeography of the *Gibberella fujikuroi* species complex. *Mycologia* **90**, 465–493 (1998).

172. Mohamed Nor, N. M. I., Salleh, B. & Leslie, J. F. *Fusarium* species from Sorghum in Thailand. *Plant Pathol. J.* **35**, 301–312 (2019).

173. Laraba, I. *et al.* *Fusarium algeriense*, sp. nov., a novel toxigenic crown rot pathogen of durum wheat from Algeria is nested in the *Fusarium burgessii* species complex. *Mycologia* **109**, 935–950 (2017).

174. Okello, P. N., Petrovic, K., Singh, A. K., Kontz, B. & Mathew, F. M. Characterization of species of *Fusarium* causing root rot of Soybean (*Glycine max* L.) in South Dakota, USA. *Can. J. Plant Pathol.* **42**, 560–571 (2020).

175. Rodrigues, K. F. The foliar fungal endophytes of the Amazonian palm *Euterpe oleracea*. *Mycologia* **86**, 376–385 (1994).

176. Summerell, B. A. *et al.* *Fusarium* species associated with plants in Australia. *Fungal Divers.* **46**, 1–27 (2011).

177. Shimada, A., Fujioka, S., Koshino, H. & Kimura, Y. Nematicidal activity of beauvericin produced by the fungus *Fusarium bulbicola*. *Zeitschrift fur Naturforsch. - Sect. C J. Biosci.* **65**, 207–210 (2010).

178. Swett, C. L. & Gordon, T. R. Endophytic association of the pine pathogen *Fusarium circinatum* with corn (*Zea mays*). *Fungal Ecol.* **13**, 120–129 (2015).

179. Isaac, M. R. *et al.* Occurrence, identification, and pathogenicity of *Fusarium* spp. associated with tomato wilt in Mexico. *Not. Bot. Horti Agrobot. Cluj-Napoca* **46**, 484–493 (2018).

180. NCBI. *Fusarium coffeatum* FIESC_28. Available at: https://www.ncbi.nlm.nih.gov/biosample/SAMN08667433/. (Accessed: 19th May 2021)

181. Ferreira, M. C., de Assis, J. C. S. & Rosa, L. H. Diversity of endophytic fungi associated with *Carapichea ipecacuanha* from a native fragment of the Atlantic Rain Forest. *South African J. Bot.* **134**, 225–229 (2020).

182. Gerlach, W. Drei neue Varietäten von *Fusarium merismoides*, *F. larvarum* und *F. chlamydosporum*. *J. Phytopathol.* **90**, 31–42 (1977).

183. Laurence, M. H. *et al.* Six novel species of *Fusarium* from natural ecosystems in Australia. *Fungal Divers.* **77**, 349–366 (2016).

184. De Boer, W. *et al.* Anti-fungal properties of chitinolytic dune soil bacteria. *Soil Biol. Biochem.* **30**, 193–203 (1997).

185. Bolaños, J. *et al.* Phylogenetic Diversity of Sponge-Associated Fungi from the Caribbean and the Pacific of Panama and Their In Vitro Effect on Angiotensin and Endothelin Receptors. *Mar. Biotechnol.* **17**, 533–564 (2015).

186. Juybari, H. Z., Tajick Ghanbary, M. A., Rahimian, H., Karimi, K. & Arzanlou, M. Seasonal, tissue and age influences on frequency and biodiversity of endophytic fungi of *Citrus sinensis* in Iran. *For. Pathol.* **49**, e12559 (2019).

187. Rodriguez, R. J. *et al.* Stress tolerance in plants via habitat-adapted symbiosis. *ISME J.* **2**, 404–416 (2008).

188. Grunden, E., Chen, W. & Crane, J. L. Fungi colonizing microsclerotia of *Verticillium dahliae* in urban environments. *Fungal Divers.* **8**, 129–141 (2001).

189. de Rooij-van der Goes, P. C. E. M., van der Putten, W. H. & van Dijk, C. Analysis of nematodes and soil-borne fungi from *Ammophila arenaria* (Marram grass) in Dutch coastal foredunes by multivariate techniques. *Eur. J. Plant Pathol.* **101**, 149–162 (1995).

190. Chen, Y., Zhou, Q., Strelkov, S. E. & Hwang, S. F. Genetic diversity and aggressiveness of *Fusarium* spp. isolated from Canola in Alberta, Canada. *Plant Dis.* **98**, 727–738 (2014).

191. Backhouse, D. *et al.* Survey of *Fusarium* species associated with crown rot of wheat and barley in eastern Australia. *Australas. Plant Pathol.* **33**, 255–261 (2004).

192. Imathiu, S. M., Hare, M. C., Ray, R. V., Back, M. & Edwards, S. G. Evaluation of pathogenicity and aggressiveness of *F. langsethiae* on oat and wheat seedlings relative to known seedling blight pathogens. *Eur. J. Plant Pathol.* **126**, 203–216 (2010).

193. Stefańczyk, E., Sobkowiak, S., Brylińska, M. & Śliwka, J. Diversity of *Fusarium* spp. associated with dry rot of potato tubers in Poland. *Eur. J. Plant Pathol.* **145**, 871–884 (2016).

194. Khan, S. A. *et al.* Molecular diversity of halophilic fungi isolated from mangroves ecosystem of miani hor, Balochistan, Pakistan. *Pakistan J. Bot.* **52**, 1823–1829 (2020).

195. Potshangbam, M., Indira Devi, S., Sahoo, D. & Strobel, G. A. Functional characterization of endophytic fungal community associated with *Oryza sativa* L. and *Zea mays* L. *Front. Microbiol.* **8**, 325 (2017).

196. Proctor, R. H. *et al.* Evolution of structural diversity of trichothecenes, a family of toxins produced by plant pathogenic and entomopathogenic fungi. *PLoS Pathog.* **14**, e1006946 (2018).

197. NCBI. *Fusarium flagelliforme* NRRL 13405. Available at: https://www.ncbi.nlm.nih.gov/biosample/SAMN08631343/. (Accessed: 20th May 2021)

198. Xia, J. W., Sandoval-Denis, M., Crous, P. W., Zhang, X. G. & Lombard, L. Numbers to names – restyling the *Fusarium incarnatum-equiseti* species complex. *Persoonia* **43**, 186–221 (2019).

199. O’Donnell, K. *et al.* Novel multilocus sequence typing scheme reveals high genetic diversity of human pathogenic members of the *Fusarium incarnatum-F. equiseti* and *F. chlamydosporum* species complexes within the United States. *J. Clin. Microbiol.* **47**, 3851–3861 (2009).

200. CABI. GRC Catalogue. Available at: https://www.cabi.org/services/microbial-services/culture-collection-microorganism-supply/grc/. (Accessed: 3rd September 2021)

201. Wiemann, P. *et al.* Deciphering the Cryptic Genome: Genome-wide Analyses of the Rice Pathogen *Fusarium fujikuroi* Reveal Complex Regulation of Secondary Metabolism and Novel Metabolites. *PLoS Pathog.* **9**, e1003475 (2013).

202. Nisa, S. *et al.* Identification and Bioactivities of Two Endophytic Fungi *Fusarium fujikuroi* and *Aspergillus tubingensis* from Foliar Parts of *Debregeasia salicifolia*. *Arab. J. Sci. Eng.* **45**, 4477–4487 (2020).

203. Chang, X. *et al.* Identification of *Fusarium* species associated with soybean root rot in Sichuan Province, China. *Eur. J. Plant Pathol.* **151**, 563–577 (2018).

204. Mazarotto, E. J. *et al.* Pathogenic *Fusarium* species complexes associated to seeds of indigenous Brazilian forest tree *Aspidosperma polyneuron*. *Eur. J. Plant Pathol.* **158**, 849–857 (2020).

205. Chen, J. *et al.* First Report of *Fusarium fujikuroi* Causing Black Rot of *Bletilla striata* (Baiji) in China. *Plant Dis.* **103**, (2019).

206. Jiang, S. B. *et al.* First Report of *Fusarium fujikuroi* Causing Stem Wilt on *Canna edulis* Ker in China. *Plant Dis.* **102**, (2018).

207. Kim, B. R. & Choi, Y. J. *Fusarium fujikuroi* Causing Fusarium Wilt of *Lactuca serriola* in Korea. *Plant Dis.* **105**, 502 (2021).

208. Shen, Y. N. *et al.* First Report of Leaf Spot on *Lasia spinosa* Caused by *Fusarium fujikuroi* in China. *Plant Dis.* **104**, (2020).

209. Long, H. *et al.* First Report of Fruit Blotch on Plum Caused by *Fusarium fujikuroi* in China. *Plant Dis.* 1–2 (2021). doi:10.1094/PDIS-08-20-1784-PDN

210. Awan, U. A. *et al.* Isolation, fermentation, and formulation of entomopathogenic fungi virulent against adults of *Diaphorina citri*. *Pest Manag. Sci.* **77**, 4040–4053 (2021).

211. Phan, H. T. *et al.* *Gibberella gaditjirrii* (*Fusarium gaditjirrii*) sp. nov., a new species from tropical grasses in Australia. *Stud. Mycol.* **50**, 261–272 (2004).

212. Rheeder, J. P., Marasas, W. F. O. & Nelson, P. E. *Fusarium globosum*, a new species from corn in southern Africa. *Mycologia* **88**, 509–513 (1996).

213. Heydari-Nezhad, A. M., Babaeizad, V., Mirhosseini, H. A. & Khaksari, M. First report of a disease caused by *Fusarium globosum* on giant cane in Iran. *J. Plant Pathol.* **96**, S4.129 (2014).

214. Gagkaeva, T. Y., Gavrilova, O. P. & Orina, A. S. First report of *Fusarium globosum* associated with barley grain in the southwestern part of Siberia. *Plant Dis.* **103**, 588 (2019).

215. Trail, F. & Common, R. Perithecial development by *Gibberella zeae*: a light microscopy study. *Mycologia* **92**, 130–138 (2000).

216. Akbar, A., Hussain, S., Ullah, K., Fahim, M. & Ali, G. S. Detection, virulence and genetic diversity of *Fusarium* species infecting tomato in Northern Pakistan. *PLoS One* **13**, e0203613 (2018).

217. Lee, S. *et al.* Fungal Diversity and Enzyme Activity Associated with the Macroalgae, *Agarum clathratum*. *Mycobiology* **47**, 50–58 (2019).

218. Starkey, D. E. *et al.* Global molecular surveillance reveals novel Fusarium head blight species and trichothecene toxin diversity. *Fungal Genet. Biol.* **44**, 1191–1204 (2007).

219. Goswami, R. S. & Kistler, H. C. Heading for disaster: *Fusarium graminearum* on cereal crops. *Mol. Plant Pathol.* **5**, 515–525 (2004).

220. Scruggs, A. C. & Quesada-Ocampo, L. M. Etiology and epidemiological conditions promoting fusarium root rot in sweetpotato. *Phytopathology* **106**, 909–919 (2016).

221. Pak, D., You, M. P., Lanoiselet, V. & Barbetti, M. J. Reservoir of cultivated rice pathogens in wild rice in Australia. *Eur. J. Plant Pathol.* **147**, 295–311 (2017).

222. Li, Z. Y. *et al.* First Report of *Fusarium graminearum* Causing Ear Rot of Foxtail Millet in China. *Plant Dis.* **99**, (2015).

223. CBS-KNAW. *Fusarium heterosporum*. Available at: https://wi.knaw.nl/page/fungal_display/27411. (Accessed: 25th March 2021)

224. Ali, H., Backhouse, D. & Burgess, L. W. *Fusarium heterosporum* associated with paspalum ergot in eastern Australia. *Australas. Plant Pathol.* **25**, 120–125 (1996).

225. Torp, M. & Adler, A. The European *Sporotrichiella* project: a polyphasic approach to the biology of a new *Fusarium* species. *Int. J. Food Microbiol.* **95**, 241–245 (2004).

226. NCBI. *Fusarium longipes* NRRL 20695. Available at: https://www.ncbi.nlm.nih.gov/biosample/SAMN08631279/. (Accessed: 21st May 2021)

227. Maryani, N., Sandoval-Denis, M., Lombard, L., Crous, P. W. & Kema, G. H. J. New endemic *Fusarium* species hitch-hiking with pathogenic *Fusarium* strains causing Panama disease in small-holder banana plots in Indonesia. *Persoonia Mol. Phylogeny Evol. Fungi* **43**, 48–69 (2019).

228. Navi, S. S. & Singh, S. D. *Fusarium longipes*: a mycoparasite of *Sclerospora graminicola* on pearl millet. *Indian Phytopathol.* **46**, 365–368 (1993).

229. Besharati Fard, M., Mohammadi, A. & Darvishnia, M. *Fusarium* species associated with Wheat crown and root tissues in the Eastern Iran. *Arch. Phytopathol. Plant Prot.* **50**, 123–133 (2017).

230. Britz, H. *et al.* Two new species of *Fusarium* section Liseola associated with mango malformation. *Mycologia* **94**, 722–730 (2002).

231. Otero-Colina, G. *et al.* Identification and characterization of a novel etiological agent of mango malformation disease in Mexico, *Fusarium mexicanum* sp. nov. *Phytopathology* **100**, 1176–1184 (2010).

232. Santillán-Mendoza, R. *et al.* A novel disease of big-leaf mahogany caused by two *Fusarium* species in Mexico. *Plant Dis.* **102**, 1965–1972 (2018).

233. Al Yazidi, L. S. *et al.* Endobronchial fusariosis in a child following bilateral lung transplant. *Med. Mycol. Case Rep.* **23**, 77–80 (2019).

234. Marasas, W. F. O. *et al.* *Fusarium napiforme*, a New Species from Millet and Sorghum in Southern Africa. *Mycologia* **79**, 910 (1987).

235. Nelson, P. E., Plattner, R. D., Shackelford, D. D. & Desjardins, A. E. Fumonisin B1 Production by *Fusarium* Species Other Than *F. moniliforme* in Section Liseola and by Some Related Species. *Appl. Environ. Microbiol.* **58**, 984–989 (1992).

236. Supratman, U. *et al.* New naphthoquinone derivatives from *Fusarium napiforme* of a mangrove plant. *Nat. Prod. Res.* (2019). doi:10.1080/14786419.2019.1650358

237. Nucci, M. & Anaissie, E. *Fusarium* Infections in Immunocompromised Patients. *Clin. Microbiol. Rev.* **20**, 695–704 (2007).

238. NCBI. *Fusarium nygamai*. Available at: https://www.ncbi.nlm.nih.gov/biosample/SAMN06240347/. (Accessed: 25th March 2021)

239. Bogner, C. W. *et al.* Fungal root endophytes of tomato from Kenya and their nematode biocontrol potential. *Mycol. Prog.* **15**, 30 (2016).

240. Krulder, J. W. M., Brimicombe, R. W., Wijermans, P. W. & Gams, W. Systemic *Fusarium nygamai* infection in a patient with lymphoblastic non-Hodgkin’s lymphoma. *Mycoses* **39**, 121–123 (1996).

241. Burgess, L. W. & Trimboli, D. Characterization and Distribution of *Fusarium nygamai*, sp. nov. *Mycologia* **78**, 223–229 (1986).

242. Balmas, V., Corda, P., Marcello, A. & Bottalico, A. *Fusarium nygamai* Associated with Fusarium Foot Rot of Rice in Sardinia. *Plant Dis.* **84**, (2000).

243. Leslie, J. F., Zeller, K. A., Lamprecht, S. C., Rheeder, J. P. & Marasas, W. F. O. Toxicity, pathogenicity, and genetic differentiation of five species of *Fusarium* from sorghum and millet. *Phytopathology* **95**, 275–283 (2005).

244. Azil, N. *et al.* Identification and pathogenicity of *Fusarium* spp. associated with tuber dry rot and wilt of potato in Algeria. *Eur. J. Plant Pathol.* **159**, 495–509 (2021).

245. Sauerborn, J. *et al.* *Striga hermonthica* control with *Fusarium nygamai* in maize. in *Proceedings of the IX International Symposium on Biological Control of Weeds* (eds. Moran, V. C. & Hoffmann, J. H.) 461–466 (1996).

246. Guo, L. *et al.* Genome and transcriptome analysis of the fungal pathogen *Fusarium oxysporum* f. sp. *cubense* causing banana vascular wilt disease. *PLoS One* **9**, e95543 (2014).

247. Delulio, G. A. *et al.* Kinome Expansion in the *Fusarium oxysporum* Species Complex Driven by Accessory Chromosomes. *mSphere* **3**, e00231-18 (2018).

248. NCBI. *Fusarium oxysporum* f. sp. *cepae* FoC_Fus2. Available at: https://www.ncbi.nlm.nih.gov/biosample/SAMN05529097/. (Accessed: 20th May 2021)

249. Armitage, A. D. *et al.* Characterisation of pathogen-specific regions and novel effector candidates in *Fusarium oxysporum* f. sp. *cepae*. *Sci. Rep.* **8**, 13530 (2018).

250. Abawi, G. S. & Lorbeer, J. W. Several Aspects of the Ecology and Pathology of *Fusarium oxysporum* f. sp. *cepae*. *Phytopathology* **62**, 870 (1972).

251. Leoni, C., de Vries, M., ter Braak, C. J. F., van Bruggen, A. H. C. & Rossing, W. A. H. *Fusarium oxysporum* f.sp. *cepae* dynamics: in-plant multiplication and crop sequence simulations. *Eur. J. Plant Pathol.* **137**, 545–561 (2013).

252. Thatcher, L. F., Gardiner, D. M., Kazan, K. & Manners, J. M. A highly conserved effector in *Fusarium oxysporum* is required for full virulence on *Arabidopsis*. *Mol. Plant-Microbe Interact.* **25**, 180–190 (2012).

253. Ma, L. J. *et al.* Comparative genomics reveals mobile pathogenicity chromosomes in *Fusarium*. *Nature* **464**, 367–373 (2010).

254. Menzies, J. G., Koch, C. & Seywerd, F. Additions to the Host Range of *Fusarium oxysporum* f. sp. *radicis-lycopersici*. *Plant Dis.* **74**, 569–572 (1990).

255. Kishi, K., Furukawa, T. & Aoki, T. Purple Spot of Aloe (*Aloe arborescens* Mill.) Caused by *Fusarium phyllophilum* Nirenberg et O’Donnell (New Disease). *Japanese J. Phytopathol.* **65**, 576–587 (1999).

256. Vanheule, A. *et al.* Living apart together: crosstalk between the core and supernumerary genomes in a fungal plant pathogen. *BMC Genomics* **17**, 670 (2016).

257. Stenglein, A. S. A. *Fusarium poae*: a pathogen that needs more attention. *J. Plant Pathol.* **91**, 25–36 (2009).

258. Yilmaz, N. *et al.* Redefining species limits in the *Fusarium fujikuroi* species complex. *Persoonia* **46**, 129–162 (2021).

259. Nirenberg, H. I. *et al.* Two new species of *Fusarium*: *Fusarium brevicatenulatum* from the noxious weed *Striga asiatica* in Madagascar and *Fusarium pseudoanthophilum* from *Zea mays* in Zimbabwe. *Mycologia* **90**, 459–464 (1998).

260. Amobonye, A., Bhagwat, P., Ranjith, D., Mohanlall, V. & Pillai, S. Characterisation, pathogenicity and hydrolytic enzyme profiling of selected *Fusarium* species and their inhibition by novel coumarins. *Arch. Microbiol.* (2021). doi:10.1007/s00203-021-02335-1

261. Tupaki-Sreepurna, A. *et al.* Phylogenetic Diversity and *In Vitro* Susceptibility Profiles of Human Pathogenic Members of the *Fusarium fujikuroi* Species Complex Isolated from South India. *Mycopathologia* **183**, 529–540 (2018).

262. Cavalcanti, A. D. *et al.* *Fusarium massalimae* sp. nov. (*F. lateritium* species complex) occurs endophytically in leaves of *Handroanthus chrysotrichus*. *Mycol. Prog.* **19**, 1133–1142 (2020).

263. Nicolli, C. P. *et al.* *Fusarium fujikuroi* species complex in Brazilian rice: Unveiling increased phylogenetic diversity and toxigenic potential. *Int. J. Food Microbiol.* **330**, 108667 (2020).

264. Shiraishi, A., Leslie, J. F., Zhong, S. & Uchida, J. Y. AFLP, Pathogenicity, and VCG Analyses of *Fusarium oxysporum* and *Fusarium pseudocircinatum* from *Acacia koa*. *Plant Dis.* **96**, 1111–1117 (2012).

265. Gardiner, D. M. *et al.* Comparative Pathogenomics Reveals Horizontally Acquired Novel Virulence Genes in Fungi Infecting Cereal Hosts. *PLoS Pathog.* **8**, e1002952 (2012).

266. Aoki, T. & O’Donnell, K. Morphological and molecular characterization of *Fusarium pseudograminearum* sp. nov., formerly recognized as the Group 1 population of *F. graminearum*. *Mycologia* **91**, 597–609 (1999).

267. NCBI. *Fusarium sarcochroum* NRRL 20472. Available at: https://www.ncbi.nlm.nih.gov/biosample/SAMN13683633/. (Accessed: 20th May 2021)

268. Sandoval-Denis, M., Guarnaccia, V., Polizzi, G. & Crous, P. W. Symptomatic citrus trees reveal a new pathogenic lineage in *Fusarium* and two new *Neocosmospora* species. *Persoonia* **40**, 1–25 (2018).

269. NCBI. *Fusarium sporotrichioides* NRRL 3299. Available at: https://www.ncbi.nlm.nih.gov/biosample/SAMN08631227/. (Accessed: 1st September 2021)

270. Moya-Elizondo, E. A., Arismendi, N., Montalva, C. & Doussoulin, H. First Report of *Fusarium sporotrichioides* Causing Foliar Spots on Forage Corn in Chile. *Plant Dis.* **97**, (2013).

271. NCBI. *Fusarium subglutinans* NRRL 66333. Available at: https://www.ncbi.nlm.nih.gov/biosample/SAMN13683616/. (Accessed: 1st September 2021)

272. Ruan, R. *et al.* First report of *Fusarium subglutinans* causing heart rot on *Cymbidium hybridum* in China. *Crop Prot.* **144**, 105603 (2021).

273. ATCC. *Fusarium venenatum* Nirenberg (ATCC® 20334^TM^). Available at: https://www.lgcstandards-atcc.org/products/all/20334.aspx?geo_country=gb#history. (Accessed: 21st May 2021)

274. Tan, D. C. *et al.* Mycotoxins produced by *Fusarium* species associated with annual legume pastures and ‘sheep feed refusal disorders’ in Western Australia. *Mycotoxin Res.* **27**, 123–135 (2011).

275. Mycocosm. *Fusarium verticillioides*. Available at: https://mycocosm.jgi.doe.gov/Fusve2/Fusve2.home.html. (Accessed: 25th March 2021)

276. Salem, N. M., Almomany, A. M., Tahat, M. M. & Aldakil, H. First Report of *Fusarium verticillioides* Causing Banana Fruit Rot in Jordan. *Plant Dis.* **104**, 3255 (2020).

277. Hilton, A., Zhang, H., Yu, W. & Shim, W. B. Identification and characterization of pathogenic and endophytic fungal species associated with pokkah boeng disease of sugarcane. *Plant Pathol. J.* **33**, 238–248 (2017).

278. Marín, P., Palmero, D. & Jurado, M. Occurrence of moulds associated with ovine raw milk and cheeses of the Spanish region of Castilla La Mancha. *Int. J. Dairy Technol.* **68**, 565–572 (2015).

279. NCBI. *Fusarium zealandicum* NRRL 22465. Available at: https://www.ncbi.nlm.nih.gov/biosample/SAMN13683635/. (Accessed: 20th May 2021)

280. Schroers, H. J., Gräfenhan, T., Nirenberg, H. I. & Seifert, K. A. A revision of *Cyanonectria* and *Geejayessia* gen. nov., and related species with *Fusarium*-like anamorphs. *Stud. Mycol.* **68**, 115–138 (2011).

281. Liao, H. L. *et al.* Fungal endophytes of *Populus trichocarpa* alter host phenotype, gene expression, and rhizobiome composition. *MPMI* **32**, 853–864 (2019).

282. Short, D. P. G. *et al.* PCR multiplexes discriminate *Fusarium* symbionts of invasive *Euwallacea* ambrosia beetles that inflict damage on numerous tree species throughout the United States. *Plant Dis.* **101**, 233–240 (2017).

283. O’Donnell, K. *et al.* Discordant phylogenies suggest repeated host shifts in the *Fusarium-Euwallacea* ambrosia beetle mutualism. *Fungal Genet. Biol.* **82**, 277–290 (2015).

284. Freeman, S. *et al.* *Fusarium euwallaceae* sp. nov.-a symbiotic fungus of *Euwallacea* sp., an invasive ambrosia beetle in Israel and California. *Mycologia* **105**, 1595–1606 (2013).

285. Eskalen, A. *et al.* Host range of Fusarium dieback and its ambrosia beetle (Coleoptera: Scolytinae) vector in southern California. *Plant Dis.* **97**, 938–951 (2013).

286. Aoki, T. *et al.* Three novel Ambrosia *Fusarium* Clade species producing clavate macroconidia known (*F. floridanum* and *F. obliquiseptatum*) or predicted (*F. tuaranense*) to be farmed by *Euwallacea* spp. (Coleoptera: Scolytinae) on woody hosts. *Mycologia* **111**, 919–935 (2019).

287. NCBI. *Fusarium kuroshium* UCR3666. Available at: https://www.ncbi.nlm.nih.gov/biosample/SAMN07200645. (Accessed: 20th May 2021)

288. Na, F. *et al.* Two novel fungal symbionts *Fusarium kuroshium* sp. nov. and *Graphium kuroshium* sp. nov. of kuroshio shot hole borer (*Euwallacea* sp. nr. *fornicatus*) cause fusarium dieback on woody host species in California. *Plant Dis.* **102**, 1154–1164 (2018).

289. Coleman, J. J. *et al.* The genome of *Nectria haematococca*: Contribution of supernumerary chromosomes to gene expansion. *PLoS Genet.* **5**, e1000618 (2009).

290. Šišić, A. *et al.* The ‘forma specialis’ issue in *Fusarium*: A case study in *Fusarium solani* f. sp. *pisi*. *Sci. Rep.* **8**, 1252 (2018).

291. Aoki, T. *et al.* Three novel Ambrosia *Fusarium* Clade species producing multiseptate ‘dolphin-shaped’ conidia, and an augmented description of *Fusarium kuroshium*. *Mycologia* **113**, 1089–1109 (2021).

1. Previously classified as *F. proliferatum*, which was epitypified ^258^ [↑](#footnote-ref-2)
2. Only pathogenised on detached and mostly wounded leaves, latent saprotroph? [↑](#footnote-ref-3)
3. Presumed from original description^106^, in absence of associated data. [↑](#footnote-ref-4)
4. ‘third generation cross between two field isolates: one (T2) obtained from a infected pea plant in NY and the other (T219) obtained from soil in a potato field in PA’ [↑](#footnote-ref-5)
